# Quantitative super-resolution single molecule microscopy dataset of YFP-tagged growth factor receptors

**DOI:** 10.1093/gigascience/giy002

**Published:** 2018-01-19

**Authors:** Tomáš Lukeš, Jakub Pospíšil, Karel Fliegel, Theo Lasser, Guy M Hagen

**Affiliations:** 1 Laboratoire d’Optique Biomédicale, École Polytechnique Fédérale de Lausanne, Route Cantonale, CH-1015 Lausanne, Switzerland; 2 Department of Radioelectronics, Faculty of Electrical Engineering, Czech Technical University in Prague, Technická 2, 16627 Prague 6, Czech Republic; 3 UCCS center for the Biofrontiers Institute, University of Colorado at Colorado Springs, 1420 Austin Bluffs Parkway, Colorado Springs, Colorado, 80918, USA

**Keywords:** super-resolution microscopy, PALM, STORM, SOFI, YFP, SMLM, single molecule, growth factor receptor, ErbB3, ThunderSTORM

## Abstract

**Background:**

Super-resolution single molecule localization microscopy (SMLM) is a method for achieving resolution beyond the classical limit in optical microscopes (approx. 200 nm laterally). Yellow fluorescent protein (YFP) has been used for super-resolution single molecule localization microscopy, but less frequently than other fluorescent probes. Working with YFP in SMLM is a challenge because a lower number of photons are emitted per molecule compared with organic dyes, which are more commonly used. Publically available experimental data can facilitate development of new data analysis algorithms.

**Findings:**

Four complete, freely available single molecule super-resolution microscopy datasets on YFP-tagged growth factor receptors expressed in a human cell line are presented, including both raw and analyzed data. We report methods for sample preparation, for data acquisition, and for data analysis, as well as examples of the acquired images. We also analyzed the SMLM datasets using a different method: super-resolution optical fluctuation imaging (SOFI). The 2 modes of analysis offer complementary information about the sample. A fifth single molecule super-resolution microscopy dataset acquired with the dye Alexa 532 is included for comparison purposes.

**Conclusions:**

This dataset has potential for extensive reuse. Complete raw data from SMLM experiments have typically not been published. The YFP data exhibit low signal-to-noise ratios, making data analysis a challenge. These datasets will be useful to investigators developing their own algorithms for SMLM, SOFI, and related methods. The data will also be useful for researchers investigating growth factor receptors such as ErbB3.

## Data Description

### Context

Fluorescence optical microscopy is one of the most important tools available for the study of biological systems at the cellular level. Unfortunately, due to diffraction phenomena, the resolution of fluorescence microscopes in the lateral *d*dimension is limited to

(1) \begin{equation*}d = \frac{{0.61\lambda }}{{NA}},\end{equation*}

where λ is the wavelength of the detected light and NA is the numerical aperture of the objective lens. As many biological structures within cells are much smaller than this, increasing resolution is of prime importance. Today several methods have been developed that are able to image below the diffraction limit [1, 2].

Photoactivated localization microscopy (PALM) [3] was initially accomplished with the photoconvertible fluorescent protein mEOS [4]. A similar method, (direct) stochastic optical reconstruction microscopy ((d)STORM) utilizes organic dyes [5–8]. In these super-resolution methods, single fluorescent molecules are induced to blink on and off (photoswitching) randomly in the sample. A sensitive camera is used to record an image sequence of the single molecule blinking events, and a computational algorithm is used to fit the imaged point spread functions (PSFs) to a model function [9, 10]. By doing so, the coordinates of each molecule can be determined with an uncertainty that is below the diffraction limit [11]. Once enough molecules have been imaged (usually 10^6^–10^7^ are required, depending on the sample structure) [12], an image can be reconstructed with lateral resolution improved by about a factor of 10. This is done by plotting the coordinates of each molecule in a new image with a much smaller pixel size. Together, this family of methods is known as single molecule localization microscopy (SMLM).

Although PALM experiments were initially performed with fluorescent proteins that are specifically photoconvertible [3], green fluorescent protein (GFP) and its spectral variant yellow fluorescent protein (YFP) are also known to exhibit blinking characteristics [13]. GFP and YFP have been used in SMLM, but less frequently [14–20]. Here we used a modified YFP known as mCitrine [21] for SMLM. The advantage of using mCitrine is that SMLM can be accomplished with a single laser, rather than with separate activation and readout lasers, as is done when using mEOS [3]. The question of how fluorophore photophysics influences SMLM experiments is still under investigation [22], but this topic has recently been reviewed fairly comprehensively, taking into account the photoswitching characteristics of fluorescent proteins for SMLM [23].

We used mCitrine to perform SMLM of the growth factor ErbB3 in A431 epithelial carcinoma cells. A431 cells were chosen for this study in part because of their use in previous studies of the ErbB receptor system [24, 25], and also because they tend to be very flat and form extended areas of membrane in contact with the coverslip, offering good conditions for SMLM. ErbB3 is a member of the epidermal growth factor receptor (EGFR) family, consisting of ErbB1 (EGFR), ErbB2 (also known as HER2), ErbB3, and ErbB4. The organization and dynamics of ErbB receptors is an important topic of study because overexpression and unrestrained activation of this family of receptors is implicated in cancer [26], including breast cancer [27]. Long thought to have no kinase activity, ErbB3 has recently been found to exhibit tyrosine kinase activity and to form homodimers and heterodimers with other ErbB receptors [28]. Such heterodimer formation between ErbB molecules can amplify signaling and appears to be an important feature of some cancer cells. In particular, the ErbB2/ErbB3 heterodimer appears to be important for tumor cell proliferation in certain breast cancers [29]. High ErbB3 levels have been linked to resistance in cancer therapies that target ErbB1 or ErbB2 [30].

Given the importance of ErbB3 in cancer, an understanding of its organization and dynamics in the plasma membrane of tumor cells is critical. Super-resolution microscopy using single molecule localization reveals the coordinates of each ErbB3 receptor, which is tagged with a YFP molecule. These data allow one to explore parameters such as clustering tendencies, an approach used successfully in studies of the T-cell receptor [31].

We have also included an additional single molecule super-resolution microscopy dataset acquired using the dye Alexa 532. This dye is more commonly used in (d)STORM studies [32] and is provided for purposes of comparison of the single molecule parameters. For this experiment, we used an Alexa 532-labeled antibody to detect RNA molecules in the nucleus of a HeLa cell, as previously described [33]. The raw data are useful in this context because they were acquired with the same microscope setup and detector. Compared with the YFP used in the other datasets, Alexa 532 has higher photon emission rates and exhibits less photobleaching.

The datasets have potential for extensive reuse. Complete raw data from SMLM experiments have typically not been published. The YFP data exhibit low signal-to-noise ratios, making data analysis a challenge. The datasets will be useful to investigators developing their own algorithms for SMLM, SOFI, and related methods. The data will also be useful for researchers investigating growth factor receptors such as ErbB3, as well as to those investigating other membrane proteins.

## Methods

### Cell lines and reagents

A431 cells (RRID: CVCL_0037) expressing mCitrine-ErbB3 and HeLa cells (RRID: CVCL_0030) were maintained in phenol red-free DMEM supplemented with 10% FCS, 100 U/ml penicillin, 100 U/ml streptomycin, and L-glutamate (obtained from Invitrogen, Carlsbad, CA, USA) at 37°C and 100% humidity. Mowiol 4–88 containing 1,4-diazabicyclo(2.2.2)octane (DABCO) was obtained from Fluka (St. Louis, MO, USA). Mercaptoethylamine (MEA) was obtained from Sigma (St. Louis, MO, USA).

### Sample preparation

Prior to SMLM experiments, A431 cells were grown on clean #1.5 coverslips for 12–18 hours. The cells were then washed with PBS and fixed with 4% paraformaldehyde for 15 minutes at 4°C. We then mounted the cells on clean slides using mowiol containing DABCO and 50–100 mM MEA, pH 8.5. Before microscopy, the mowiol was allowed to harden for 12–18 hours. The mowiol was freshly prepared according to standard procedures.

For labeling of transcription sites in the cell nucleus, HeLa cells were grown on #1.5 coverslips for 12–18 hours, then incubated for 5 minutes with 5-fluorouridine (Sigma) at a concentration of 10 μM. The cells were then fixed in 2% formaldehyde, permeabilized with 0.1% Triton X-100, and labeled using a mouse monoclonal anti-BrdU antibody (clone BU-33, Sigma). The anti-BrdU antibodies were then detected with a secondary antimouse antibody labeled with Alexa 532 (Invitrogen). The cells were mounted using freshly prepared mowiol containing DABCO and 50–100 mM MEA. Before microscopy, the mowiol was allowed to harden for 12–18 hours.

### Single molecule microscopy

For SMLM imaging, we used an IX71 microscope equipped with a planapochromatic 100 ×/1.35 NA oil immersion objective (Olympus, Tokyo, Japan) and a front-illuminated Ixon DU885 EMCCD camera under control of IQ software (Andor, Belfast, Northern Ireland), as previously described [34]. The excitation source was a 400-mW, 473-nm laser (Dragon laser, ChangChun, China), which was coupled to the microscope using a 0.39-NA multimode optical fiber. The fiber output was collimated using a 2-inch diameter, 60-mm FL lens (Thor Labs, Newton, NJ, USA). The fiber was coupled into the microscope using an Olympus IX2-RFAL fluorescence illuminator, resulting in an evenly illuminated field. Fluorescence was observed using an Olympus U-MNIBA3 filter set (excitation 470–495 nm, dichroic 505 nm, emission 510–550 nm). In each experiment, a sequence of 1419–10 000 images was acquired, with an exposure time of 40–100 ms and an EM gain of 50–300. For imaging Alexa 532, we used a 1-W, 532-nm laser (Dragon laser) and an appropriate fluorescence emission filter (569–610 nm, Chroma), as previously described [33].

### Data analysis methods

We analyzed the data using ThunderSTORM [9, 35] with the default settings. The default settings involve use of a wavelet-based filter for feature enhancement [36], followed by local maximum detection of single molecules in the filtered data. This is followed by fitting molecules in the raw data using a 2-dimensional Gaussian function in integrated form [37] using maximum likelihood methods [38]. Gaussian functions have been found to be a good representation of the true PSF of a microscope [39]. For visualization of the results, we use an average shifted histogram approach [40]. If the camera calibration parameters (pixel size, photoelectrons per A/D count, base level, and EM gain) are correct, maximum likelihood fitting of an integrated Gaussian function will correctly return the number of photons detected from each molecule [9, 37, 38, 41]. An integrated 2-dimensional Gaussian function can be written as

(2) \begin{eqnarray*} PS{F_{IG}}(x,y|\theta ) &=& {\theta _N}{E_x}{E_y} + {\theta _b},\nonumber\\ {E_x} &=& \frac{1}{2}{{\rm erf}}\left( {\frac{{x - {\theta _x} + {{1} / {2}}}}{{\sqrt 2 {\theta _\sigma }}}} \right) - \frac{1}{2}{{\rm erf}}\left( {\frac{{x - {\theta _x} - {{1}/{2}}}}{{\sqrt 2 {\theta _\sigma }}}} \right),\nonumber\\ {E_y} &=& \frac{1}{2}{{\rm erf}}\left( {\frac{{{\it y} - {\theta _{\it y}} + {{1}/{2}}}}{{\sqrt 2 {\theta _\sigma }}}} \right) - \frac{1}{2}{{\rm erf}}\left( {\frac{{y - {\theta _y} - {{1}/{2}}}}{{\sqrt 2 {\theta _\sigma }}}} \right), \end{eqnarray*}

where θ_*x*_, θ_*y*_ are the sub-pixel molecular coordinates, θ_σ_ is the standard deviation of the Gaussian function (i.e., the width), θ_*N*_ is the total number of detected photons emitted by the molecule, and θ_*b*_ is the background offset.

### Single molecule localization uncertainty

In ThunderSTORM, the localization uncertainty is calculated for each detected molecule. This quantity can help one determine whether the molecule was well localized and whether it should be included in the final result. Let ${\hat{\theta }_\sigma }$be the standard deviation of a Gaussian function fitted to an imaged PSF in nm, *a* is the back-projected pixel size in nm (camera pixel size divided by system magnification), ${\hat{\theta }_N}$is the estimate of the number of photons detected for a given molecule, and${\hat{\hphantom{\,}b}}$ is the background signal level in photons, calculated as the standard deviation of the residuals between the raw data and the fitted PSF model. The uncertainty of estimates determined by maximum likelihood methods for the lateral position of a molecule is given by

(3) \begin{eqnarray*} {\left( {\Delta {{\hat{\theta }}_{xy}}} \right)^2} &=& \frac{{g{{\hat{\theta }}_{{\sigma ^2}}} + {a^2}/12}}{{{{\hat{\theta }}_N}}}\left( {1 + 4\tau + \sqrt {\frac{{2\tau }}{{1 + 4\tau }}} } \right), \nonumber\\ \tau &=& \frac{{2\pi \left( {{{\hat{b}}^2} + r} \right)\left( {\hat{\theta }_\sigma ^2 + {a^2}/12} \right)}}{{{a^2}{{\hat{\theta }}_N}}}. \end{eqnarray*}

This formula is a modified form of the Thompson-Larson-Webb equation [11] and was derived by Rieger and Stallinga [42]. Finally, compensation for camera readout noise *r* and EM gain *g* was added following Quan, Zeng, and Huang [43], who suggested that when using EMCCD cameras, the correction factors should be set to *r* = 0, *g* = 2, and when using CCD or sCMOS cameras, the correction factors should be set to *r* = *g* = 2.

### Super-resolution optical fluctuation imaging

Super-resolution optical fluctuation imaging (SOFI) is based on calculation of spatio-temporal cumulants over the input sequence of camera frames [44]. Assuming a nonfluctuating background and Gaussian additive noise, the *n*th order cumulant (for *n* ≥ 2 and a time lag τ) can be written as

(4) \begin{equation*}{\kappa _n}\{ I({{\bf r}},t)\} (\tau ) = \sum\limits_{k = 1}^N {\varepsilon _k^n} {U^n}({{\bf r}} - {{{\bf r}}_k}){\kappa _n}\{ {s_k}(t)\} (\tau ),\end{equation*}

where *I*(**r**, *t*)is the detected intensity at position **r**and time *t*, ε_*k*_is the molecular brightness of *k*th emitter, *U^n^*(**r** − **r**_*k*_) is the PSF at the position **r**_*k*_, and *s_k_*(*t*) denotes a normalized fluctuation sequence *s_k_*(*t*) ∈ {0, 1}. The PSF is raised to the *n*th power, resulting in resolution increased by a factor of $\sqrt n $. After reweighting in frequency space, a resolution enhancement factor of *n* can be achieved [45], scaling linearly with the cumulant order. SOFI can be applied to any image sequence of stochastically blinking emitters acquired from a conventional widefield microscope if the emitters switch between at least 2 optically distinguishable states (a dark state and a bright state) and if sampling of the PSF fulfills the Nyquist–Shannon sampling theorem [46]. In comparison with STORM, SOFI tolerates higher densities of emitters and higher blinking rates [47], resulting in improved temporal resolution [48]. SOFI can be applied to the same datasets as SMLM analysis [47, 49], offering an interesting complement to SMLM methods. Due to the entirely different image processing methods used, SOFI and SMLM are prone to different artifacts. Applying both processing methods to the same dataset reveals more information about the true structure and properties of the underlying sample. By combining multiple orders of the SOFI analysis, molecular parameters like molecular density, brightness, and on-time ratio can be extracted using the balanced SOFI method (bSOFI) [50]. The on-time ratioρ_*on*_describes the blinking rate of the fluorescent label. Assuming a 2-state blinking model where the emitter fluctuates between a bright state and a dark state, the on-time ratio is given as [38]:

(5) \begin{equation*}{\rho _{on}} = \frac{{{\tau _{on}}}}{{{\tau _{on}} + {\tau _{off}}}},\end{equation*}

where τ_*on*_and τ_*off*_are the characteristic lifetimes of the bright state and the dark state, respectively.

SOFI analysis was carried out as reported previously [49]. We used a custom written algorithm (Matlab, The Mathworks) based on the code of our SOFI simulation tool [51] and the bSOFI algorithm [50]. The sequence of camera frames was divided into subsequences of 500 frames each. The subsequences were processed separately in order to minimize the influence of photobleaching, and the resulting SOFI images were averaged. Details about photobleaching correction for SOFI have recently been published [52]. SOFI relies on calculating higher-order cumulants, as described in the previous section. Calculating cumulants raises the molecular brightness to the *n*th power (Equation 3). SOFI’s nonlinear response to brightness becomes an issue for cumulants of higher than second order, where fluorescent spots of high brightness may mask less bright details. The balanced SOFI (bSOFI) algorithm linearizes the response to brightness [50] or to the detected intensity [49]. Throughout this work, the “*n*th-order bSOFI image” refers to an image calculated using the *n*th-order cumulant and applying the subsequent linearization according to the procedure described in Deschout et al. [49].

### Super-resolution images

**Figure 1:**
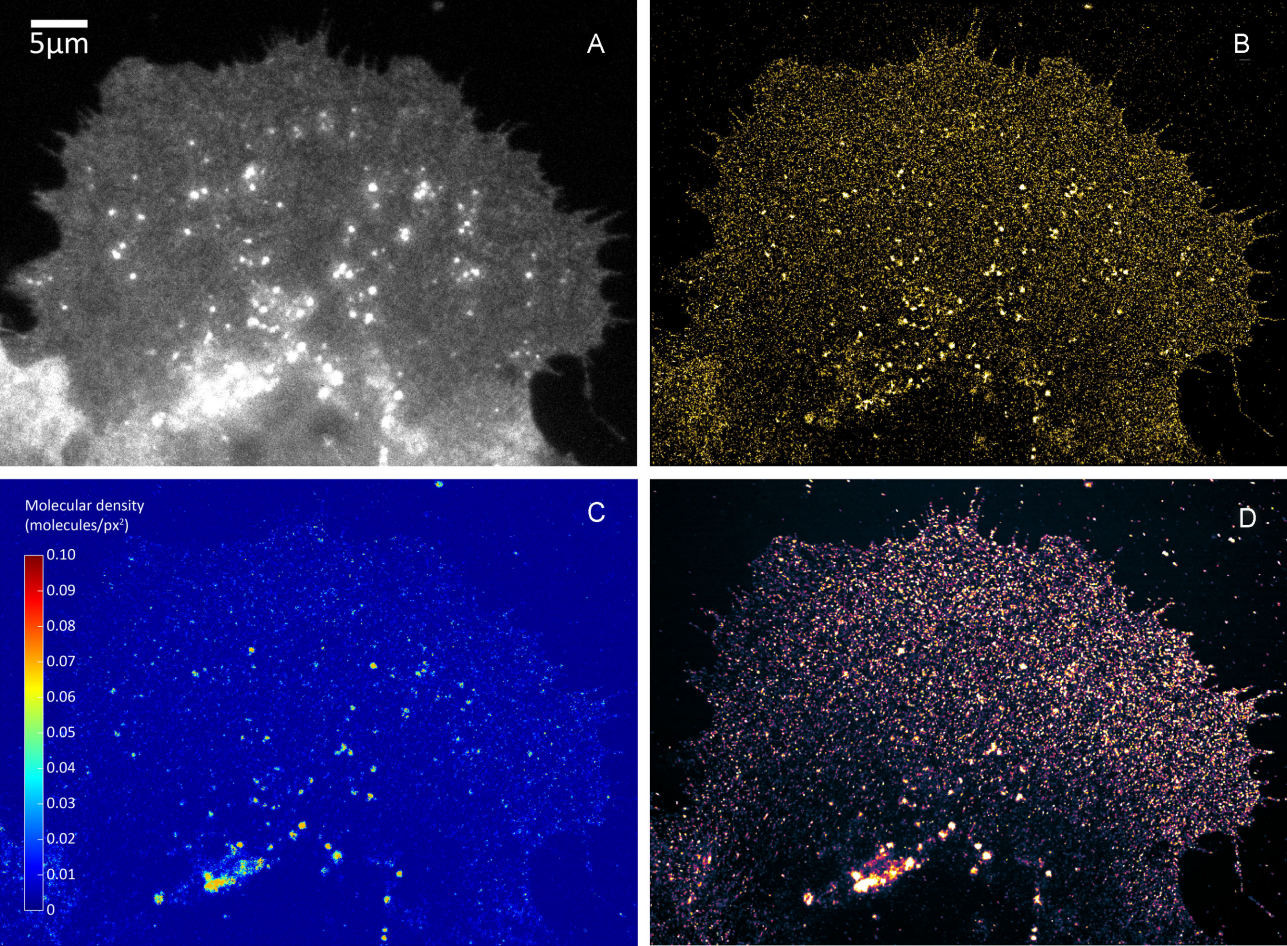
Super-resolution imaging of mCitrine-ErbB3 in A431 cells. (A) Conventional widefield. (B) SMLM. (C) Molecular density map. (D) Fourth-order bSOFI.

Figure 1 shows images of an A431 cell expressing mCitrine-ErbB3 (YFP dataset 1) [53]. Conventional widefield (WF) (Fig. 1A) and SMLM (Fig. 1B) results are shown. Figure 1C shows a color-coded density map, calculated by the bSOFI algorithm. This unique information cannot be obtained by conventional fluorescence microscopy. Figure 1D shows the fourth-order bSOFI image.

**Figure 2:**
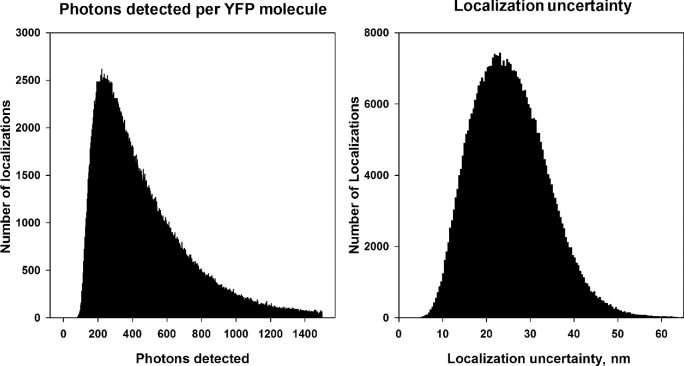
Quantification of molecular parameters from the experiment shown in Fig. 1. (A) Histogram of the number of photons detected from each YFP molecule. (B) Histogram of the localization uncertainty calculated for each YFP molecule.

Figure 2A shows a histogram of the number of photons detected from each YFP molecule (“intensity” in ThunderSTORM) for the cell shown in Fig. 1, and Fig. 2B shows a histogram of the localization uncertainty determined for each molecule for the cell shown in Fig. 1. The localization uncertainty was calculated using Equation 3. The 2 histograms were calculated using the plot histogram command in ThunderSTORM.

Table 1 shows a list of quantitative parameters for the first 10 detected molecules, as reported by ThunderSTORM for the experiment shown in Fig. 1. Sigma (nm) is the standard deviation of the 2-dimensional integrated Gaussian function fitted to the molecule, intensity (photons) is the number of photons detected from the molecule, offset (photons) is the background offset, SD of background (photons) is the standard deviation of the background, and localization uncertainty (nm) is the result of Equation 2 for each molecule. Recall that the full width at half max (FWHM) of a Gaussian function is related to its standard deviation by FWHM = 2.35σ. The variation in parameters between molecules is usually attributed to differences in the local environment of each molecule, such as oxygen concentration, and to factors such as the fluorophore orientation.

**Table 1:** Quantitative parameters for the first 10 detected molecules as reported by ThunderSTORM for the experiment shown in Fig. 1

Molecule number	Camera frame	x, nm	y, nm	Sigma, nm	Intensity, photons	Offset, photons	SD of background, photons	Localization uncertainty, nm
1	1	3743.17	28005.63	81.53	942	108	25	17.41
2	1	3880.95	31519.89	155.09	2014	68	21	23.33
3	1	4150.78	32662.21	60.03	433	81	21	17.75
4	1	4289.06	28407.32	36.90	407	155	32	12.97
5	1	4310.28	28737.99	103.00	1567	142	34	21.61
6	1	4615.06	23832.74	89.18	1186	73	22	14.60
7	1	4695.34	30060.77	102.05	1266	122	28	22.17
8	1	4812.40	30994.57	101.18	1051	115	24	22.61
9	1	4827.01	25960.59	83.02	717	80	20	19.35
10	1	5037.67	28686.08	149.32	2293	121	33	29.85

**Figure 3:**
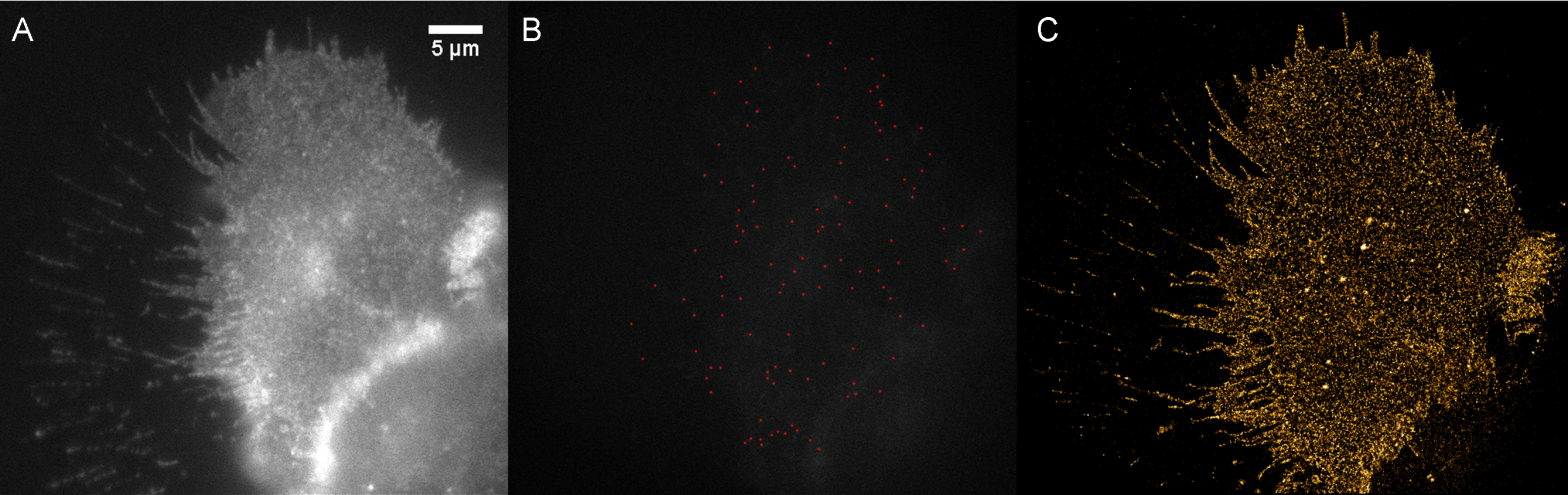
Super-resolution imaging of mCitrine-ErbB3 in A431 cells. (A) Conventional widefield. (B) Single frame of SMLM, with detections indicated with red dots. (C) SMLM reconstruction.

Figure 3 shows WF imaging of an A431 cell (Fig. 3A), along with identification of single molecules by ThunderSTORM (Fig. 3B, indicated by red dots, and the reconstructed SMLM result (YFP dataset 2) (Fig. 3C) [53].

**Figure 4:**
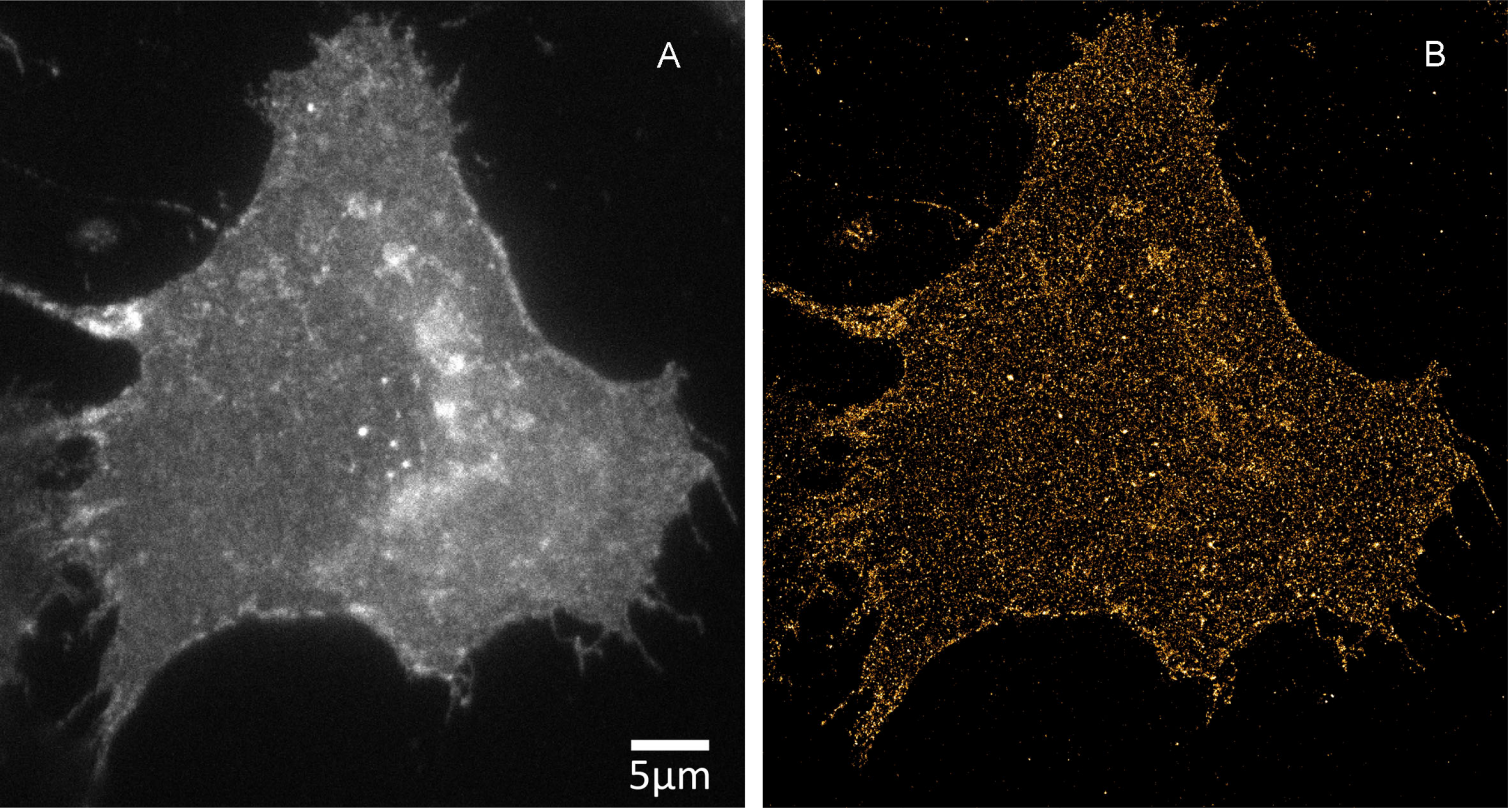
Super-resolution imaging of mCitrine-ErbB3 in A431 cells. (A) Conventional widefield. (B) SMLM reconstruction.

**Figure 5:**
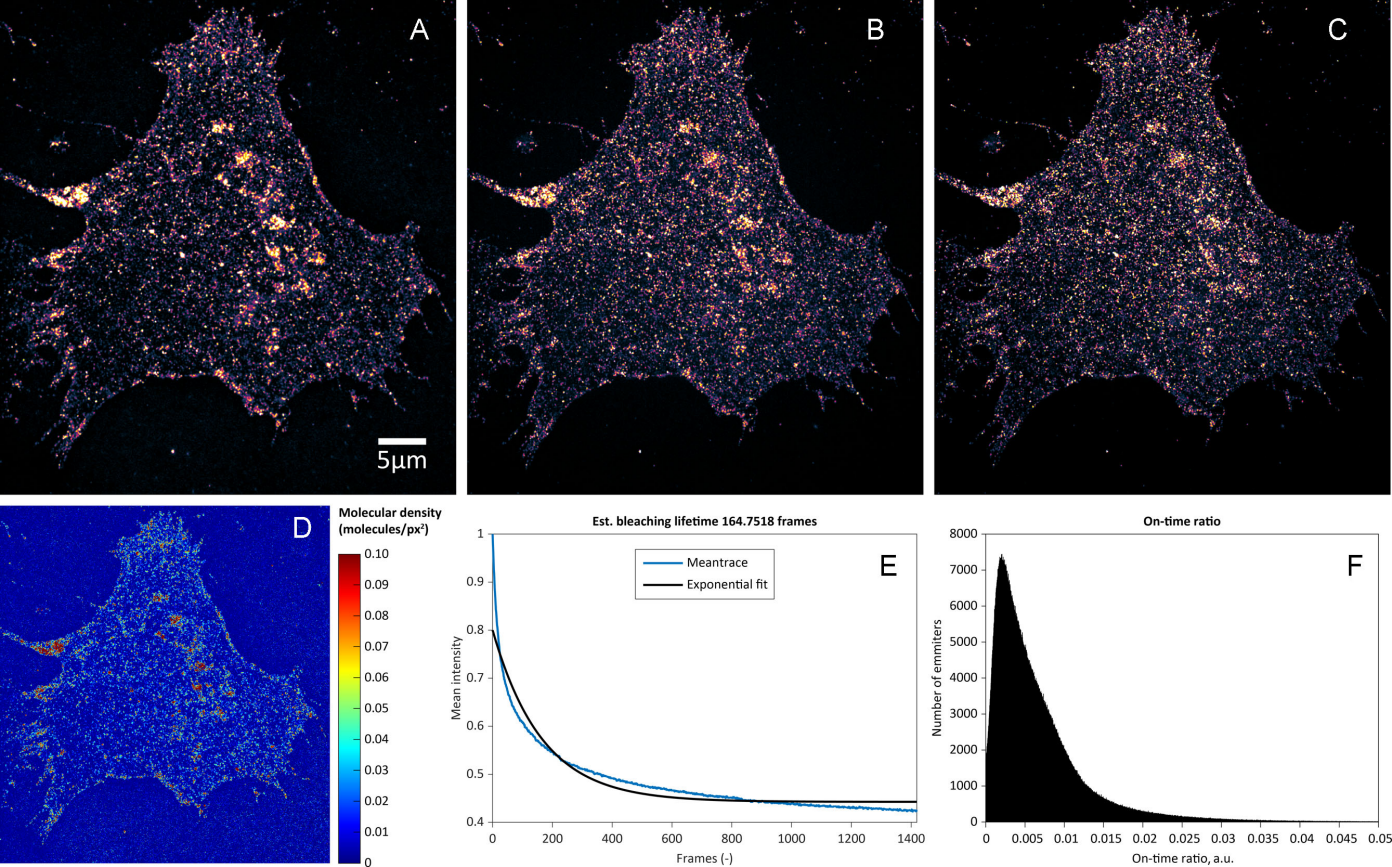
Super-resolution imaging of mCitrine-ErbB3 in A431 cells. (A), (B), and (C) are second-, third-, and fourth-order bSOFI reconstruction, respectively. (D) Molecular density map estimated using bSOFI (E) Mean intensity trace of the raw image sequence (blue) with the exponential fit (black) used for photobleaching correction. (F) Histogram of the on-time ratio estimated using the bSOFI algorithm.

**Figure 6:**
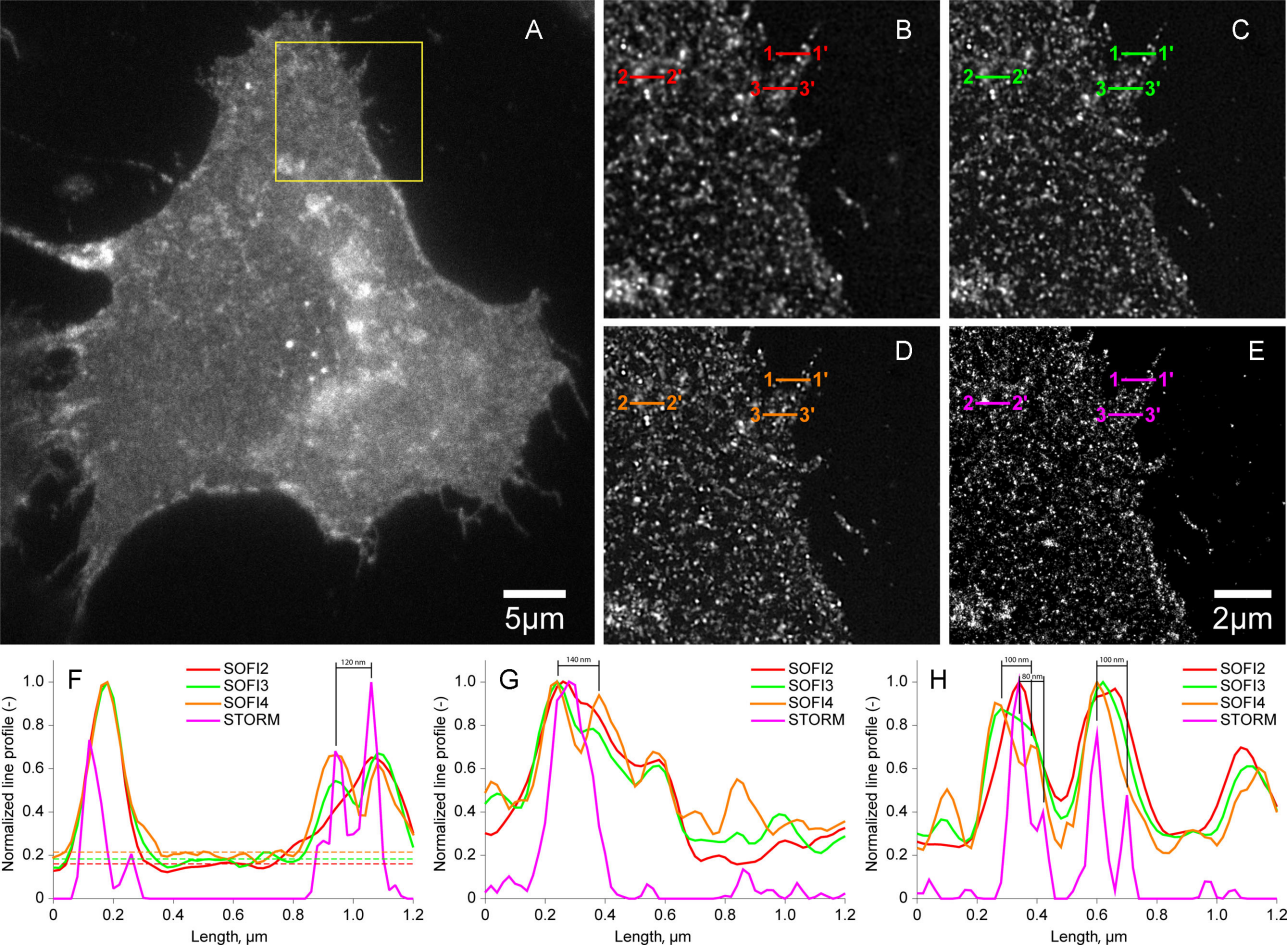
Super-resolution imaging of mCitrine-ErbB3 in A431 cells. (A) Conventional widefield. Region of interest marked in (A) by the yellow square processed by second-order bSOFI (B), third-order bSOFI (C), fourth-order bSOFI (D), SMLM (E). (F–H) Line profiles along the cuts 1–1’, 2–2’, and 3–3’, which correspond to examples of low density of emitters (F), high density (G), and medium density (H), respectively. Dashed lines in (F) represent the average value of the background of bSOFI images.

Figure 4 shows WF imaging (Fig. 4A) and the reconstructed SMLM result (YFP dataset 3) (Fig. 4B) [53]. Figure 5 shows SOFI analysis for the cell shown in Fig. 4. Second-, third-, and fourth-order bSOFI images are shown in Fig. 5A–C, as well as a density map (Fig. 5D), photobleaching profile (Fig. 5E), and molecular on-time ratio (Fig. 5F), where second, third, and fourth denote the order of the cumulant used during the calculation of the bSOFI image. With increasing cumulant order of the SOFI analysis, spatial resolution generally increases, but the signal-to-background ratio (SBR) limits the spatial resolution achievable in practice. The situation is shown in detail in Fig. 6B–D and in the line profiles in Fig. 6F–H. The fourth-order bSOFI image (Fig. 6D) has higher spatial resolution compared with the second- and third-order bSOFI images (Fig. 6B and C). The dashed lines in Fig. 6F show the average value of the background of the bSOFI images, which increases with increasing order of SOFI analysis. In other words, increasing the cumulant order leads to a decrease in SBR, which hampers the resolution enhancement. Note that we calculated linearized SOFI as previously described [49, 50]. In the case of a relatively low density of emitters (Fig. 6F, H), SMLM achieved better spatial resolution. On the other hand, in Fig. 6G, the SMLM analysis does not agree with the result from SOFI, suggesting that the local density of emitters was too high for successful single molecule identification and fitting in that particular location of the cell membrane.

Comparing the density maps in Figs 1D and 5D, the sample in Fig. 5D exhibits an average density approximately 1.8-fold higher. The presence of more emitters in the sample (Fig. 5D) leads to higher brightness, which is likely the reason why the bSOFI image reconstruction was still successful despite the lower number of input frames.

Table 2 shows a summary of the imaging conditions and quantitative parameters for the YFP and Alexa 532 datasets. Also shown are the relevant camera settings. The camera setting information should be entered into ThunderSTORM’s camera setup tab to ensure correct results.

**Table 2:** Summary of imaging conditions and quantitative parameters for the SMLM datasets

Data	Exp. time, ms	Pixel size, nm	e^−^ per A/D count^a^	Base level, A/D counts	EM gain	Frames	Total number of detections	Sigma, nm mean+/−SD	Loc. uncertainty, nm mean+/−SD
YFP data 1 (Fig. 1)	50	80	3.6	414	150	10 000	482 778	86.9+/−29.0	25.6+/−8.6
YFP data 2 (Fig. 2)	100	80	3.6	414	50	6366	224 175	84.1+/−26.3	27.3+/−8.7
YFP data 3 (Figs 4–6)	50	80	3.6	414	150	1419	452 498	84.2+/−24.3	28.9+/−8.1
YFP data 4	100	80	3.6	414	100	3922	159 463	81.6+/−24.1	25.9+/−8.0
Alexa 532 data	30	80	1.5	396	50	20 000	1 128 322	121.5+/−47.6	20.6+/−7.5

^a^Photoelectrons per analog to digital converter count.

## Re-use Potential

Super-resolution microscopy algorithms are under active development [10]. Researchers engaged in algorithm development may use this dataset to help develop and fine-tune their methods. As the true positions of the molecules remain unknown, the results from ThunderSTORM may be taken as the reference data for comparison purposes. ThunderSTORM offers an analysis tool that compares reference data and experimental data and computes several quantities that can be used to quantitatively evaluate algorithm performance. A detailed example of use is provided in the Supplementary Data.

## Availability of source code and requirements

Project name: ThunderSTORM v1.3

Project home page: http://zitmen.github.io/thunderstorm/

Operating system: platform-independent

Programming language: Java

Other requirements: Image J https://imagej.nih.gov/ij/

License: GNU General Public License v3.0

## Availability of data

All raw and analyzed data are available in the *GigaScience* repository, *Giga*DB [53].

## Additional files

Figure S1: Counting localized and missed molecules. Red dot: ground-truth position of a molecule; blue cross: localized molecule; green arrow: association of a localized molecule with ground-truth position; dashed circle: detection tolerance radius. (A) 1 TP + 1 FP; (B) 1 FN + 2 FP; (C, D) example of a situation where (C) greedy approach fails by finding 1 TP + 1 FP + 1 FN and (D) Gale-Shapley algorithm finds a correct solution with 2 TP.

Figure S2: (A) Input image (frame 100 of “YFP dataset 2”), (B) ThunderSTORM setup, default settings with maximum likelihood fitting method selected, (C) ThunderSTORM setup, default settings with weighted least squares fitting method selected.

Figure S3: (A) ThunderSTORM results table using maximum likelihood fitting. (B) ThunderSTORM results table using weighted least squares fitting. The results indicate true-positive detections (green), false-positive detections (red), and false negatives (orange). (C) Table of results when varying the molecule matching tolerance. Statistics are calculated that quantitatively compare the 2 results tables.

## Abbreviations

(d)STORM: (direct) stochastic optical reconstruction microscopy; FWHM: full width at half maximum; GFP: green fluorescent protein; NA: numerical aperture; PALM: photoactivated localization microscopy; PSF: point spread function; SMLM: single molecule localization microscopy; SOFI: stochastic optical fluctuation imaging; WF: wide field; YFP: yellow fluorescent protein.

## Competing interests

The authors declare that they have no competing interests.

## Funding

This work was supported by the UCCS center for the University of Colorado BioFrontiers Institute, by the Czech Science Foundation (GA17-05840S Multicriteria optimization of shift-variant imaging system models), and by Czech Technical University in Prague (grant number SGS16/167/OHK3/2T/13). T.L. acknowledges a SCIEX scholarship (project code 13.183). The funding sources had no involvement in study design; in the collection, analysis, or interpretation of data; in the writing of the report; or in the decision to submit the article for publication.

## Author contributions

T.L.: analyzed data, developed computer code, wrote the paper; J.P.: analyzed data, developed computer code; K.F.: supervised research; T.L.: supervised research; G.H.: conceived project, acquired data, analyzed data, supervised research, wrote the paper.

## Supplementary Material

GIGA-D-17-00208_Original_Submission.pdf

GIGA-D-17-00208_Revision_1.pdf

Response_to_Reviewer_Comments_Original_Submission.pdf

Reviewer_1_Report_(Original_Submission) -- Graham Wright 25 Sep 2017 Reviewed

Reviewer_2_Report_(Original_Submission) -- Sebastian Van de Linde 13 Oct 2017 Reviewed

Supplemental material
