# Supplementary material for: Quantitative super-resolution single molecule microscopy dataset of YFP-tagged growth factor receptors
Source: Gigascience. 2018 Jan 19;7(3):1–10. doi: 10.1093/gigascience/giy002 (PMC5841371; doi:10.1093/gigascience/giy002)
Supplement: GIGA-D-17-00208_Original_Submission.pdf [file giy002_giga-d-17-00208_original_submission.pdf]

GigaScience

Quantitative super-resolution single molecule microscopy dataset of YFP-tagged growth factor receptors

--Manuscript Draft--

**Manuscript Number:**	GIGA-D-17-00208
**Full Title:**	Quantitative super-resolution single molecule microscopy dataset of YFP-tagged growth factor receptors
**Article Type:**	Data Note
**Funding Information:**	BioFrontiers Institute, University of Colorado Colorado Springs — Dr Guy Hagen<br>České Vysoké Učení Technické v Praze (CZ) (SGS16/167/OHK3/2T/13) — Dr Karel Fliegel<br>Grantová Agentura České Republiky — Dr Guy Hagen<br>SCIEX (13.183) — Dr Tomáš Lukeš
**Abstract:**	Background: Super-resolution single molecule localization microscopy (SMLM) is a method for achieving resolution beyond the classical limit in optical microscopes. Yellow fluorescent protein (YFP) has been used for super-resolution single molecule localization microscopy, but more rarely compared to other probes. Working with YFP in SMLM is a challenge because a lower number of photons are emitted per molecule compared to organic dyes which are more commonly used. Publically available experimental data can facilitate development of new data analysis algorithms.<br><br>Findings: Four complete, freely available single molecule super-resolution microscopy datasets on<br>YFP-tagged growth factor receptors expressed in a human cell line are presented, including raw and analyzed data. We report methods for sample preparation, for data acquisition, and for data analysis, and examples of the acquired images. We also analyzed the SMLM data sets using a different method, super-resolution optical fluctuation imaging (SOFI). The two modes of analysis offer complementary information about the sample.<br><br>Conclusion: This dataset has potential for extensive reuse. Complete raw data from SMLM experiments has typically not been published. The data exhibits low signal to noise ratios, making data analysis a challenge. The data sets will be useful to investigators developing their own algorithms for SMLM, SOFI, and related methods. The data will also be useful for researchers investigating growth factor receptors such as erbB3.
**Corresponding Author:**	Guy Hagen<br><br>UNITED STATES
**Corresponding Author Secondary Information:**	
**Corresponding Author's Institution:**	
**Corresponding Author's Secondary Institution:**	
**First Author:**	Tomáš Lukeš
**First Author Secondary Information:**	
**Order of Authors:**	Tomáš Lukeš
	Jakub Pospíšil
	Karel Fliegel

Powered by Editorial Manager® and ProduXion Manager® from Aries Systems Corporation

	Theo Lasser
	Guy Hagen
**Order of Authors Secondary Information:**	
**Opposed Reviewers:**	
**Additional Information:**	
**Question**	**Response**
Are you submitting this manuscript to a special series or article collection?	No
**Experimental design and statistics**<br><br>Full details of the experimental design and statistical methods used should be given in the Methods section, as detailed in our Minimum Standards Reporting Checklist. Information essential to interpreting the data presented should be made available in the figure legends.<br><br>Have you included all the information requested in your manuscript?	Yes
**Resources**<br><br>A description of all resources used, including antibodies, cell lines, animals and software tools, with enough information to allow them to be uniquely identified, should be included in the Methods section. Authors are strongly encouraged to cite Research Resource Identifiers (RRIDs) for antibodies, model organisms and tools, where possible.<br><br>Have you included the information requested as detailed in our Minimum Standards Reporting Checklist?	Yes
**Availability of data and materials**<br><br>All datasets and code on which the conclusions of the paper rely must be either included in your submission or deposited in publicly available repositories (where available and ethically appropriate), referencing such data using a unique identifier in the references and in the "Availability of Data and Materials" section of your manuscript.<br><br>Have you have met the above requirement as detailed in our Minimum	No

Standards Reporting Checklist?	
If not, please give reasons for any omissions below.<br><br>as follow-up to "**Availability of data and materials**<br><br>All datasets and code on which the conclusions of the paper rely must be either included in your submission or deposited in publicly available repositories (where available and ethically appropriate), referencing such data using a unique identifier in the references and in the “Availability of Data and Materials” section of your manuscript.<br><br>Have you have met the above requirement as detailed in our Minimum Standards Reporting Checklist?<br><br>"	we plan to submit the data to GigaDB when the editor assigns the paper to reviewers

Manuscript Click here to download Manuscript Hagen YFP imaging.docx

Title Quantitative super-resolution single molecule microscopy dataset of YFP-tagged growth factor receptors

Authors Tomáš Lukeš[1], Jakub Pospíšil[2], Karel Fliegel[2], Theo Lasser[1], Guy M. Hagen[3]

Affiliations [1]Laboratoire d'Optique Biomédicale, École Polytechnique Fédérale de Lausanne, CH-1015 Lausanne, Switzerland

[2]Department of Radioelectronics, Faculty of Electrical Engineering, Czech Technical University in Prague, Technická 2, 16627 Prague 6, Czech Republic

[3]UCCS center for the Biofrontiers Institute, University of Colorado at Colorado Springs, 1420 Austin Bluffs Parkway, Colorado Springs, Colorado, 80918, USA

Contact email addresses

Tomáš Lukeš lukestom@fel.cvut.cz

Jakub Pospíšil, pospij27@fel.cvut.cz

Zdeněk Švindrych, zs4d@virginia.edu

Karel Fliegel, fliegek@fel.cvut.cz

Theo Lasser, theo.lasser@epfl.ch

Kathrin Spendier, kspendie@uccs.edu

Corresponding author, Guy M. Hagen, ghagen@uccs.edu

Abstract

Background: Super-resolution single molecule localization microscopy (SMLM) is a method for achieving resolution beyond the classical limit in optical microscopes. Yellow fluorescent protein (YFP) has been used for super-resolution single molecule localization microscopy, but more rarely compared to other probes. Working with YFP in SMLM is a challenge because a lower number of photons are emitted per

molecule compared to organic dyes which are more commonly used. Publically available experimental data can facilitate development of new data analysis algorithms.

Findings: Four complete, freely available single molecule super-resolution microscopy datasets on YFP-tagged growth factor receptors expressed in a human cell line are presented, including raw and analyzed data. We report methods for sample preparation, for data acquisition, and for data analysis, and examples of the acquired images. We also analyzed the SMLM data sets using a different method, super-resolution optical fluctuation imaging (SOFI). The two modes of analysis offer complementary information about the sample.

Conclusion: This dataset has potential for extensive reuse. Complete raw data from SMLM experiments has typically not been published. The data exhibits low signal to noise ratios, making data analysis a challenge. The data sets will be useful to investigators developing their own algorithms for SMLM, SOFI, and related methods. The data will also be useful for researchers investigating growth factor receptors such as erbB3.

Keywords: super-resolution microscopy, PALM, STORM, SOFI, YFP, SMLM, single molecule, growth factor receptor, erbB3, ThunderSTORM

Data description

Context

Fluorescence optical microscopy is one of the most important tools available for the study of biological systems at the cellular level. Unfortunately, due to diffraction phenomena the resolution of fluorescence microscopes in the lateral dimension is limited to $0.61\lambda/NA$, where λ is the wavelength of the detected light, and NA is the numerical aperture of the objective lens. As many biological structures within cells are much smaller than this, increasing resolution is of prime importance. Today several methods have been developed which are able to image below the diffraction limit [1,2].

Photoactivated localization microscopy (PALM) [3] was initially accomplished with the photoconvertible fluorescent protein mEOS [4]. A similar method, (direct) stochastic optical reconstruction

microscopy (d)STORM utilizes organic dyes [5–8]. In these super-resolution methods, single fluorescent molecules are induced to blink on and off (photoswitching) randomly in the sample. A sensitive camera is used to record an image sequence of the single molecule blinking events, and a computational algorithm is used to fit the imaged point spread functions (PSFs) to a model function [9,10]. By doing so, the coordinates of each molecule can be determined with an accuracy much better than the diffraction limit [11]. Once enough molecules have been imaged (usually 10^6-10^7 are required, depending on the sample structure [12]), an image can be reconstructed with lateral resolution improved by about a factor of 10. Together, this family of methods is known as single molecule localization microscopy (SMLM).

Although PALM experiments were initially performed with fluorescent proteins which are specifically photoconvertible [3], green fluorescent protein (GFP) and its spectral variant yellow fluorescent protein (YFP) are also known to exhibit blinking characteristics [13]. GFP and YFP have been used in SMLM, but more rarely [14–20]. Here we used a modified YFP known as mCitrine [21] for SMLM of the growth factor ErbB3 in A431 epithelial carcinoma cells. The organization and dynamics of erbB receptors is an important topic of study because overexpression and unrestrained activation of this family of receptors is implicated in many types of cancer [22].

This dataset has potential for extensive reuse. Complete raw data from SMLM experiments has typically not been published. The data exhibits low signal to noise ratios, making data analysis a challenge. The data sets will be useful to investigators developing their own algorithms for SMLM, SOFI, and related methods. The data will also be useful for researchers investigating growth factor receptors such as erbB3, as well as to those investigating other membrane proteins.

Methods

Cell lines and reagents

A431 cells expressing mCitrine-erbB3 [23] were maintained in phenol red-free DMEM supplemented with 10 % FCS, 100 U/ml penicillin, 100 U/ml streptomycin, and L-glutamate (obtained from Invitrogen, Carlsbad, CA, USA) at 37 °C and 100% humidity. Mowiol 4-88 containing 1,4-diazabicyclo(2.2.2)octane

(DABCO) was obtained from Fluka (St. Louis, MO, USA). Mercaptoethylamine (MEA) was obtained from Sigma (St. Louis, MO, USA).

Sample preparation

Prior to SMLM experiments, the cells were grown on clean #1.5 coverslips for 12-18 hours. The cells were then washed with PBS, then fixed with 4% paraformaldehyde for 15 minutes at 4 °C. We then mounted the cells on clean slides using freshly prepared mowiol containing DABCO and 50-100 mM MEA. Before microscopy, the mowiol was allowed to harden for 12-18 hours.

Single molecule microscopy

For SMLM imaging, we used an IX71 microscope equipped with a planapochromatic 100×/1.35 NA oil immersion objective (Olympus, Tokyo, Japan) and a front-illuminated Ixon DU885 EMCCD camera under control of IQ software (Andor, Belfast, Northern Ireland) as previously described[24]. The excitation source was a 400 mW, 473 nm laser (Dragon laser, ChangChun, China), which was coupled to the microscope using a 0.39 NA multimode optical fiber. The fiber output was collimated using a 2 inch diameter, 60 mm FL lens (Thor Labs, Newton, New Jersey). The fiber was coupled into the microscope using an Olympus IX2-RFAL fluorescence illuminator, resulting in an evenly illuminated field. mCitrine-erbB3 fluorescence was observed using an Olympus U-MNIBA3 filter set (excitation 470 – 495 nm, dichroic 505 nm, emission 510 – 550 nm). In each experiment, a sequence of 1,419-10,000 images was acquired with an exposure time of 40 – 100 ms and an EM gain of 50-300.

Data analysis methods

We analyzed the data using ThunderSTORM [9,25] with the default settings. The default settings involve use of a wavelet-based filter for feature enhancement [26], followed by local maximum detection of single molecules in the filtered data. This is followed by fitting molecules in the raw data using a two-dimensional Gaussian function in integrated form [27] using maximum likelihood methods [28]. Gaussian functions have been found to be a good representation of the true PSF of a microscope [29]. For visualization of the results, we use an average shifted histogram approach [30]. If the camera calibration parameters (pixel size, photoelectrons per A/D count, base level, and EM gain) are correct, maximum likelihood fitting of an

integrated Gaussian function will correctly return the number of photons detected from each molecule [9,27,28,31]. An integrated two dimensional Gaussian function can be written as

$$\begin{aligned} &PSF_{IG}(x, y \mid \theta) = \theta_N E_x E_y + \theta_b, \\ &E_x = \frac{1}{2}\operatorname{erf}\left(\frac{x-\theta_x + \frac{1}{2}}{\sqrt{2}\theta_\sigma}\right) - \frac{1}{2}\operatorname{erf}\left(\frac{x-\theta_x - \frac{1}{2}}{\sqrt{2}\theta_\sigma}\right), \\ &E_y = \frac{1}{2}\operatorname{erf}\left(\frac{y-\theta_y + \frac{1}{2}}{\sqrt{2}\theta_\sigma}\right) - \frac{1}{2}\operatorname{erf}\left(\frac{y-\theta_y - \frac{1}{2}}{\sqrt{2}\theta_\sigma}\right), \end{aligned} \tag{1}$$

where θ_x, θ_y are the sub-pixel molecular coordinates, θ_σ is the standard deviation of the Gaussian function (i.e., the width), θ_N is the total number of detected photons emitted by the molecule, and θ_b is the background offset.

Single molecule localization uncertainty

In ThunderSTORM the localization uncertainty is calculated for each detected molecule. This quantity can help one determine whether the molecule was well localized and whether it should be included in the final result. Let $\hat{\theta}_\sigma$ be the standard deviation of a Gaussian function fitted to an imaged PSF in nm, a is the backprojected pixel size in nm (camera pixel size divided by system magnification), $\hat{\theta}_N$ is the estimate of the number of photons detected for a given molecule, and $\hat{b}$ is the background signal level in photons calculated as the standard deviation of the residuals between the raw data and the fitted PSF model. The uncertainty of estimates determined by maximum likelihood methods for the lateral position of a molecule is given by

$$\left(\Delta\hat{\theta}_{xy}\right)^2 = \frac{g\hat{\theta}_{\sigma^2} + a^2/12}{\hat{\theta}_N}\left(1 + 4\tau + \sqrt{\frac{2\tau}{1+4\tau}}\right), \tau = \frac{2\pi\left(\hat{b}^2 + r\right)\left(\hat{\theta}_\sigma^2 + a^2/12\right)}{a^2\hat{\theta}_N}. \tag{2}$$

This formula is a modified form of the Thompson-Larson-Webb equation [11], and was derived by Rieger and Stallinga [32]. Finally, compensation for camera readout noise r and EM gain g was added following Quan, Zeng, and Huang [33], who suggested that when using EMCCD cameras, the correction factors should be set to $r = 0$, $g = 2$, and when using CCD or sCMOS cameras the correction factors should be set to $r = g = 2$.

Super-resolution optical fluctuation imaging

Super-resolution optical fluctuation imaging (SOFI) is based on spatio-temporal cumulants calculated over the input sequence of camera frames [34]. Assuming a non-fluctuating background and Gaussian additive noise, the n-th order cumulant (for $n \geq 2$ and a time lag τ) can be written as

$$\kappa_n\{I(\mathbf{r},t)\}(\tau) = \sum_{k=1}^{N} \varepsilon_k^n U^n(\mathbf{r}-\mathbf{r}_k)\kappa_n\{s_k(t)\}(\tau), \tag{3}$$

where $I(\mathbf{r},t)$ is the detected intensity at position $\mathbf{r}$ and time t, ε_k is the molecular brightness of k-the emitter, $U^n(\mathbf{r}-\mathbf{r}_k)$ is the PSF at the position $\mathbf{r}_k$, and $s_k(t)$ denotes a normalized fluctuation sequence $s_k(t) \in \{0,1\}$. The PSF is raised to the n-th power, resulting in resolution increased by a factor of $\sqrt{n}$. After reweighting in frequency space, a resolution enhancement factor of *n* can be achieved [35], scaling linearly with the cumulant order. SOFI can be applied to any image sequence of stochastically blinking emitters acquired from a conventional widefield microscope if the emitters switch between at least two optically distinguishable states (a dark state and a bright state) and if sampling of the PSF fulfills the Nyquist–Shannon sampling theorem [36]. In comparison to STORM, SOFI tolerates higher densities of emitters and/or higher blinking rates [37], resulting in improved temporal resolution [38]. SOFI can be applied on the same datasets as STORM/PALM [37,39] offering an interesting complement to SMLM methods. Due to the entirely different image processing methods used, SOFI and SMLM are prone to different artifacts. Applying both processing methods to the same dataset reveals more information about the true structure and properties of the underlying sample. By combining multiple orders of the SOFI analysis, molecular parameters like molecular density, brightness, and on-time ratio can be extracted using the balanced SOFI method (bSOFI) [40]. The on-time ratio ρ_{on} describes the blinking rate of the fluorescent label. Assuming a two state blinking model where the emitter fluctuates between a bright state and a dark state, the on-time ratio is given as [38]

$$\rho_{on} = \frac{\tau_{on}}{\tau_{on} + \tau_{off}}, \tag{4}$$

where the τ_{on} and τ_{off} are the characteristic lifetimes of the bright state and the dark state, respectively.

SOFI analysis was carried out as reported previously [39]. We used a custom written algorithm (Matlab, The Mathworks) based on the code of our SOFI simulation tool [41] and the bSOFI algorithm [40]. The sequence of camera frames was divided into subsequences of 500 frames each. The subsequences were processed separately in order to minimize the influence of photobleaching and the resulting SOFI images were averaged. More details about photobleaching correction for SOFI can be found in [42].

Super-resolution images

Figure 1 shows images of an A431 cell expressing mCitrine-erbB3 (YFP dataset 1, DOI to be provided). Conventional widefield (WF, Fig. 1A), and SMLM (Fig. 1B) results are shown. Fig. 1C shows a color-coded density map, calculated by the bSOFI algorithm. This unique information cannot be obtained by conventional fluorescence microscopy. Fig. 4D shows the results of 4th order SOFI analysis.

INSERT FIGURE 1

Figure 2A shows a histogram of the number of photons detected from each YFP molecule (“intensity” in ThunderSTORM) for the cell shown in Fig 1. Fig. 2B shows a histogram of the localization uncertainty determined for each molecule for the cell shown in Fig 1. The localization uncertainty was calculated using Eq. 2. The two histograms were calculated using the *plot histogram* command in ThunderSTORM.

INSERT FIGURE 2

Table 1 shows a list of quantitative parameters for the first 10 detected molecules as reported by ThunderSTORM for the experiment shown in Figure 1. Sigma (nm) is the fitted standard deviation of the two-dimensional integrated Gaussian function fitted to the molecule, intensity (photons) is the number of photons detected from the molecule, offset (photons) is the background offset, SD of background (photons) is the standard deviation of the background, and localization uncertainty (nm) is the result of Equation 2 for each molecule. Recall that the full width at half max (FWHM) of a Gaussian function is related to its standard deviation by FWHM=2.35σ.

Table 1 Quantitative parameters for the first 10 detected molecules as reported by ThunderSTORM for the experiment shown in Fig. 1.

Molecule number	Camera frame	x (nm)	y (nm)	sigma (nm)	intensity (photons)	offset (photons)	SD of Background (photons)	localization uncertainty (nm)
1	1	3743.17	28005.63	81.53	942	108	25	17.41
2	1	3880.95	31519.89	155.09	2014	68	21	23.33
3	1	4150.78	32662.21	60.03	433	81	21	17.75
4	1	4289.06	28407.32	36.90	407	155	32	12.97
5	1	4310.28	28737.99	103.00	1567	142	34	21.61
6	1	4615.06	23832.74	89.18	1186	73	22	14.60
7	1	4695.34	30060.77	102.05	1266	122	28	22.17
8	1	4812.40	30994.57	101.18	1051	115	24	22.61
9	1	4827.01	25960.59	83.02	717	80	20	19.35
10	1	5037.67	28686.08	149.32	2293	121	33	29.85

Figure 3 shows WF imaging of an A431 cell (Fig. 3A), along with identification of single molecules by ThunderSTORM (Fig. 3B, indicated by red dots), and the reconstructed SMLM result (Fig. 3C) (YFP dataset 2, DOI to be provided).

INSERT FIGURE 3

Figure 4 shows WF imaging (Fig. 4A), and the reconstructed SMLM result (Fig. 4B) (YFP dataset 3, DOI to be provided). Figure 5 shows SOFI analysis for the cell shown in Figure 4. Second, third, and fourth order SOFI are shown (Fig. 5A-5C), as well as a density map (Fig 5D), photobleaching profile (Fig. 5E), and molecular on-time ratio (Fig 5F).

INSERT FIGURE 4

INSERT FIGURE 5

Table 2 shows a summary of the imaging conditions and quantitative parameters for the YFP datasets. Table 3 shows the camera parameters used to acquire the data, this information should be entered into ThunderSTORM's camera setup tab to ensure correct results.

Table 2 Summary of imaging conditions and quantitative parameters for the YFP datasets.

Data	Exposure time, ms	EM Gain	Frames	Total number of detections	Sigma, nm mean+/-SD	Loc. accuracy, nm mean+/-SD
YFP data 1 (Fig.1)	50	150	10000	482,778	86.9+/-29	25.58+/-8.58
YFP data 2 (Fig . 2)	100	50	6366	224,175	84.1+/-26.3	27.3+/-8.7
YFP data 3 (Figs. 4, 5)	50	150	1419	452,498	84.2+/-24.3	28.9+/-8.1
YFP data 4	100	100	3922	159,463	81.6+/-24.1	25.9+/-8.0

Table 3 Camera setup parameters for the YFP datasets

Camera parameter	Value
Pixel size	80 nm
Photoelectrons per A/D count	3.6
Base level A/D counts	414
EM gain	Yes, Refer to Table 2

Re-use potential

Super-resolution microscopy algorithms are under active development. Researchers engaged in algorithm development may use this dataset to help develop and fine tune their methods. Since the true positions of the molecules remain unknown, the results from ThunderSTORM may be taken as the ground truth for evaluation purposes. ThunderSTORM offers an evaluator, which compares ground truth and analyzed data, and computes several quantitates which can be used to evaluate algorithm performance.

Availability of source code and requirements

Project name: ThunderSTORM v1.3

Project home page: http://zitmen.github.io/thunderstorm/

Operating system: platform independent

Programming language: Java

Other requirements: Image J https://imagej.nih.gov/ij/

License: GNU General Public License v3.0

Availability of data

All raw and analyzed data is available on GigaDB at http://gigadb.org/site/index.

Abbreviations

(d)STORM, (direct) stochastic optical reconstruction microscopy; FWHM, full width at half maximum; GFP, green fluorescent protein, NA, numerical aperture; PALM, photoactivated localization microscopy; PSF, point spread function; SMLM, single molecule localization microscopy; SOFI, stochasitic optical fluctuation imaging; WF, wide field; YFP, yellow fluorescent protein.

Ethics approval and consent to participate

Not applicable

Consent for publication

Not applicable

Competing interests

The authors declare that they have no competing interests.

Funding

This work was supported by the UCCS center for the University of Colorado BioFrontiers Institute, by the Czech Science Foundation, and by Czech Technical University in Prague (grant number SGS16/167/OHK3/2T/13). T.L. acknowledges a SCIEX scholarship (project code 13.183). The funding sources had no involvement in study design; in the collection, analysis and interpretation of data; in the writing of the report; or in the decision to submit the article for publication.

Author Contributions

TL: analyzed data, developed computer code, wrote the paper

JP: analyzed data, developed computer code

KF: supervised research

TL: supervised research

GH: conceived project, acquired data, analyzed data, supervised research, wrote the paper

Acknowledgements

Epithelial carcinoma A431 cells expressing mCitrine-erbB3 [23] were a kind gift from Dr. Donna Arndt-Jovin and Dr. Tom Jovin of the Max Planck Institute for Biophysical Chemistry (Göttingen, Germany). We thank Peter W. Winter for assistance with microscopy, and Pavel Křížek, Josef Borkovec, Zdeněk Švindrych, and Martin Ovesný for assistance with microscopy, data analysis, and programming.

References

[1] Huang B, Bates M, Zhuang X. Super-resolution fluorescence microscopy. Annu Rev Biochem 2009;78:993–1016.

[2] Hell SW, Sahl SJ, Bates M, Zhuang X, Heintzmann R, Booth MJ, et al. The 2015 super-resolution microscopy roadmap. J Phys D Appl Phys 2015;48:443001.

[3] Betzig E, Patterson GH, Sougrat R, Lindwasser OW, Olenych S, Bonifacino JS, et al. Imaging intracellular fluorescent proteins at nanometer resolution. Science 2006;313:1642–5.

[4] Wiedenmann J, Ivanchenko S, Oswald F, Schmitt F, Röcker C, Salih A, et al. EosFP, a fluorescent marker protein with UV-inducible green-to-red fluorescence conversion. Proc Natl Acad Sci U S A 2004;101:15905–10.

[5] Rust MJ, Bates M, Zhuang X. Sub-diffraction-limit imaging by stochastic optical reconstruction microscopy (STORM). Nat Methods 2006;3:793–5.

[6] Huang B, Wang W, Bates M, Zhuang X. Three-dimensional super-resolution imaging by stochastic optical reconstruction microscopy. Science 2008;319:810–3.

[7] Heilemann M, van de Linde S, Schüttpelz M, Kasper R, Seefeldt B, Mukherjee A, et al. Subdiffraction-resolution fluorescence imaging with conventional fluorescent probes. Angew Chemie Int Ed 2008;47:6172–6.

[8] Dempsey GT, Vaughan JC, Chen KH, Bates M, Zhuang X. Evaluation of fluorophores for optimal performance in localization-based super-resolution imaging. Nat Methods 2011;8:1–14.

[9] Ovesný M, Křížek P, Borkovec J, Švindrych Z, Hagen GM. ThunderSTORM: A comprehensive ImageJ plug-in for PALM and STORM data analysis and super-resolution imaging. Bioinformatics 2014;30.

[10] Sage D, Kirshner H, Pengo T, Stuurman N, Min J, Manley S, et al. Quantitative evaluation of software packages for single-molecule localization microscopy. Nat Methods 2015;12:717–24.

[11] Thompson RE, Larson DR, Webb WW. Precise nanometer localization analysis for individual fluorescent probes. Biophys J 2002;82:2775–83.

[12] Fox-Roberts P, Marsh R, Pfisterer K, Jayo A, Parsons M, Cox S. Local dimensionality determines imaging speed in localization microscopy. Nat Commun 2017;8:13558.

[13] Dickson RM, Cubitt AB, Tsien RY, Moerner WE. On/off blinking and switching behaviour of single molecules of green fluorescent protein. Nature 1997;388:355–8.

[14] Lemmer P, Gunkel M, Baddeley D, Kaufmann R, Urich A, Weiland Y, et al. SPDM: light microscopy with single-molecule resolution at the nanoscale. Appl Phys B Lasers Opt 2008;93:1–12.

[15] Lemmer P, Gunkel M, Weiland Y, Muller P, Baddeley D, Kaufmann R, et al. Using conventional fluorescent markers for far-field fluorescence localization nanoscopy allows resolution in the 10-nm range. J Microsc 2009;235:163–71.

[16] Biteen JS, Thompson MA, Tselentis NK, Bowman GR, Shapiro L, Moerner WE. Super-resolution imaging in live Caulobacter crescentus cells using photoswitchable EYFP. Nat Methods 2008;5:947–9.

[17] Lew MD, Lee SF, Ptacin JL, Lee MK, Twieg RJ, Shapiro L, et al. Three-dimensional superresolution colocalization of intracellular protein superstructures and the cell surface in live Caulobacter crescentus. Proc Natl Acad Sci 2011;108:E1102–10.

[18] Jusuk I, Vietz C, Raab M, Dammeyer T, Tinnefeld P. Super-Resolution Imaging Conditions for enhanced Yellow Fluorescent Protein (eYFP) Demonstrated on DNA Origami Nanorulers. Sci Rep 2015;5:14075.

[19] Křížek P, Raška I, Hagen GM. Minimizing detection errors in single molecule localization microscopy. Opt Express 2011;19:3226–35.

[20] Kaufmann R, Piontek J, Grüll F, Kirchgessner M, Rossa J, Wolburg H, et al. Visualization and quantitative analysis of reconstituted tight junctions using localization microscopy. PLoS One 2012;7:e31128.

[21] Griesbeck O, Baird GS, Campbell RE, Zacharias DA, Tsien RY. Reducing the Environmental Sensitivity of Yellow Fluorescent Protein. J Biol Chem 2001;276:29188–94.

[22] Yarden Y, Sliwkowski. Untangling the ErbB signaling network. Nat Rev Mol Cell Biol 2001;2:127–37.

[23] Nagy P, Arndt-Jovin DJ, Jovin TM. Small interfering RNAs suppress the expression of endogenous and GFP-fused epidermal growth factor receptor (erbB1) and induce apoptosis in erbB1-overexpressing cells. Exp Cell Res 2003;285:39–49.

[24] Křžek P, Raška I, Hagen GMGMGM, Křížek P, Raška I, Hagen GMGMGM. Flexible structured illumination microscope with a programmable illumination array. Opt Express 2012;20:24585–99.

[25] Ovesný M, Křížek P, Borkovec J, Švindrych Z, Hagen GM. Image Analysis for Single- Molecule Localization Microscopy. In: Diaspro A, Marc A. M. J. van Zandvoort, editors. Super-Resolution Imaging Biomed., Boca Raton, Florida: CRC Press; 2016, p. 79–97.

[26] Izeddin I, Boulanger J, Racine V, Specht CG, Kechkar A, Nair D, et al. Wavelet analysis for single molecule localization microscopy. Opt Express 2012;20:2081–95.

[27] Huang F, Schwartz SL, Byars JM, Lidke KA. Simultaneous multiple-emitter fitting for single molecule super-resolution imaging. Biomed Opt Express 2011;2:1377–93.

[28] Mortensen KI, Churchman LS, Spudich JA, Flyvbjerg H. Optimized localization analysis for single-molecule tracking and super-resolution microscopy. Nat Methods 2010;7:377–81.

[29] Stallinga S, Rieger B. Accuracy of the gaussian point spread function model in 2D localization microscopy. Opt Express 2010;18:24461–76.

[30] Scott DW. Averaged shifted histograms: effective nonparametric density estimators in several dimensions. Ann Stat 1985;13:1024–40.

[31] Smith CS, Joseph N, Rieger B, Lidke KA. Fast, single-molecule localization that achieves theoretically minimum uncertainty. Nat Methods 2010;7:373–5.

[32] Rieger B, Stallinga S. The lateral and axial localization uncertainty in super-resolution light microscopy. Chemphyschem 2014;15:664–70.

[33] Quan T, Zeng S, Huang Z-L. Localization capability and limitation of electron-multiplying charge-coupled, scientific complementary metal-oxide semiconductor, and charge-coupled devices for superresolution imaging. J Biomed Opt 2010;15:066005.

[34] Dertinger T, Colyer R, Iyer G, Weiss S, Enderlein J. Fast, background-free, 3D super-resolution optical fluctuation imaging (SOFI). Proc Natl Acad Sci U S A 2009;106:22287–92.

[35] Dertinger T, Colyer R, Vogel R, Enderlein J, Weiss S. Achieving increased resolution and more pixels with Superresolution Optical Fluctuation Imaging (SOFI). Opt Express 2010;18:18875–85.

[36] Heintzmann R. Band-Limit and appropriate sampling in microscopy . In: Celis Julio E, editor. Cell Biol. A Lab. Handb., Elsevier Academic Press; 2006, p. 29–36.

[37] Geissbuehler S, Dellagiacoma C, Lasser T. Comparison between SOFI and STORM. Biomed Opt Express 2011;2:408–20.

[38] Geissbuehler S, Sharipov A, Godinat A, Bocchio NL, Sandoz P a., Huss A, et al. Live-cell multiplane three-dimensional super-resolution optical fluctuation imaging. Nat Commun 2014;5.

[39] Deschout H, Lukes T, Sharipov A, Szlag D, Feletti L, Vandenberg W, et al. Complementarity of PALM and SOFI for super-resolution live-cell imaging of focal adhesions. Nat Commun 2016;7:13693.

[40] Geissbuehler S, Bocchio NL, Dellagiacoma C, Berclaz C, Leutenegger M, Lasser T. Mapping molecular statistics with balanced super-resolution optical fluctuation imaging (bSOFI). Opt Nanoscopy 2012;1.

[41] Girsault A, Lukes T, Sharipov A, Geissbuehler S, Leutenegger M, Vandenberg W, et al. SOFI Simulation Tool: A Software Package for Simulating and Testing Super-Resolution Optical Fluctuation Imaging. PLoS One 2016;11:e0161602.

[42] Peeters Y, Vandenberg W, Duwé S, Bouwens A, Lukes T, Ruckebusch C, et al. Correcting for photodestruction in super-resolution optical fluctuation imaging. Sci Rep 2017;Accepted:July 2017.

FIGURE CAPTIONS

Fig. 1 Super-resolution imaging of mCitrine-erbB3 in A431 cells. (A) conventional widefield. (B) SMLM. (C) Molecular density map. (D) 4th order bSOFI.

Fig. 2 Quantification of molecular parameters from the experiment shown in Fig. 1. (A) Histogram of the number of photons detected from each YFP molecule. (B) Histogram of the localization uncertainty calculated for each YFP molecule.

Fig. 3 Super-resolution imaging of mCitrine-erbB3 in A431 cells. (A) conventional widefield. (B) single frame of SMLM with detections indicated with red dots. (C) SMLM reconstruction.

Fig. 4 Super-resolution imaging of mCitrine-erbB3 in A431 cells. (A) conventional widefield. (B) SMLM reconstruction.

Fig 5 Super-resolution imaging of mCitrine-erbB3 in A431 cells. (A,) (B) and (C) are second, third and fourth order SOFI reconstruction, respectively. (D) Molecular density map estimated using bSOFI (E) Mean intensity trace of the raw image sequence (blue) with the exponential fit (black) used for photobleaching correction. (F) Histogram of the on-time ratio estimated using bSOFI algorithm.

Figure

Click here to download Figure Fig_1.png

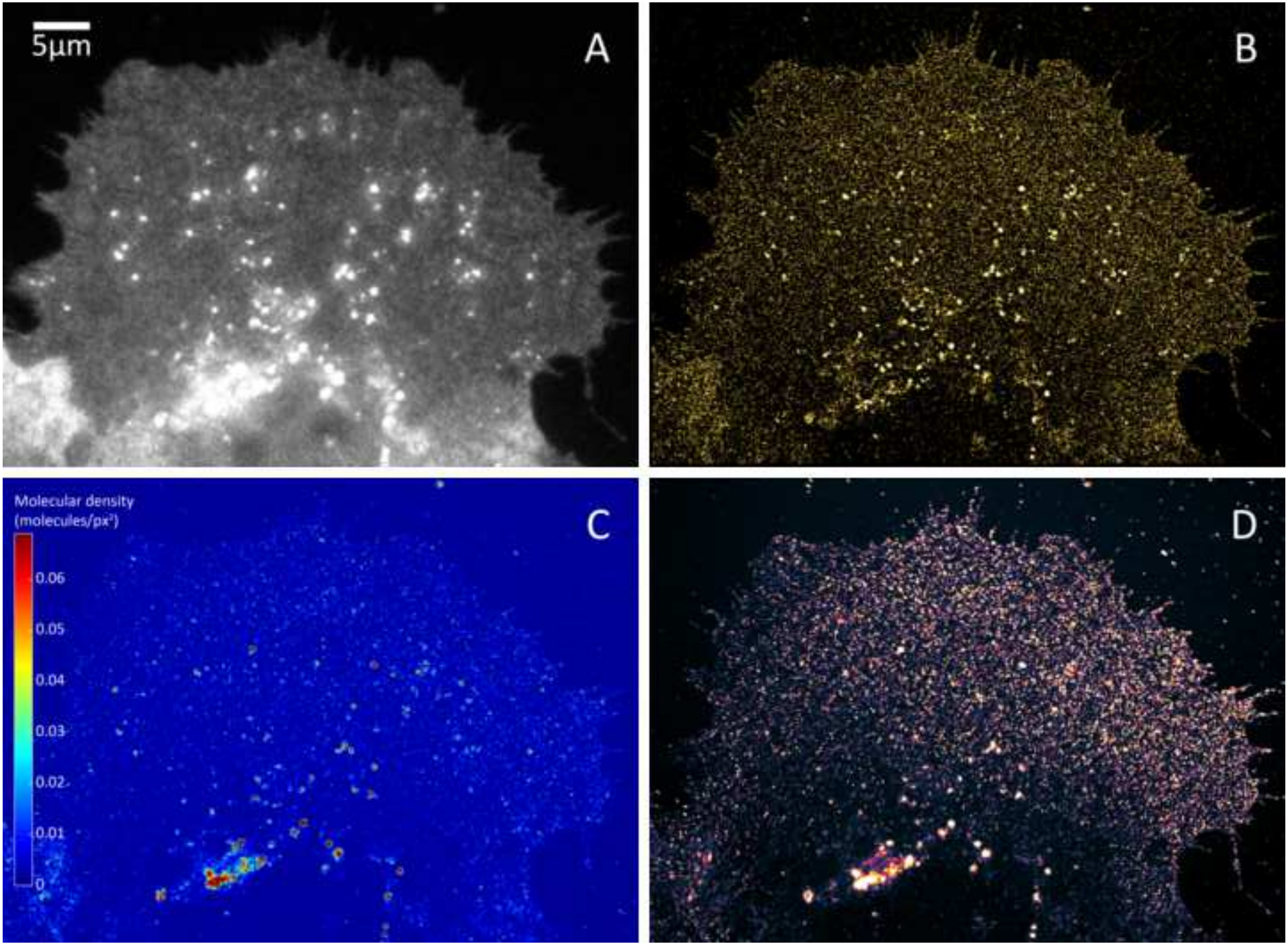

 Click here to download Figure Fig_2.png

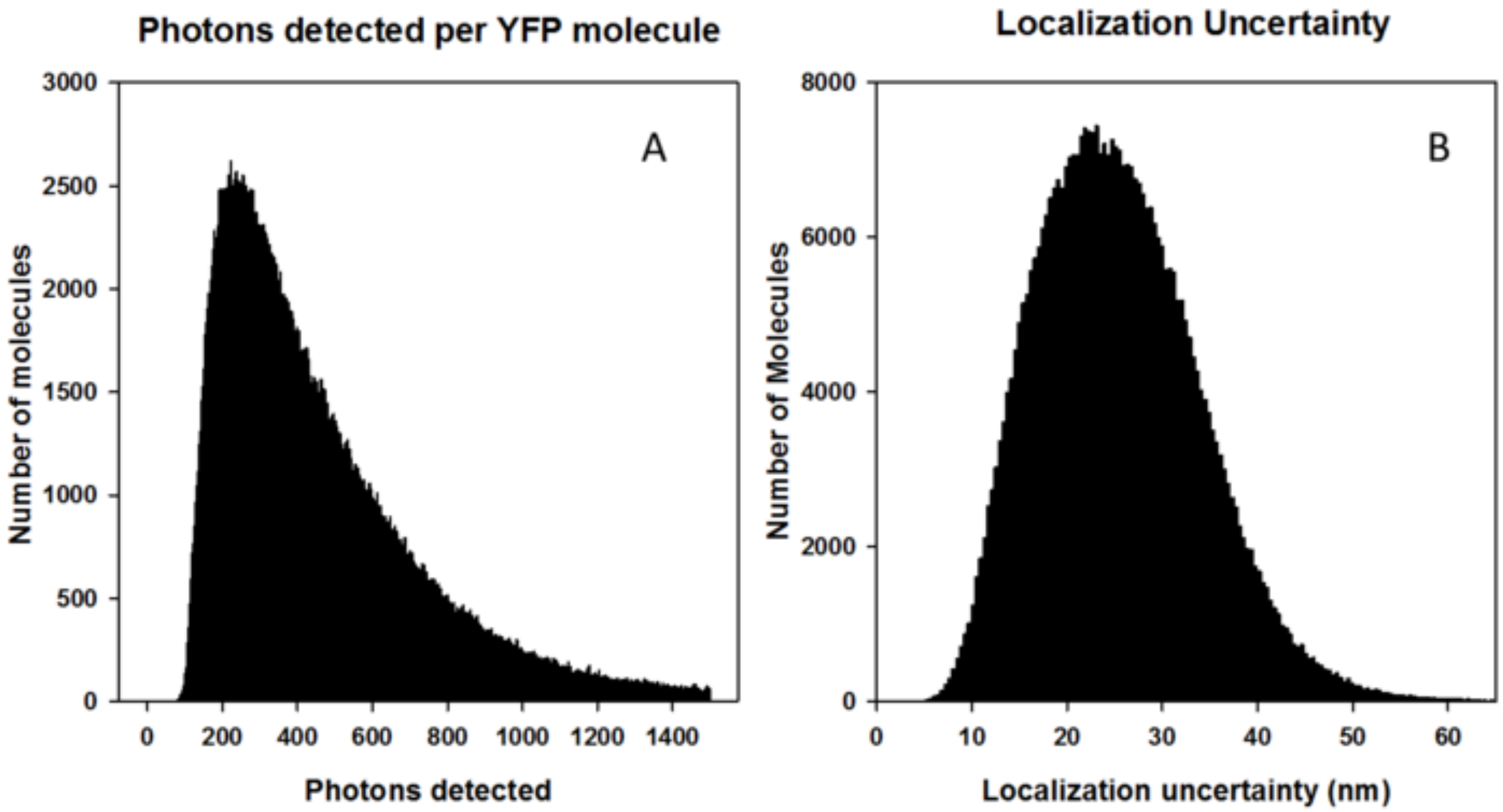

Figure

Click here to download Figure Fig_3.png

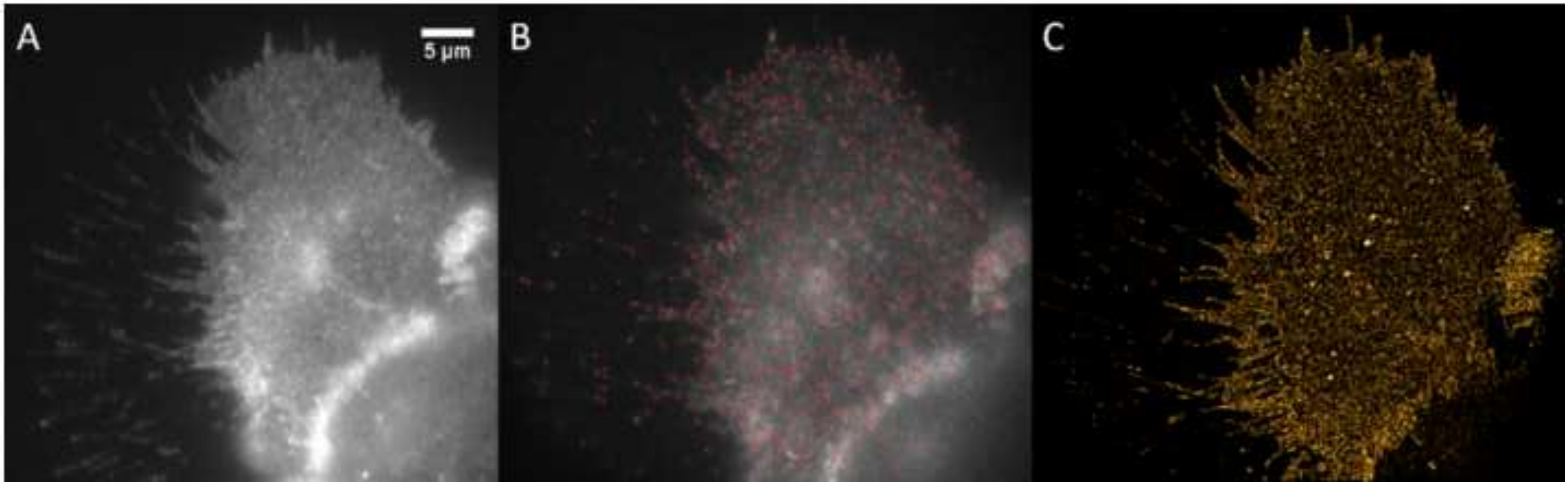

 Click here to download Figure Fig_4.png

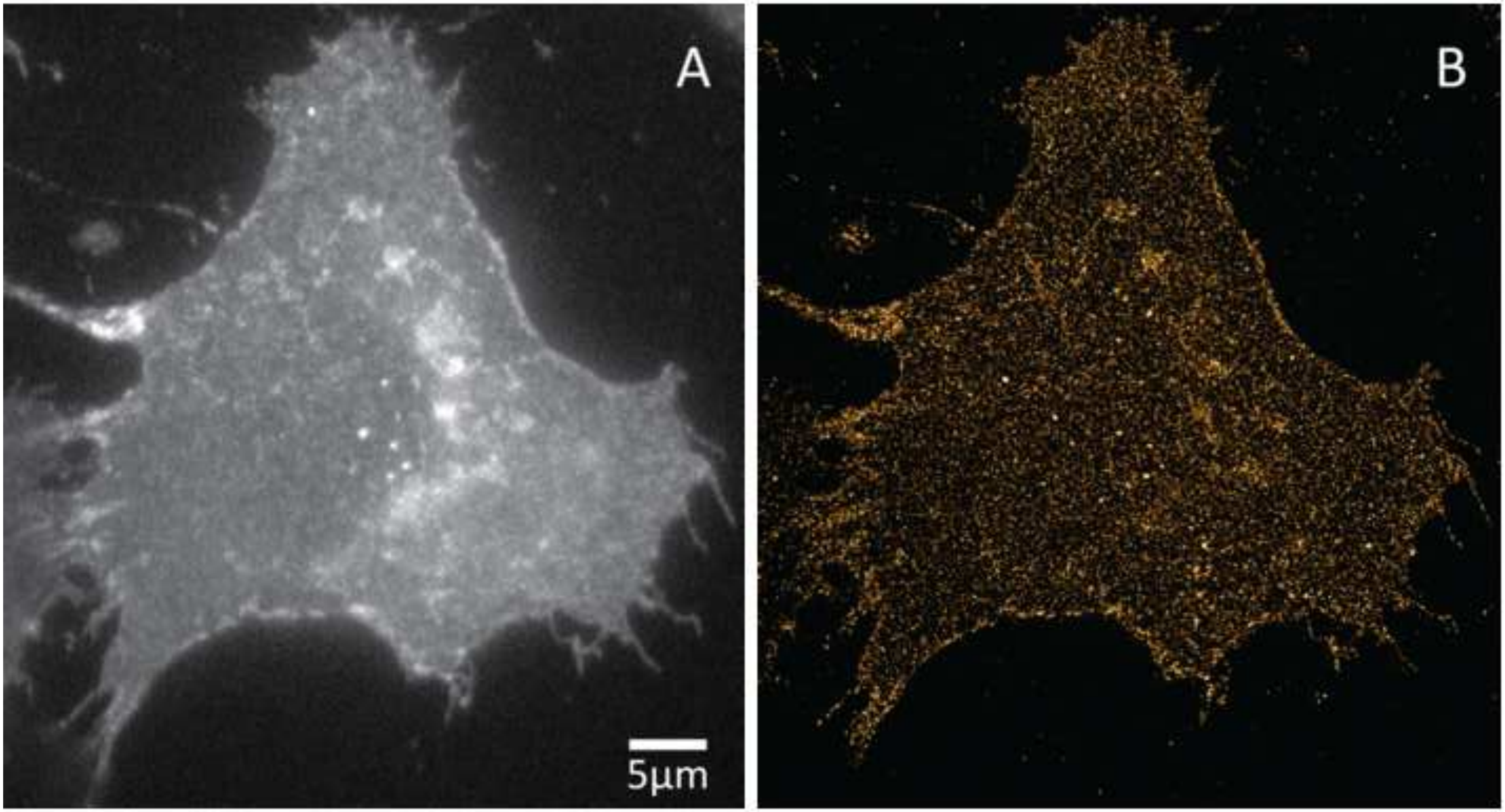

Figure Click here to download Figure Fig_5.png

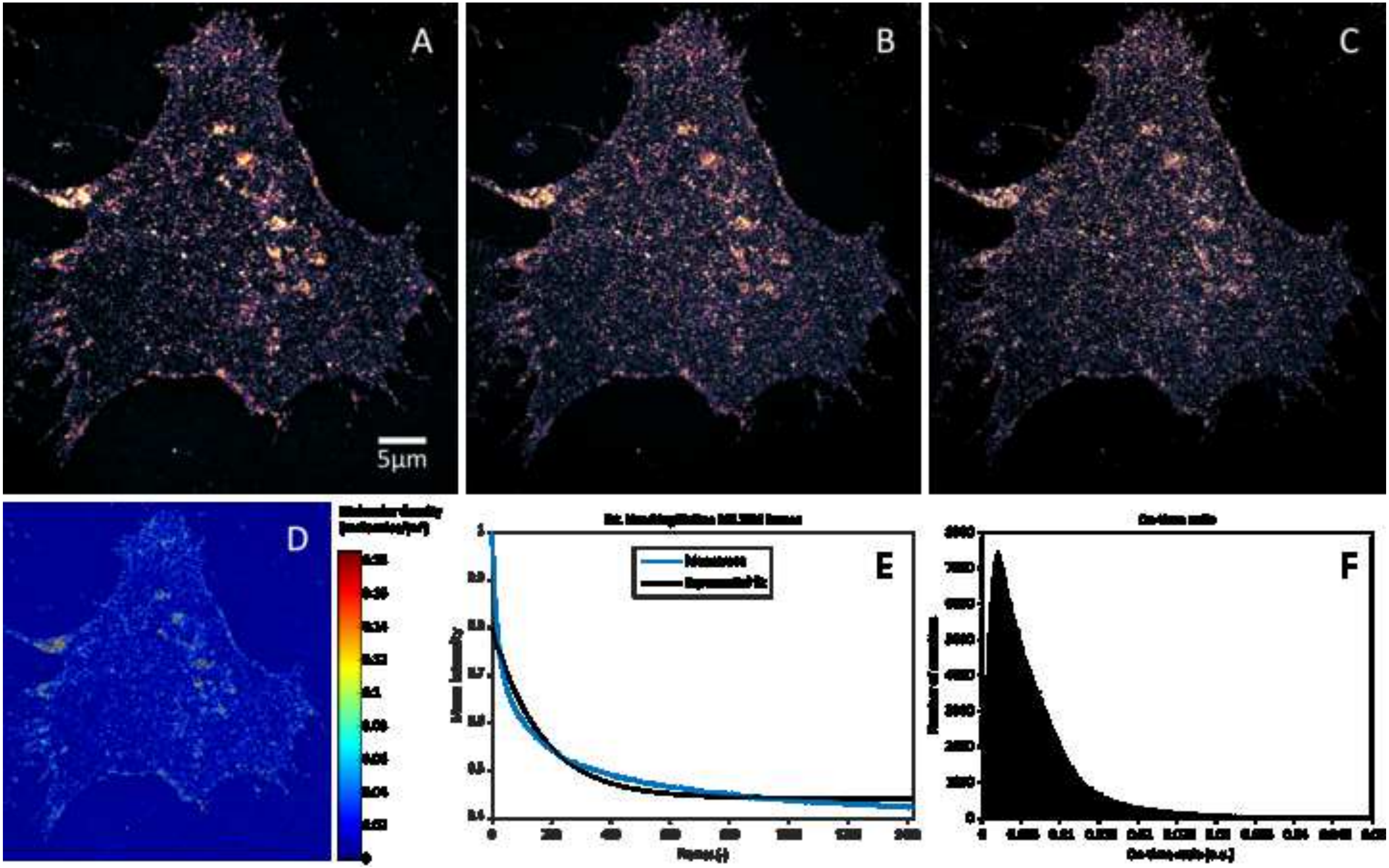

Personal Cover Click here to download Personal Cover Hagen letter to editor.docx

University of Colorado Colorado Springs

Dr. Guy M. Hagen
Biofrontiers
1420 Austin Bluffs Pkwy.
Colorado Springs, CO 80918
Tel. 719-255-3692
ghagen@uccs.edu

Dear Editor:

We would like to submit a manuscript entitled "Quantitative super-resolution single molecule microscopy dataset of YFP-tagged growth factor receptors" for consideration in *GigaScience* as a data note.

Super-resolution microscopy is an important topic today, highlighted by the 2014 Nobel Prize in Chemistry, which was awarded for work in this field. Single-molecule microscopy methods can reach a resolution of approximately 20 nm, or 10 times better than a conventional microscope. This method relies on specialized computer algorithms to analyze the data. To develop the algorithms, researchers require high quality data sets that can be used to test their approaches. These data sets can be rather large, which has prevented their publication up to now. We believe *GigaScience* offers an excellent venue for such publication.

Our datasets include raw data from microscopy experiments on growth factor receptors tagged with yellow fluorescent protein. The growth factor receptor we studied, known as erbB3, is important because this cell surface molecule is present in elevated quantities in several human cancers. We have analyzed the data using two different methods: single molecule localization microscopy, and stochastic optical fluctuation imaging. These two distinct methods are able to produce super-resolution images from a common raw dataset and offer complementary information about the sample.

Figure 1 of the paper shows one of the experiments. Figure 1A and 1B were previously published in the open access paper "ThunderSTORM: a comprehensive ImageJ plug-in for PALM and STORM data analysis and super-resolution imaging" (*Bioinformatics*, Volume 30, Issue 16, 15 August 2014, Pages 2389–2390, https://doi.org/10.1093/bioinformatics/btu202). This earlier paper reported the software developed by my group which we used to analyze the data. We have experienced quite a bit of interest in our software (> 5000 downloads, >150 citations reported by Google Scholar) from a large community of cell biologists and other researchers.

There is an increasing demand for reference datasets which would facilitate adoption of super-resolution imaging methods for a wider audience. *Bioinformatics* does not host large primary datasets such as ours, and so we feel our data fits perfectly into the scope of *GigaScience*. Our datasets include the raw and analyzed data shown in Figure 1, and also three additional datasets. Each dataset includes a conventional microscopy image, the single molecule data (each set consists of several thousand camera frames), super-resolution images created using the two approaches, and data tables consisting of molecular coordinates determined by our software. We also include metadata about the acquisition conditions.

University of Colorado
Colorado Springs

Dr. Guy M. Hagen
Biofrontiers
1420 Austin Bluffs Pkwy.
Colorado Springs, CO 80918
Tel. 719-255-3692
ghagen@uccs.edu

We would like to suggest the following reviewers for the manuscript:

Dr. Daniel Sage
Biomedical Imaging Laboratory
École polytechnique fédérale de Lausanne
Lausanne, Swizerland
daniel.sage@epfl.ch
Dr. Sage has published several papers in this field.

Dr. Udo Birk
Institute of Molecular Biology
Mainz, Germany
u.birk@imb-mainz.de
Dr. Birk is an author of several papers in this field, including a data note article published in *Data in Brief.*

Dr. Ricardo Henriques
MRC laboratory for molecular cell biology
University College London
London, UK
r.henriques@ucl.ac.uk
Dr. Henriques is an author of several papers in this field including those involving development of data analysis algorithms.

Dr. Kyle Douglass
Laboratory of Experimental Biophysics
École polytechnique fédérale de Lausanne
Lausanne, Swizerland
kyle.douglass@epfl.ch
Dr. Douglas is an author of several papers in this field.

Sincerely,

Guy Hagen

Guy M. Hagen
