# Supplementary material for: Quantitative super-resolution single molecule microscopy dataset of YFP-tagged growth factor receptors
Source: Gigascience. 2018 Jan 19;7(3):1–10. doi: 10.1093/gigascience/giy002 (PMC5841371; doi:10.1093/gigascience/giy002)
Supplement: GIGA-D-17-00208_Revision_1.pdf [file giy002_giga-d-17-00208_revision_1.pdf]

GigaScience

Quantitative super-resolution single molecule microscopy dataset of YFP-tagged growth factor receptors

--Manuscript Draft--

**Manuscript Number:**	GIGA-D-17-00208R1
**Full Title:**	Quantitative super-resolution single molecule microscopy dataset of YFP-tagged growth factor receptors
**Article Type:**	Data Note
**Funding Information:**	BioFrontiers Institute, University of Colorado Colorado Springs — Dr Guy Hagen<br>České Vysoké Učení Technické v Praze (CZ) (SGS16/167/OHK3/2T/13) — Dr Karel Fliegel<br>Grantová Agentura České Republiky — Dr Guy Hagen<br>SCIEX (13.183) — Dr Tomáš Lukeš
**Abstract:**	Background: Super-resolution single molecule localization microscopy (SMLM) is a method for achieving resolution beyond the classical limit in optical microscopes (approx. 200 nm laterally). Yellow fluorescent protein (YFP) has been used for super-resolution single molecule localization microscopy, but less frequently than other fluorescent probes. Working with YFP in SMLM is a challenge because a lower number of photons are emitted per molecule compared to organic dyes which are more commonly used. Publically available experimental data can facilitate development of new data analysis algorithms.<br><br>Findings: Four complete, freely available single molecule super-resolution microscopy datasets on YFP-tagged growth factor receptors expressed in a human cell line are presented including both raw and analyzed data. We report methods for sample preparation, for data acquisition, and for data analysis, as well as examples of the acquired images. We also analyzed the SMLM data sets using a different method: super-resolution optical fluctuation imaging (SOFI). The two modes of analysis offer complementary information about the sample. A fifth single molecule super-resolution microscopy dataset acquired with the dye Alexa 532 is included for comparison purposes.<br><br>Conclusion: This dataset has potential for extensive reuse. Complete raw data from SMLM experiments has typically not been published. The YFP data exhibits low signal to noise ratios, making data analysis a challenge. These data sets will be useful to investigators developing their own algorithms for SMLM, SOFI, and related methods. The data will also be useful for researchers investigating growth factor receptors such as ErbB3.
**Corresponding Author:**	Guy Hagen<br><br>UNITED STATES
**Corresponding Author Secondary Information:**	
**Corresponding Author's Institution:**	
**Corresponding Author's Secondary Institution:**	
**First Author:**	Tomáš Lukeš
**First Author Secondary Information:**	
**Order of Authors:**	Tomáš Lukeš<br>Jakub Pospíšil

Powered by Editorial Manager® and ProduXion Manager® from Aries Systems Corporation

	Karel Fliegel
	Theo Lasser
	Guy Hagen
**Order of Authors Secondary Information:**	
**Response to Reviewers:**	please see attached
**Additional Information:**	
**Question**	**Response**
Are you submitting this manuscript to a special series or article collection?	No
**Experimental design and statistics**<br><br>Full details of the experimental design and statistical methods used should be given in the Methods section, as detailed in our Minimum Standards Reporting Checklist. Information essential to interpreting the data presented should be made available in the figure legends.<br><br>Have you included all the information requested in your manuscript?	Yes
**Resources**<br><br>A description of all resources used, including antibodies, cell lines, animals and software tools, with enough information to allow them to be uniquely identified, should be included in the Methods section. Authors are strongly encouraged to cite Research Resource Identifiers (RRIDs) for antibodies, model organisms and tools, where possible.<br><br>Have you included the information requested as detailed in our Minimum Standards Reporting Checklist?	Yes
**Availability of data and materials**<br><br>All datasets and code on which the conclusions of the paper rely must be either included in your submission or deposited in publicly available repositories (where available and ethically appropriate), referencing such data using a unique identifier in the references and in the "Availability of Data and Materials" section of your manuscript.	No

Have you have met the above requirement as detailed in our Minimum Standards Reporting Checklist?	
If not, please give reasons for any omissions below.<br><br>as follow-up to "**Availability of data and materials**<br><br>All datasets and code on which the conclusions of the paper rely must be either included in your submission or deposited in publicly available repositories (where available and ethically appropriate), referencing such data using a unique identifier in the references and in the "Availability of Data and Materials" section of your manuscript.<br><br>Have you have met the above requirement as detailed in our Minimum Standards Reporting Checklist?<br><br>"	we plan to submit the data to GigaDB when the editor assigns the paper to reviewers

Manuscript

Click here to download Manuscript 14 Re-Submit Hagen YFP imaging.docx

Title Quantitative super-resolution single molecule microscopy dataset of YFP-tagged growth factor receptors

Authors Tomáš Lukeš[1], Jakub Pospíšil[2], Karel Fliegel[2], Theo Lasser[1], Guy M. Hagen[3]

Affiliations [1]Laboratoire d'Optique Biomédicale, École Polytechnique Fédérale de Lausanne, CH-1015 Lausanne, Switzerland

[2]Department of Radioelectronics, Faculty of Electrical Engineering, Czech Technical University in Prague, Technická 2, 16627 Prague 6, Czech Republic

[3]UCCS center for the Biofrontiers Institute, University of Colorado at Colorado Springs, 1420 Austin Bluffs Parkway, Colorado Springs, Colorado, 80918, USA

Contact email addresses & ORCID IDs

Tomáš Lukeš lukestom@fel.cvut.cz

Jakub Pospíšil, pospij27@fel.cvut.cz, ORCID: 0000-0003-3615-5752

Karel Fliegel, fliegek@fel.cvut.cz, ORCID: 0000-0001-5737-6736

Theo Lasser, theo.lasser@epfl.ch, ORCID: 0000-0002-4948-7580

Corresponding author, Guy M. Hagen, ghagen@uccs.edu , ORCID: 0000-0002-4802-9481

Abstract

Background: Super-resolution single molecule localization microscopy (SMLM) is a method for achieving resolution beyond the classical limit in optical microscopes (approx. 200 nm laterally). Yellow fluorescent protein (YFP) has been used for super-resolution single molecule localization microscopy, but less frequently than other fluorescent probes. Working with YFP in SMLM is a challenge because a lower number of photons are emitted per molecule compared to organic dyes which are more commonly used. Publically available experimental data can facilitate development of new data analysis algorithms.

Findings: Four complete, freely available single molecule super-resolution microscopy datasets on YFP-tagged growth factor receptors expressed in a human cell line are presented including both raw and analyzed data. We report methods for sample preparation, for data acquisition, and for data analysis, as well as examples of the acquired images. We also analyzed the SMLM data sets using a different method: super-resolution optical fluctuation imaging (SOFI). The two modes of analysis offer complementary information about the sample. A fifth single molecule super-resolution microscopy dataset acquired with the dye Alexa 532 is included for comparison purposes.

Conclusion: This dataset has potential for extensive reuse. Complete raw data from SMLM experiments has typically not been published. The YFP data exhibits low signal to noise ratios, making data analysis a challenge. These data sets will be useful to investigators developing their own algorithms for SMLM, SOFI, and related methods. The data will also be useful for researchers investigating growth factor receptors such as ErbB3.

Keywords: super-resolution microscopy, PALM, STORM, SOFI, YFP, SMLM, single molecule, growth factor receptor, ErbB3, ThunderSTORM

Data description

Context

Fluorescence optical microscopy is one of the most important tools available for the study of biological systems at the cellular level. Unfortunately, due to diffraction phenomena the resolution of fluorescence microscopes in the lateral d dimension is limited to

$$d = \frac{0.61\lambda}{NA}, \qquad (1)$$

where λ is the wavelength of the detected light, and NA is the numerical aperture of the objective lens. As many biological structures within cells are much smaller than this, increasing resolution is of prime importance. Today several methods have been developed which are able to image below the diffraction limit [1,2].

Photoactivated localization microscopy (PALM) [3] was initially accomplished with the photoconvertible fluorescent protein mEOS [4]. A similar method, (direct) stochastic optical reconstruction microscopy (d)STORM utilizes organic dyes [5–8]. In these super-resolution methods, single fluorescent molecules are induced to blink on and off (photoswitching) randomly in the sample. A sensitive camera is used to record an image sequence of the single molecule blinking events, and a computational algorithm is used to fit the imaged point spread functions (PSFs) to a model function [9,10]. By doing so, the coordinates of each molecule can be determined with an uncertainty which is below the diffraction limit [11]. Once enough molecules have been imaged (usually 10^6-10^7 are required, depending on the sample structure [12]), an image can be reconstructed with lateral resolution improved by about a factor of 10. This is done by plotting the coordinates of each molecule in a new image with a much smaller pixel size. Together, this family of methods is known as single molecule localization microscopy (SMLM).

Although PALM experiments were initially performed with fluorescent proteins which are specifically photoconvertible [3], green fluorescent protein (GFP) and its spectral variant yellow fluorescent protein (YFP) are also known to exhibit blinking characteristics [13]. GFP and YFP have been used in SMLM, but less frequently [14–20]. Here we used a modified YFP known as mCitrine [21] for SMLM. The advantage of using mCitrine is that SMLM can be accomplished with a single laser, rather than with separate activation and readout lasers as is done when using mEOS [3]. The question of how fluorophore photophysics influences SMLM experiments is still under investigation [22], but this topic has recently been reviewed fairly comprehensively, taking into account the photoswitching characteristics of fluorescent proteins for SMLM [23].

We used mCitrine to perform SMLM of the growth factor ErbB3 in A431 epithelial carcinoma cells. A431 cells were chosen for this study in part because of their use in previous studies of the ErbB receptor system [24,25], and also because they tend to be very flat and form extended areas of membrane in contact with the coverslip, offering good conditions for SMLM. ErbB3 is a member of the epidermal growth factor receptor (EGFR) family, consisting of ErbB1 (EGFR), ErbB2 (also known as HER2),

ErbB3, and ErbB4. The organization and dynamics of ErbB receptors is an important topic of study because overexpression and unrestrained activation of this family of receptors is implicated in cancer [26], including breast cancer [27]. Long thought to have no kinase activity, ErbB3 has recently been found to exhibit tyrosine kinase activity and to form homodimers and heterodimers with other ErbB receptors [28]. Such heterodimer formation between ErbB molecules can amplify signaling and appears to be an important feature of some cancer cells. In particular the ErbB2/ErbB3 heterodimer appears to be important for tumor cell proliferation in certain breast cancers [29]. High ErbB3 levels have been linked to resistance in cancer therapies which target ErbB1 or ErbB2 [30].

Given the importance of ErbB3 in cancer, an understanding of its organization and dynamics in the plasma membrane of tumor cells is critical. Super-resolution microscopy using single molecule localization reveals the coordinates of each ErbB3 receptor which is tagged with a YFP molecule. This data allows one to explore parameters such as clustering tendencies, an approach used successfully in studies of the T-cell receptor [31].

We have also included an additional single molecule super-resolution microscopy dataset acquired using the dye Alexa 532. This dye is more commonly used in (d)STORM studies [32] and is provided for purposes of comparison of the single molecule parameters. For this experiment we used an Alexa 532-labeled antibody to detect RNA molecules in the nucleus of a HeLa cell as previously described [33]. The raw data is useful in this context because it was acquired with the same microscope setup and detector. Compared to the YFP used in the other datasets, Alexa 532 has higher photon emission rates and exhibits less photobleaching.

The datasets have potential for extensive reuse. Complete raw data from SMLM experiments has typically not been published. The YFP data exhibits low signal to noise ratios, making data analysis a challenge. The data sets will be useful to investigators developing their own algorithms for SMLM, SOFI, and related methods. The data will also be useful for researchers investigating growth factor receptors such as ErbB3, as well as to those investigating other membrane proteins.

Methods

Cell lines and reagents

A431 cells (RRID: CVCL_0037) expressing mCitrine-ErbB3 and HeLa cells (RRID: CVCL_0030) were maintained in phenol red-free DMEM supplemented with 10 % FCS, 100 U/ml penicillin, 100 U/ml streptomycin, and L-glutamate (obtained from Invitrogen, Carlsbad, CA, USA) at 37 °C and 100% humidity. Mowiol 4-88 containing 1,4-diazabicyclo(2.2.2)octane (DABCO) was obtained from Fluka (St. Louis, MO, USA). Mercaptoethylamine (MEA) was obtained from Sigma (St. Louis, MO, USA).

Sample preparation

Prior to SMLM experiments, A431 cells were grown on clean #1.5 coverslips for 12-18 hours. The cells were then washed with PBS, then fixed with 4% paraformaldehyde for 15 minutes at 4 °C. We then mounted the cells on clean slides using mowiol containing DABCO and 50-100 mM MEA, pH 8.5. Before microscopy, the mowiol was allowed to harden for 12-18 hours. The mowiol was freshly prepared according to standard procedures.

For labeling of transcription sites in the cell nucleus, HeLa cells were grown on #1.5 coverslips for 12-18 hours, then incubated for 5 minutes with 5-fluorouridine (Sigma) at a concentration of 10 μM. The cells were then fixed in 2% formaldehyde, permeabilized with 0.1% Triton X-100, and labeled using a mouse monoclonal anti-BrdU antibody (clone BU-33, Sigma). The anti-BrdU antibodies were then detected with a secondary anti-mouse antibody labeled with Alexa 532 (Invitrogen). The cells were mounted using freshly prepared mowiol containing DABCO and 50-100 mM MEA. Before microscopy, the mowiol was allowed to harden for 12-18 hours.

Single molecule microscopy

For SMLM imaging, we used an IX71 microscope equipped with a planapochromatic 100×/1.35 NA oil immersion objective (Olympus, Tokyo, Japan) and a front-illuminated Ixon DU885 EMCCD camera under control of IQ software (Andor, Belfast, Northern Ireland) as previously described [34]. The excitation source was a 400 mW, 473 nm laser (Dragon laser, ChangChun, China), which was coupled to the microscope using a 0.39 NA multimode optical fiber. The fiber output was collimated using a 2 inch diameter, 60 mm FL lens (Thor Labs, Newton, New Jersey). The fiber was coupled into the microscope

using an Olympus IX2-RFAL fluorescence illuminator, resulting in an evenly illuminated field. Fluorescence was observed using an Olympus U-MNIBA3 filter set (excitation 470 – 495 nm, dichroic 505 nm, emission 510 – 550 nm). In each experiment, a sequence of 1,419-10,000 images was acquired with an exposure time of 40 – 100 ms and an EM gain of 50-300. For imaging Alexa 532, we used a 1 W, 532 nm laser (Dragon laser) and an appropriate fluorescence emission filter (569-610 nm, Chroma) as previously described [33].

Data analysis methods

We analyzed the data using ThunderSTORM [9,35] with the default settings. The default settings involve use of a wavelet-based filter for feature enhancement [36], followed by local maximum detection of single molecules in the filtered data. This is followed by fitting molecules in the raw data using a two-dimensional Gaussian function in integrated form [37] using maximum likelihood methods [38]. Gaussian functions have been found to be a good representation of the true PSF of a microscope [39]. For visualization of the results, we use an average shifted histogram approach [40]. If the camera calibration parameters (pixel size, photoelectrons per A/D count, base level, and EM gain) are correct, maximum likelihood fitting of an integrated Gaussian function will correctly return the number of photons detected from each molecule [9,37,38,41]. An integrated two dimensional Gaussian function can be written as

$$\begin{aligned} PSF_{IG}(x, y \mid \theta) &= \theta_N E_x E_y + \theta_b, \\ E_x &= \frac{1}{2}\operatorname{erf}\left(\frac{x-\theta_x+1/2}{\sqrt{2}\theta_\sigma}\right) - \frac{1}{2}\operatorname{erf}\left(\frac{x-\theta_x-1/2}{\sqrt{2}\theta_\sigma}\right), \\ E_y &= \frac{1}{2}\operatorname{erf}\left(\frac{y-\theta_y+1/2}{\sqrt{2}\theta_\sigma}\right) - \frac{1}{2}\operatorname{erf}\left(\frac{y-\theta_y-1/2}{\sqrt{2}\theta_\sigma}\right), \end{aligned} \qquad (2)$$

where θ_x, θ_y are the sub-pixel molecular coordinates, θ_σ is the standard deviation of the Gaussian function (i.e., the width), θ_N is the total number of detected photons emitted by the molecule, and θ_b is the background offset.

Single molecule localization uncertainty

In ThunderSTORM the localization uncertainty is calculated for each detected molecule. This quantity can help one determine whether the molecule was well localized and whether it should be included in the final result. Let $\hat{\theta}_\sigma$ be the standard deviation of a Gaussian function fitted to an imaged PSF in nm, a is the back-projected pixel size in nm (camera pixel size divided by system magnification), $\hat{\theta}_N$ is the estimate of the number of photons detected for a given molecule, and $\hat{b}$ is the background signal level in photons calculated as the standard deviation of the residuals between the raw data and the fitted PSF model. The uncertainty of estimates determined by maximum likelihood methods for the lateral position of a molecule is given by

$$\left(\Delta\hat{\theta}_{xy}\right)^2 = \frac{g\hat{\theta}_{\sigma^2} + a^2/12}{\hat{\theta}_N}\left(1 + 4\tau + \sqrt{\frac{2\tau}{1+4\tau}}\right), \tau = \frac{2\pi\left(\hat{b}^2 + r\right)\left(\hat{\theta}_\sigma^2 + a^2/12\right)}{a^2\hat{\theta}_N}. \quad (3)$$

This formula is a modified form of the Thompson-Larson-Webb equation [11], and was derived by Rieger and Stallinga [42]. Finally, compensation for camera readout noise r and EM gain g was added following Quan, Zeng, and Huang [43], who suggested that when using EMCCD cameras, the correction factors should be set to $r = 0$, $g = 2$, and when using CCD or sCMOS cameras the correction factors should be set to $r = g = 2$.

Super-resolution optical fluctuation imaging

Super-resolution optical fluctuation imaging (SOFI) is based on calculation of spatio-temporal cumulants over the input sequence of camera frames [44]. Assuming a non-fluctuating background and Gaussian additive noise, the n-th order cumulant (for $n \geq 2$ and a time lag τ) can be written as

$$\kappa_n\{I(\mathbf{r},t)\}(\tau) = \sum_{k=1}^{N} \varepsilon_k^n U^n(\mathbf{r}-\mathbf{r}_k)\kappa_n\{s_k(t)\}(\tau), \quad (4)$$

where $I(\mathbf{r},t)$ is the detected intensity at position $\mathbf{r}$ and time t, ε_k is the molecular brightness of k-the emitter, $U^n(\mathbf{r}-\mathbf{r}_k)$ is the PSF at the position $\mathbf{r}_k$, and $s_k(t)$ denotes a normalized fluctuation sequence $s_k(t) \in \{0,1\}$. The PSF is raised to the n-th power, resulting in resolution increased by a factor of $\sqrt{n}$. After reweighting in frequency space, a resolution enhancement factor of n can be achieved [45], scaling

linearly with the cumulant order. SOFI can be applied to any image sequence of stochastically blinking emitters acquired from a conventional widefield microscope if the emitters switch between at least two optically distinguishable states (a dark state and a bright state) and if sampling of the PSF fulfills the Nyquist–Shannon sampling theorem [46]. In comparison to STORM, SOFI tolerates higher densities of emitters and higher blinking rates [47], resulting in improved temporal resolution [48]. SOFI can be applied to the same datasets as SMLM analysis [47,49] offering an interesting complement to SMLM methods. Due to the entirely different image processing methods used, SOFI and SMLM are prone to different artifacts. Applying both processing methods to the same dataset reveals more information about the true structure and properties of the underlying sample. By combining multiple orders of the SOFI analysis, molecular parameters like molecular density, brightness, and on-time ratio can be extracted using the balanced SOFI method (bSOFI) [50]. The on-time ratio ρ_{on} describes the blinking rate of the fluorescent label. Assuming a two state blinking model where the emitter fluctuates between a bright state and a dark state, the on-time ratio is given as [38]

$$\rho_{on} = \frac{\tau_{on}}{\tau_{on} + \tau_{off}}, \qquad (5)$$

where the τ_{on} and τ_{off} are the characteristic lifetimes of the bright state and the dark state, respectively.

SOFI analysis was carried out as reported previously [49]. We used a custom written algorithm (Matlab, The Mathworks) based on the code of our SOFI simulation tool [51] and the bSOFI algorithm [50]. The sequence of camera frames was divided into subsequences of 500 frames each. The subsequences were processed separately in order to minimize the influence of photobleaching and the resulting SOFI images were averaged. Details about photobleaching correction for SOFI have recently been published [52]. SOFI relies on calculating higher order cumulants as described in the previous section. Calculating cumulants raises the molecular brightness to the n-th power (Eq. 3). SOFI's non-linear response to brightness becomes an issue for cumulants of higher than second order where fluorescent spots of high brightness may mask less bright details. The balanced SOFI (bSOFI) algorithm linearizes the response to brightness [50] or to the detected intensity [49]. Throughout this work, the "n-th

order bSOFI image” refers to an image calculated using the n-th order cumulant and applying the subsequent linearization according to the procedure described in [49].

Super-resolution images

Figure 1 shows images of an A431 cell expressing mCitrine-ErbB3 (YFP dataset 1 [53]). Conventional widefield (WF, Fig. 1A), and SMLM (Fig. 1B) results are shown. Fig. 1C shows a color-coded density map, calculated by the bSOFI algorithm. This unique information cannot be obtained by conventional fluorescence microscopy. Fig. 1D shows the 4th order bSOFI image.

INSERT FIGURE 1

Figure 2A shows a histogram of the number of photons detected from each YFP molecule (“intensity” in ThunderSTORM) for the cell shown in Fig 1. Fig. 2B shows a histogram of the localization uncertainty determined for each molecule for the cell shown in Fig 1. The localization uncertainty was calculated using Eq. 3. The two histograms were calculated using the *plot histogram* command in ThunderSTORM.

INSERT FIGURE 2

Table 1 shows a list of quantitative parameters for the first 10 detected molecules as reported by ThunderSTORM for the experiment shown in Figure 1. Sigma (nm) is the standard deviation of the two-dimensional integrated Gaussian function fitted to the molecule, intensity (photons) is the number of photons detected from the molecule, offset (photons) is the background offset, SD of background (photons) is the standard deviation of the background, and localization uncertainty (nm) is the result of Equation 2 for each molecule. Recall that the full width at half max (FWHM) of a Gaussian function is related to its standard deviation by $FWHM=2.35\sigma$. The variation in parameters between molecules is usually attributed to differences in the local environment of each molecule such as oxygen concentration, and to factors such as the fluorophore orientation.

Table 1 Quantitative parameters for the first 10 detected molecules as reported by ThunderSTORM for the experiment shown in Fig. 1.

Molecule number	Camera frame	x (nm)	y (nm)	sigma (nm)	intensity (photons)	offset (photons)	SD of Background (photons)	localization uncertainty (nm)

1	1	3743.17	28005.63	81.53	942	108	25	17.41
2	1	3880.95	31519.89	155.09	2014	68	21	23.33
3	1	4150.78	32662.21	60.03	433	81	21	17.75
4	1	4289.06	28407.32	36.90	407	155	32	12.97
5	1	4310.28	28737.99	103.00	1567	142	34	21.61
6	1	4615.06	23832.74	89.18	1186	73	22	14.60
7	1	4695.34	30060.77	102.05	1266	122	28	22.17
8	1	4812.40	30994.57	101.18	1051	115	24	22.61
9	1	4827.01	25960.59	83.02	717	80	20	19.35
10	1	5037.67	28686.08	149.32	2293	121	33	29.85

Figure 3 shows WF imaging of an A431 cell (Fig. 3A), along with identification of single molecules by ThunderSTORM (Fig. 3B, indicated by red dots), and the reconstructed SMLM result (Fig. 3C) (YFP dataset 2, [53]).

INSERT FIGURE 3

Figure 4 shows WF imaging (Fig. 4A), and the reconstructed SMLM result (Fig. 4B) (YFP dataset 3, [53]). Figure 5 shows SOFI analysis for the cell shown in Figure 4. Second, third, and fourth order bSOFI images are shown in Fig. 5A-5C, as well as a density map (Fig 5D), photobleaching profile (Fig. 5E), and molecular on-time ratio (Fig 5F), where second, third, and fourth denotes the order of the cumulant used during the calculation of the bSOFI image. With increasing cumulant order of the SOFI analysis, spatial resolution generally increases, but the signal-to-background ratio (SBR) limits the spatial resolution achievable in practice. The situation is shown in detail in Fig 6B-D and in the line profiles in Fig 6F-H. The fourth order bSOFI image (Fig 6D) has higher spatial resolution compared to the second and third order bSOFI images (Fig 6B-C). The dashed lines in Fig 6F show the average value of the background of the bSOFI images, which increases for increasing order of SOFI analysis. In other words, increasing the cumulant order leads to a decrease in SBR which hampers the resolution enhancement. Note that we calculate linearized SOFI as previously described [49,50]. In the case of a relatively low density of emitters (Fig 6 F, H), SMLM achieved better spatial resolution. On the other hand, in Fig 6G, the SMLM

analysis does not agree with the result from SOFI, suggesting that the local density of emitters was too high for successful single molecule identification and fitting in that particular location of the cell membrane.

Comparing the density maps in Fig 1D and Fig 5D, the sample in Fig. 5D exhibits an average density approximately 1.8 fold higher. The presence of more emitters in the sample (Fig 5D) leads to higher brightness which is likely the reason why the bSOFI image reconstruction was still successful despite the lower number of input frames.

INSERT FIGURE 4

INSERT FIGURE 5

INSERT FIGURE 6

Table 2 shows a summary of the imaging conditions and quantitative parameters for the YFP and Alexa 532 datasets. Also shown are the relevant camera settings. The camera setting information should be entered into ThunderSTORM's camera setup tab to ensure correct results.

Table 2 Summary of imaging conditions and quantitative parameters for the SMLM datasets.

Data	Exp. time, ms	Pixel size, nm	e^- per A/D count[1]	Base level, A/D counts	EM Gain	Frames	Total number of detections	Sigma, nm mean+/-SD	Loc. uncertainty, nm mean+/-SD
YFP data 1 (Fig. 1)	50	80	3.6	414	150	10,000	482,778	86.9+/-29.0	25.6+/-8.6
YFP data 2 (Fig . 2)	100	80	3.6	414	50	6,366	224,175	84.1+/-26.3	27.3+/-8.7
YFP data 3 (Figs. 4, 5, 6)	50	80	3.6	414	150	1,419	452,498	84.2+/-24.3	28.9+/-8.1
YFP data 4	100	80	3.6	414	100	3,922	159,463	81.6+/-24.1	25.9+/-8.0
Alexa 532 data	30	80	1.5	396	50	20,000	1,128,322	121.5+/-47.6	20.6+/-7.5

[1]photoelectrons per analog to digital converter count

Re-use potential

Super-resolution microscopy algorithms are under active development [10]. Researchers engaged in algorithm development may use this dataset to help develop and fine tune their methods. Since the true positions of the molecules remain unknown, the results from ThunderSTORM may be taken as the

reference data for comparison purposes. ThunderSTORM offers an analysis tool which compares reference data and experimental data and computes several quantities which can be used to quantitatively evaluate algorithm performance. A detailed example of use is provided in the supplementary information.

Availability of source code and requirements

Project name: ThunderSTORM v1.3

Project home page: http://zitmen.github.io/thunderstorm/

Operating system: platform independent

Programming language: Java

Other requirements: Image J https://imagej.nih.gov/ij/

License: GNU General Public License v3.0

Availability of data

All raw and analyzed data are available in the *GigaScience* repository, GigaDB [53].

Abbreviations

(d)STORM, (direct) stochastic optical reconstruction microscopy; FWHM, full width at half maximum; GFP, green fluorescent protein, NA, numerical aperture; PALM, photoactivated localization microscopy; PSF, point spread function; SMLM, single molecule localization microscopy; SOFI, stochastic optical fluctuation imaging; WF, wide field; YFP, yellow fluorescent protein.

Ethics approval and consent to participate

Not applicable

Competing interests

The authors declare that they have no competing interests.

Funding

This work was supported by the UCCS center for the University of Colorado BioFrontiers Institute, by the Czech Science Foundation, and by Czech Technical University in Prague (grant number SGS16/167/OHK3/2T/13). T.L. acknowledges a SCIEX scholarship (project code 13.183). The funding sources had no involvement in study design; in the collection, analysis and interpretation of data; in the writing of the report; or in the decision to submit the article for publication.

Author Contributions

TL: analyzed data, developed computer code, wrote the paper

JP: analyzed data, developed computer code

KF: supervised research

TL: supervised research

GH: conceived project, acquired data, analyzed data, supervised research, wrote the paper

Acknowledgements

Epithelial carcinoma A431 cells expressing mCitrine-ErbB3 were a kind gift from Dr. Donna Arndt-Jovin and Dr. Tom Jovin of the Max Planck Institute for Biophysical Chemistry (Göttingen, Germany). We thank Peter W. Winter for assistance with microscopy, and Pavel Křížek, Josef Borkovec, Zdeněk Švindrych, and Martin Ovesný for assistance with microscopy, data analysis, and programming. We thank Evgeny Smirnov for assistance with sample preparation.

References

[1] Huang B, Bates M, Zhuang X. Super-resolution fluorescence microscopy. Annu. Rev. Biochem. 2009;78:993–1016.

[2] Hell SW, Sahl SJ, Bates M, Zhuang X, Heintzmann R, Booth MJ, et al. The 2015 super-resolution

microscopy roadmap. J. Phys. D. Appl. Phys. 2015;48:443001.

[3] Betzig E, Patterson GH, Sougrat R, Lindwasser OW, Olenych S, Bonifacino JS, et al. Imaging intracellular fluorescent proteins at nanometer resolution. Science. 2006;313:1642–5.

[4] Wiedenmann J, Ivanchenko S, Oswald F, Schmitt F, Röcker C, Salih A, et al. EosFP, a fluorescent marker protein with UV-inducible green-to-red fluorescence conversion. Proc. Natl. Acad. Sci. U. S. A. 2004;101:15905–10.

[5] Rust MJ, Bates M, Zhuang X. Sub-diffraction-limit imaging by stochastic optical reconstruction microscopy (STORM). Nat. Methods. 2006;3:793–5.

[6] Huang B, Wang W, Bates M, Zhuang X. Three-dimensional super-resolution imaging by stochastic optical reconstruction microscopy. Science. 2008;319:810–3.

[7] Heilemann M, van de Linde S, Schüttpelz M, Kasper R, Seefeldt B, Mukherjee A, et al. Subdiffraction-resolution fluorescence imaging with conventional fluorescent probes. Angew. Chemie Int. Ed. 2008;47:6172–6.

[8] Dempsey GT, Vaughan JC, Chen KH, Bates M, Zhuang X. Evaluation of fluorophores for optimal performance in localization-based super-resolution imaging. Nat. Methods. 2011;8:1–14.

[9] Ovesný M, Křížek P, Borkovec J, Švindrych Z, Hagen GM. ThunderSTORM: A comprehensive ImageJ plug-in for PALM and STORM data analysis and super-resolution imaging. Bioinformatics. 2014;30.

[10] Sage D, Kirshner H, Pengo T, Stuurman N, Min J, Manley S, et al. Quantitative evaluation of software packages for single-molecule localization microscopy. Nat. Methods. 2015;12:717–24.

[11] Thompson RE, Larson DR, Webb WW. Precise nanometer localization analysis for individual fluorescent probes. Biophys. J. 2002;82:2775–2783.

[12] Fox-Roberts P, Marsh R, Pfisterer K, Jayo A, Parsons M, Cox S. Local dimensionality determines imaging speed in localization microscopy. Nat. Commun. 2017;8:13558.

[13] Dickson RM, Cubitt AB, Tsien RY, Moerner WE. On/off blinking and switching behaviour of single molecules of green fluorescent protein. Nature. 1997;388:355–358.

[14] Lemmer P, Gunkel M, Baddeley D, Kaufmann R, Urich A, Weiland Y, et al. SPDM: light microscopy with single-molecule resolution at the nanoscale. Appl. Phys. B Lasers Opt. 2008;93:1–12.

[15] Lemmer P, Gunkel M, Weiland Y, Muller P, Baddeley D, Kaufmann R, et al. Using conventional fluorescent markers for far-field fluorescence localization nanoscopy allows resolution in the 10-nm range. J. Microsc. 2009;235:163–71.

[16] Biteen JS, Thompson MA, Tselentis NK, Bowman GR, Shapiro L, Moerner WE. Super-resolution imaging in live Caulobacter crescentus cells using photoswitchable EYFP. Nat. Methods. 2008;5:947–9.

[17] Lew MD, Lee SF, Ptacin JL, Lee MK, Twieg RJ, Shapiro L, et al. Three-dimensional superresolution colocalization of intracellular protein superstructures and the cell surface in live Caulobacter crescentus. Proc. Natl. Acad. Sci. U. S. A. 2011;108:E1102–10.

[18] Jusuk I, Vietz C, Raab M, Dammeyer T, Tinnefeld P. Super-Resolution Imaging Conditions for enhanced Yellow Fluorescent Protein (eYFP) Demonstrated on DNA Origami Nanorulers. Sci. Rep. 2015;5:14075.

[19] Křížek P, Raška I, Hagen GM. Minimizing detection errors in single molecule localization microscopy. Opt. Express. 2011;19:3226–35.

[20] Kaufmann R, Piontek J, Grüll F, Kirchgessner M, Rossa J, Wolburg H, et al. Visualization and quantitative analysis of reconstituted tight junctions using localization microscopy. PLoS One. 2012;7:e31128.

[21] Griesbeck O, Baird GS, Campbell RE, Zacharias DA, Tsien RY. Reducing the environmental sensitivity of yellow fluorescent protein. J. Biol. Chem. 2001;276:29188–29194.

[22] Pennacchietti F, Gould TJ, Hess ST. The role of probe photophysics in localization-based superresolution microscopy. Biophys. J. 2017;113:2037–54.

[23] Shcherbakova DM, Sengupta P, Lippincott-Schwartz J, Verkhusha V V. Photocontrollable fluorescent proteins for superresolution imaging. Annu. Rev. Biophys. 2014;43:303–29.

[24] Nagy P, Arndt-Jovin DJ, Jovin TM. Small interfering RNAs suppress the expression of endogenous and GFP-fused epidermal growth factor receptor (erbB1) and induce apoptosis in erbB1-overexpressing cells. Exp. Cell Res. 2003;285:39–49.

[25] Hagen GM, Caarls W, Lidke KA, De Vries AHB, Fritsch C, Barisas BG, et al. Fluorescence recovery after photobleaching and photoconversion in multiple arbitrary regions of interest using a programmable array microscope. Microsc. Res. Tech. 2009;72:431–40.

[26] Yarden Y, Sliwkowski. Untangling the ErbB signaling network. Nat. Rev. Mol. Cell Biol. 2001;2:127–37.

[27] Naidu R, Yadav M, Nair S, Kutty MK. Expression of c-erbB3 protein in primary breast carcinomas. Br. J. Cancer. 1998;78:1385–90.

[28] Steinkamp MP, Low-Nam ST, Yang S, Lidke KA, Lidke DS, Wilson BS. ErbB3 is an active tyrosine kinase capable of homo- and heterointeractions. Mol. Cell. Biol. 2014;34:965–77.

[29] Holbro T, Beerli RR, Maurer F, Koziczak M, Barbas CF, Hynes NE. The ErbB2/ErbB3 heterodimer functions as an oncogenic unit: ErbB2 requires ErbB3 to drive breast tumor cell proliferation. Proc. Natl. Acad. Sci. U. S. A. 2003;100:8933–8938.

[30] Sithanandam G, Anderson LM. The ErbB3 receptor in cancer and cancer gene therapy. Cancer Gene Ther. 2008;15:413–48.

[31] Williamson DJ, Owen DM, Rossy J, Magenau A, Wehrmann M, Gooding JJ, et al. Pre-existing clusters of the adaptor Lat do not participate in early T cell signaling events. Nat Immunol. 2011;12:655–62.

[32] Heilemann M, Linde S van de, Mukherjee A, Sauer M. Super-resolution imaging with small organic fluorophores. Angew. Chemie Int. Ed. 2009;48:6903–6908.

[33] Smirnov E, Borkovec J, Kováčik L, Svidenská S, Schröfel A, Skalníková M, et al. Separation of replication and transcription domains in nucleoli. J. Struct. Biol. 2014;188:259–66.

[34] Křížek P, Raška I, Hagen GM. Flexible structured illumination microscope with a programmable illumination array. Opt. Express. 2012;20:24585–99.

[35] Ovesný M, Křížek P, Borkovec J, Švindrych Z, Hagen GM. Image analysis for single-molecule localization microscopy. In: Diaspro A, Marc A. M. J. van Zandvoort, editors. Super-Resolution Imaging Biomed., Boca Raton, Florida: CRC Press; 2016, p. 79–97.

[36] Izeddin I, Boulanger J, Racine V, Specht CG, Kechkar A, Nair D, et al. Wavelet analysis for single molecule localization microscopy. Opt. Express. 2012;20:2081–95.

[37] Huang F, Schwartz SL, Byars JM, Lidke KA. Simultaneous multiple-emitter fitting for single molecule super-resolution imaging. Biomed. Opt. Express. 2011;2:1377–93.

[38] Mortensen KI, Churchman LS, Spudich JA, Flyvbjerg H. Optimized localization analysis for single-molecule tracking and super-resolution microscopy. Nat. Methods. 2010;7:377–381.

[39] Stallinga S, Rieger B. Accuracy of the gaussian point spread function model in 2D localization microscopy. Opt. Express. 2010;18:24461–76.

[40] Scott DW. Averaged shifted histograms: effective nonparametric density estimators in several dimensions. Ann. Stat. 1985;13:1024–40.

[41] Smith CS, Joseph N, Rieger B, Lidke KA. Fast, single-molecule localization that achieves theoretically minimum uncertainty. Nat. Methods. 2010;7:373–375.

[42] Rieger B, Stallinga S. The lateral and axial localization uncertainty in super-resolution light microscopy. Chemphyschem. 2014;15:664–70.

[43] Quan T, Zeng S, Huang Z-L. Localization capability and limitation of electron-multiplying charge-coupled, scientific complementary metal-oxide semiconductor, and charge-coupled devices for superresolution imaging. J. Biomed. Opt. 2010;15:66005.

[44] Dertinger T, Colyer R, Iyer G, Weiss S, Enderlein J. Fast, background-free, 3D super-resolution optical fluctuation imaging (SOFI). Proc. Natl. Acad. Sci. U. S. A. 2009;106:22287–22292.

[45] Dertinger T, Colyer R, Vogel R, Enderlein J, Weiss S. Achieving increased resolution and more pixels with superresolution optical fluctuation imaging (SOFI). Opt. Express. 2010;18:18875–85.

[46] Heintzmann R. Band-limit and appropriate sampling in microscopy. In: Celis Julio E, editor. Cell Biol. A Lab. Handb., Elsevier Academic Press; 2006, p. 29–36.

[47] Geissbuehler S, Dellagiacoma C, Lasser T. Comparison between SOFI and STORM. Biomed. Opt. Express. 2011;2:408–20.

[48] Geissbuehler S, Sharipov A, Godinat A, Bocchio NL, Sandoz PA, Huss A, et al. Live-cell multiplane three-dimensional super-resolution optical fluctuation imaging. Nat. Commun. 2014;5:5830.

[49] Deschout H, Lukes T, Sharipov A, Szlag D, Feletti L, Vandenberg W, et al. Complementarity of PALM and SOFI for super-resolution live-cell imaging of focal adhesions. Nat. Commun. 2016;7:13693.

[50] Geissbuehler S, Bocchio NL, Dellagiacoma C, Berclaz C, Leutenegger M, Lasser T. Mapping molecular statistics with balanced super-resolution optical fluctuation imaging (bSOFI). Opt. Nanoscopy. 2012;1:4.

[51] Girsault A, Lukeš T, Sharipov A, Geissbuehler S, Leutenegger M, Vandenberg W, et al. SOFI simulation tool: A software package for simulating and testing super-resolution optical fluctuation imaging. PLoS One. 2016;11:e0161602.

[52] Peeters Y, Vandenberg W, Duwé S, Bouwens A, Lukeš T, Ruckebusch C, et al. Correcting for photodestruction in super-resolution optical fluctuation imaging. Sci. Rep. 2017;7:July 2017.

[53] Lukeš, T; Pospíšil, J; Fliegel, K; Lasser, T; Hagen, G, M (2018): Supporting data for “Quantitative super-resolution single molecule microscopy dataset of YFP-tagged growth factor receptors” GigaScience Database. http://dx.doi.org/10.5524/100400

FIGURE CAPTIONS

Fig. 1 Super-resolution imaging of mCitrine-ErbB3 in A431 cells. (A) Conventional widefield. (B) SMLM. (C) Molecular density map. (D) 4th order bSOFI.

Fig. 2 Quantification of molecular parameters from the experiment shown in Fig. 1. (A) Histogram of the number of photons detected from each YFP molecule. (B) Histogram of the localization uncertainty calculated for each YFP molecule.

Fig. 3 Super-resolution imaging of mCitrine-ErbB3 in A431 cells. (A) Conventional widefield. (B) Single frame of SMLM with detections indicated with red dots. (C) SMLM reconstruction.

Fig. 4 Super-resolution imaging of mCitrine-ErbB3 in A431 cells. (A) Conventional widefield. (B) SMLM reconstruction.

Fig 5 Super-resolution imaging of mCitrine-ErbB3 in A431 cells. (A,) (B) and (C) are second, third and fourth order bSOFI reconstruction, respectively. (D) Molecular density map estimated using bSOFI (E) Mean intensity trace of the raw image sequence (blue) with the exponential fit (black) used for photobleaching correction. (F) Histogram of the on-time ratio estimated using bSOFI algorithm.

Fig 6 Super-resolution imaging of mCitrine-ErbB3 in A431 cells. (A) Conventional widefield. Region of interest marked in A by the yellow square processed by 2nd order bSOFI (B), 3rd order bSOFI (C), 4th order bSOFI (D), SMLM (E). (F-H) Line profiles along the cuts 1-1’, 2-2’ and 3-3’ which correspond to examples of low density of emitters (F), high density (G), and medium density (H), respectively. Dashed lines in (F) represent the average value of the background of bSOFI images.

Figure1

Click here to download Figure Fig_1.png

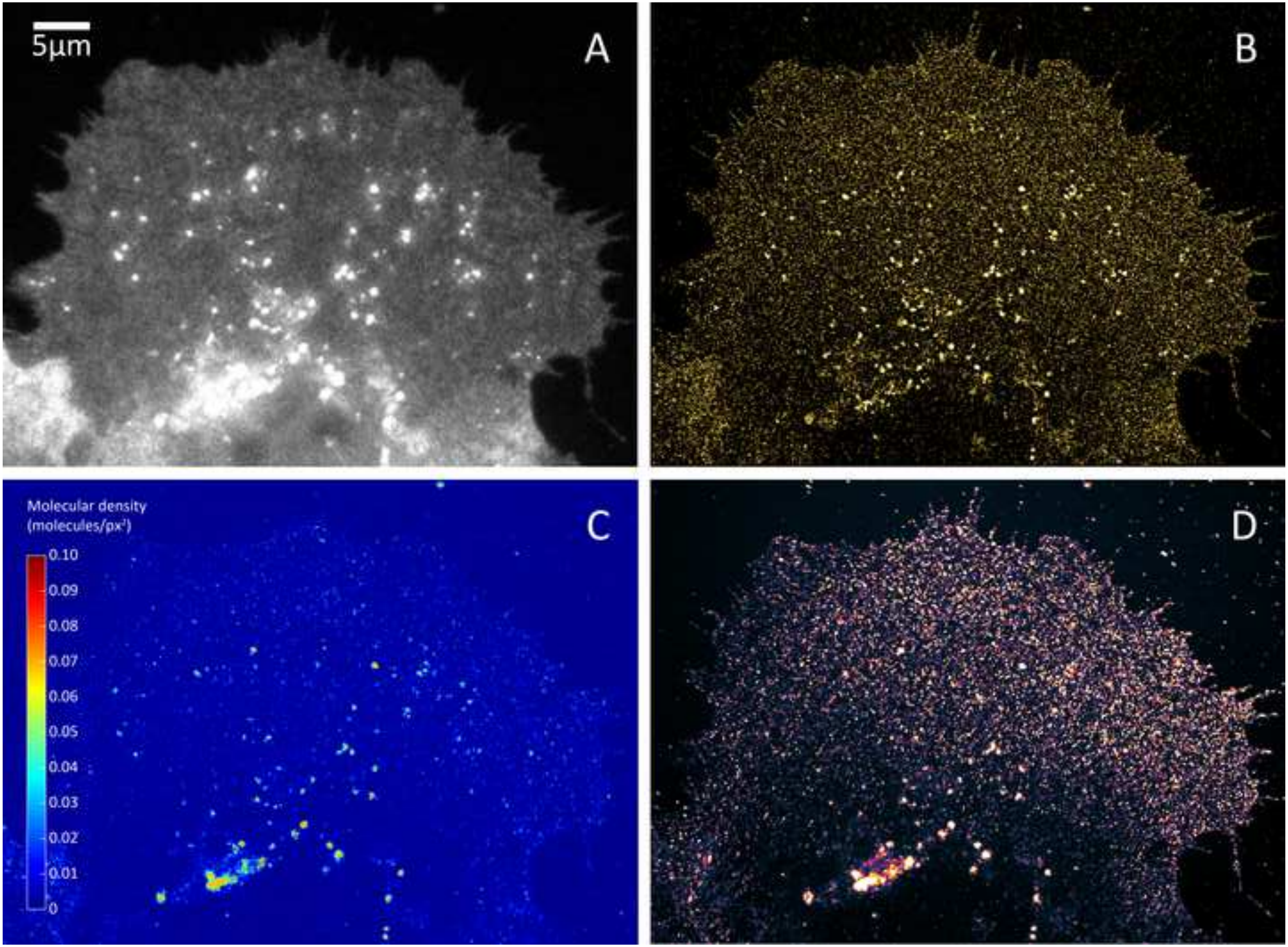

Figure2 Click here to download Figure Fig_2.png

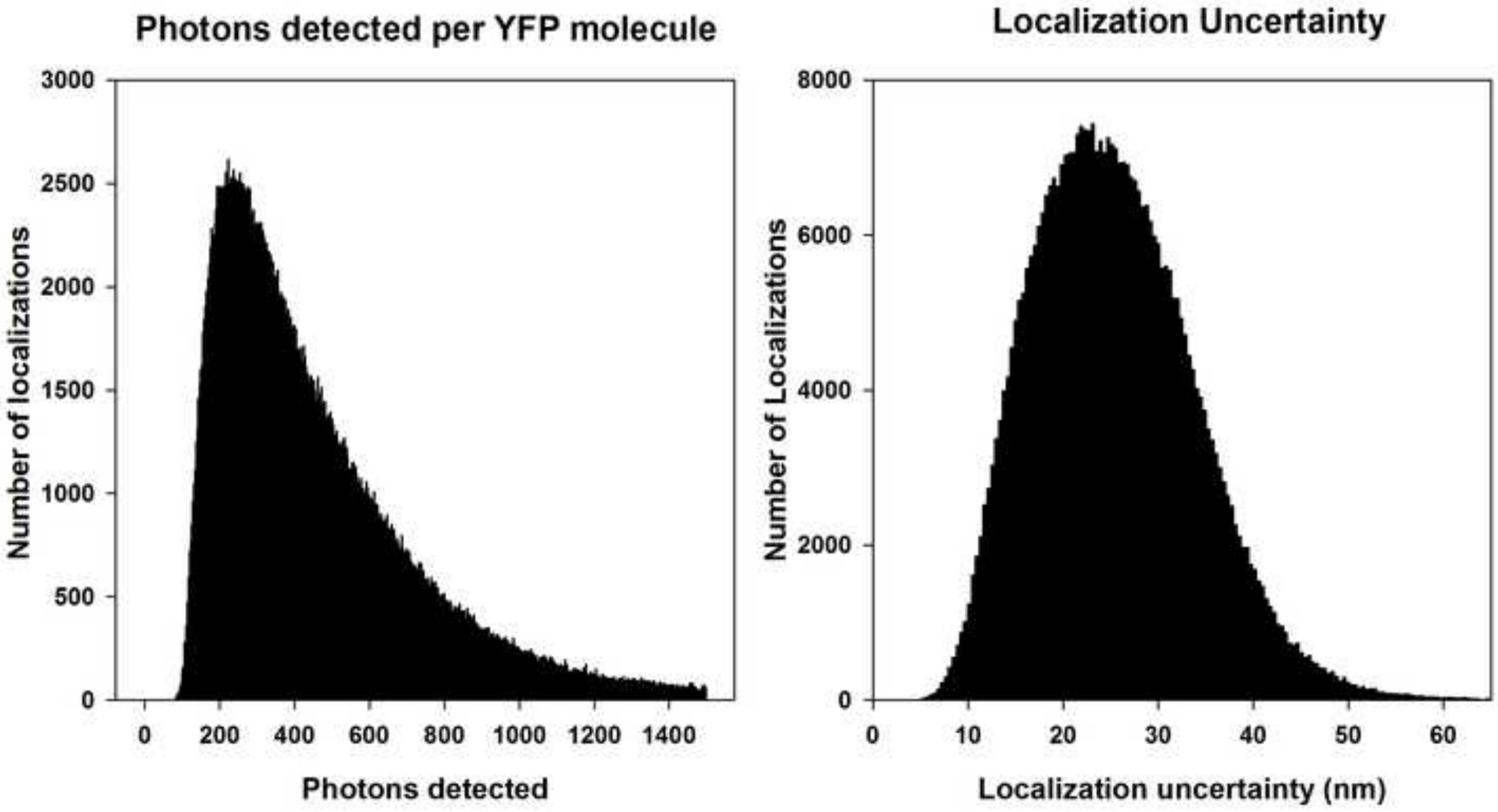

Figure3

Click here to download Figure Fig_3.png

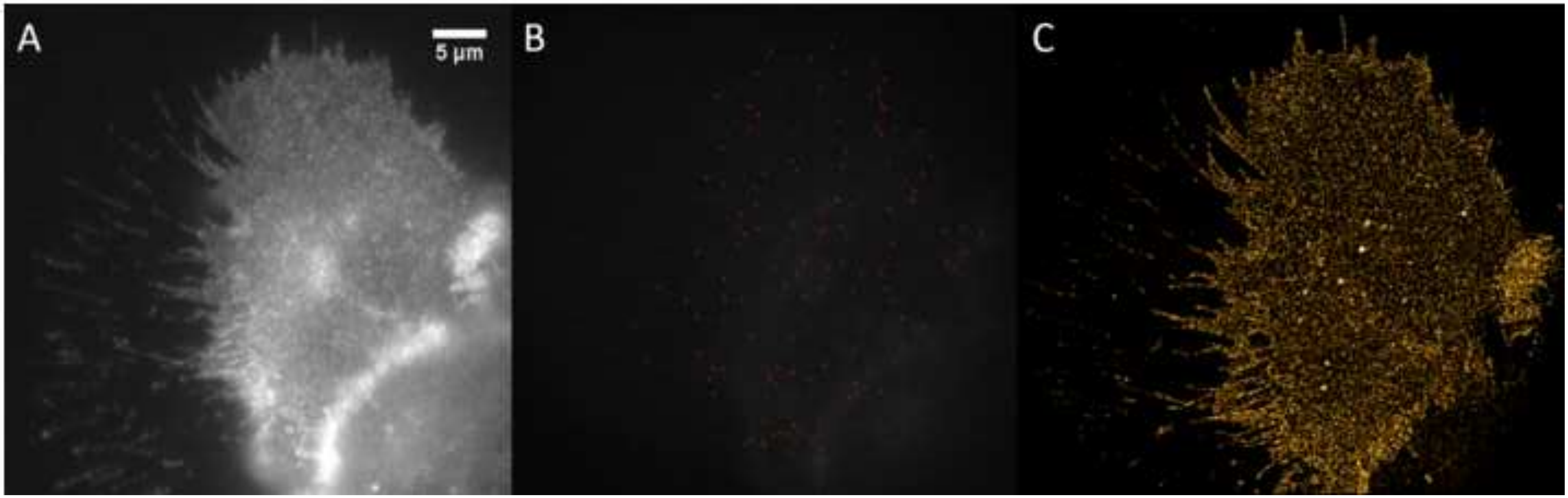

 Click here to download Figure Fig_4.png

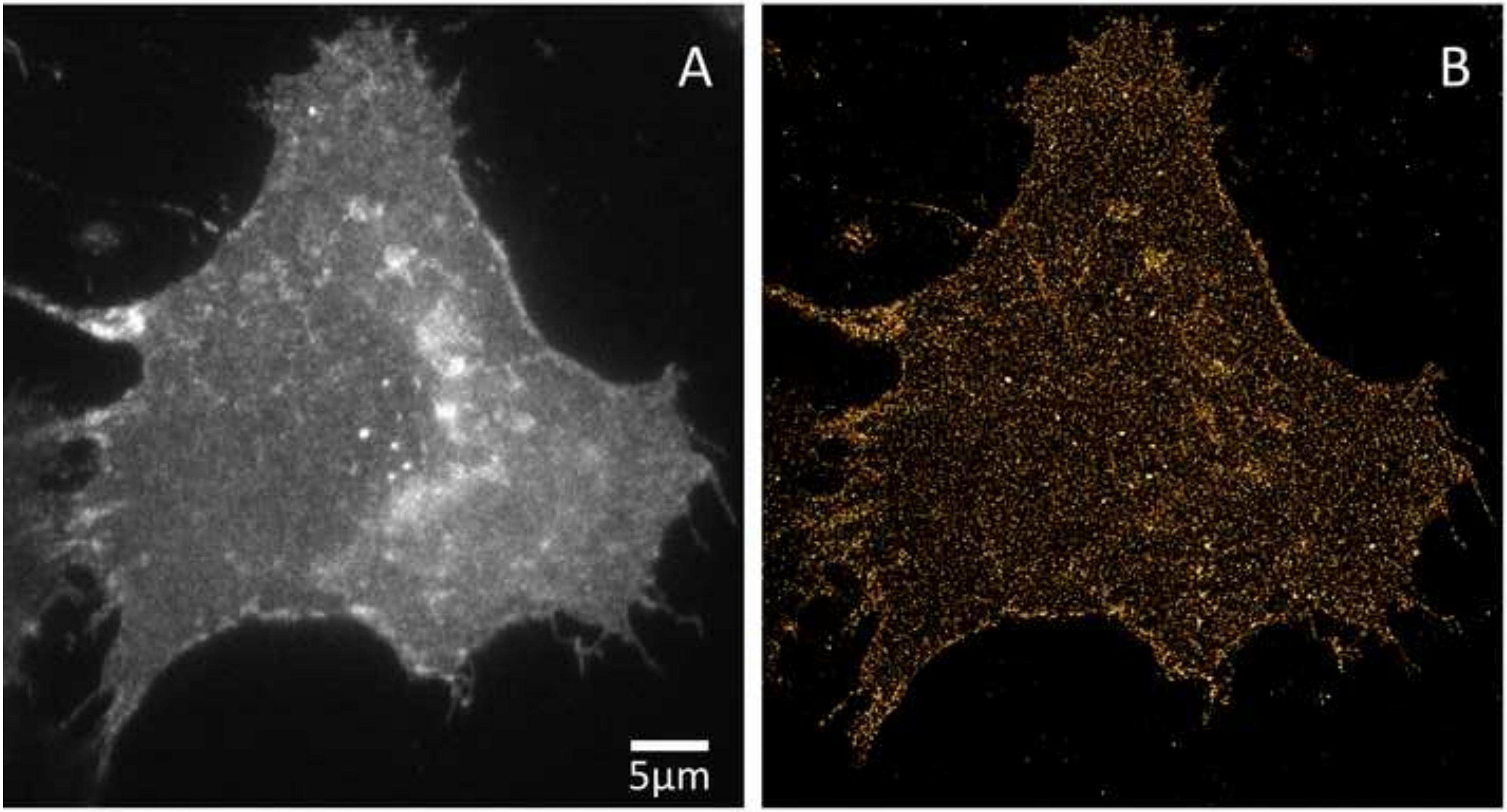

Figure5

Click here to download Figure Fig_5.png

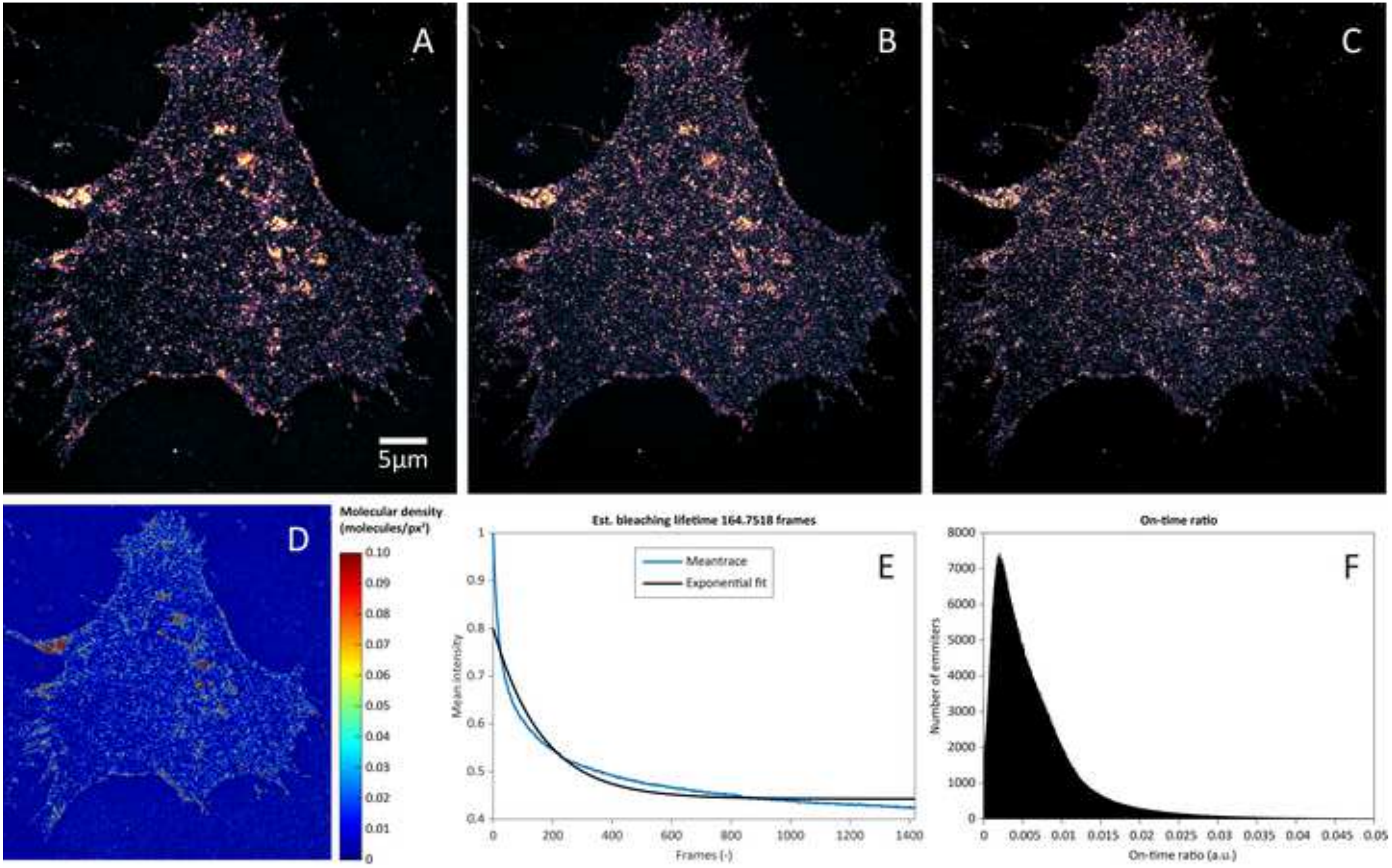

Figure6

Click here to download Figure Fig_6.png

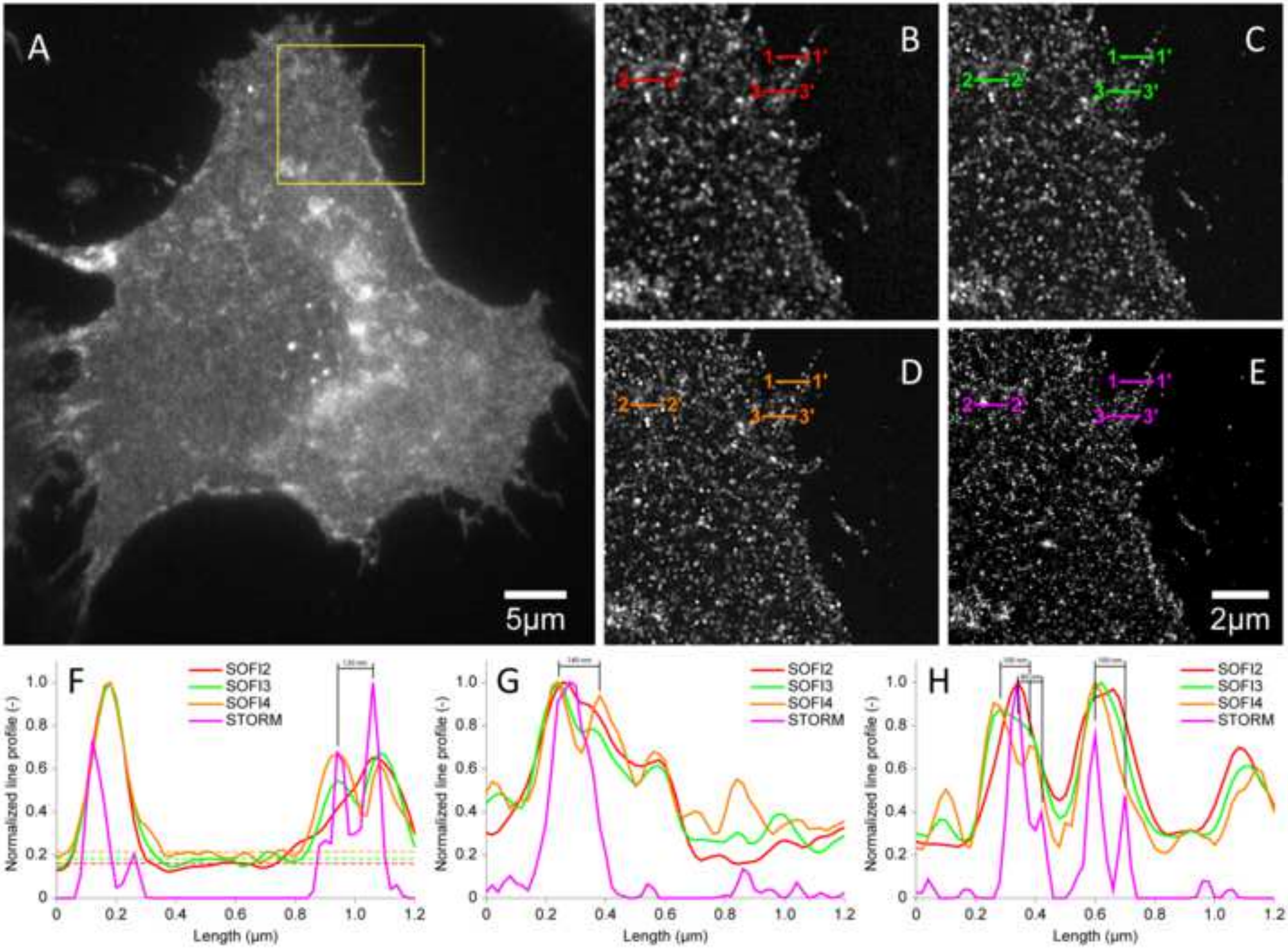

Click here to access/download
Supplementary Material
Supplement.pdf

University of Colorado Colorado Springs

Dr. Guy M. Hagen
Biofrontiers Center
1420 Austin Bluffs Pkwy.
Colorado Springs, CO 80918
ghagen@uccs.edu

Dear Editor:

Thank you for your positive evaluation of our paper. We have carefully revised the paper according to your own and the reviewer's comments, and would like to reply as follows. We hope that with these changes, our paper will be ready for publication in *GigaScience.*

Editor's comments:

From an editorial perspective we would ask for a bit more detail on potential uses of this data and more methodological detail for reproducibility. We recommend Data Notes have a Re-use potential section at the end of the paper. Instructions and suggestions of downstream applications that may help other researchers with reuse of the data are also encouraged. This section can also promote discussion on possible ways the data presented might be used in or have a relationship with other areas of research that may not be directly apparent in the work.

We have prepared a new supplement for the manuscript which offers detailed information about how the data sets may be re-used. We have included background information, references, and a completed example of use.

In addition, for antibodies, cell lines, and any other entities please search in the SciCrunch.org database to see if they have a RRID (Research Resource Identification Initiative ID) number, and include this in your manuscript. This will facilitate reproducibility and re-use of your work.

We included the RRID for the two cell lines we used. The antibody we used was not in the SciCrunch.org database, so we indicated the clone number.

For reproducibility we strongly recommend you use protocols.io to enter any protocols in a stepwise manner, and this will generate a discoverable and citable DOI that will be integrated into the paper.

Our methods and protocols have been published previously[1–4] as indicated in the manuscript. The publication for the software ThunderSTORM[2] includes supplementary information (40 pages) and a user's guide (26 pages) with extremely detailed information about use of the software and complete documentation of the methodology and algorithms. The project website also contains very detailed information about use of the software (https://github.com/zitmen/thunderstorm/wiki). The sample preparation steps are considered to be quite standard at this point. Because of these factors, we do not wish to add an additional protocol to protols.io.

Reviewer #1:

1) Can the authors comment on where mCitrine sits, relative to other fluorescent proteins, in terms of an appropriate choice for the purpose of SMLM? What parameters should be considered?

The reason we used this particular fluorescent protein for the presented data is its low photon emission rate which creates a challenge for localization of emitters by SMLM techniques. The aim of our work was to offer a dataset with difficult SMLM images (low SNR) for evaluation of analytical tools under suboptimal conditions. However mCitrine is probably not the best choice for SMLM experiments when compared to newer fluorescent proteins such as mEos. On the other hand, SMLM experiments employing mCitrine can be accomplished with a single laser, simplifying the microscope setup.

We added the following text and references to the manuscript (note that the references are re-numbered in this letter as compared to the manuscript):

"The advantage of using mCitrine is that SMLM can be accomplished with a single laser, rather than with separate activation and readout lasers as is done when using mEOS[5]. The question of how fluorophore photophysics influences SMLM experiments is still under investigation[6], but this topic has recently been reviewed fairly comprehensively, taking into account the photoswitching characteristics of fluorescent proteins for SMLM[7]."

2) In the 'Context' section (paragraph from Line 56), can the authors expand on why A431 cells and ErbB3 were used and what their biological significance is? Why might super-resolution be a useful approach for studying ErbB3 - what extra information can be gained from such approaches when compared to diffraction limited techniques and can we see this in the images presented?

ErbB3 is structurally related to a well-described onco-protein, ErbB1, the epidermal growth factor receptor. ErbB3 has been found to be overexpressed or present in mutated forms in several forms of cancer. ErbB3 forms heterodimers with ErbB1 or ErbB2 (also known as HER2). A431 cells are cancer cells of epithelial origin. A431 cells are easy to transfect, a property useful for technology oriented and preliminary biological studies. As seen in Figure 1, diffraction limited microscopy provides only coarse details about the distribution of ErbB3 molecules on these cells. SMLM uncovers potential clustering of ErbB3 in these cells. For example, in the future, correlation of tumorigenic potential of ErbB3 in clusters with its randomly distributed counterparts can be studied. ErbB3 co-localization with downstream effectors, and many other open questions related to the nanoscopic organization of oncogenic receptors and their impact on tumorigenesis (or the effect of drugs) can be studied. Such detailed localization analysis can be achieved using super-resolution microscopy approaches.

We have added new text and several references to the "context" section of the paper:

"We used mCitrine to perform SMLM of the growth factor ErbB3 in A431 epithelial carcinoma cells. A431 cells were chosen for this study in part because of their use in previous studies of the ErbB receptor system[8], and also because they tend to be very flat and form extended areas of membrane in contact with the coverslip, offering good conditions for SMLM. ErbB3 is a member of the epidermal growth factor receptor (EGFR) family, consisting of ErbB1 (EGFR), ErbB2 (also known as HER2), ErbB3, and ErbB4. The organization and dynamics of ErbB receptors is an important topic of study because overexpression and unrestrained activation of this family of receptors is implicated in cancer[9], including breast cancer[10]. Long thought to have no kinase activity, ErbB3 has recently been found to exhibit tyrosine kinase activity and to form homo- and heterodimers with other ErbB receptors[11]. Such hybrid receptor formation between ErbB molecules can amplify signaling, and appears to be an important feature of cancer cells. In particular the ErbB2/ErbB3 heterodimer appears to be important for tumor cell proliferation in certain breast cancers[12], and high ErbB3 levels have been linked with tumor resistance to therapies which target ErbB1 or ErbB2[13].

Given the importance of ErbB3 in cancer, an understanding of its organization and dynamics in the plasma membrane of tumor cells is critical. Super-resolution microscopy using single molecule localization reveals the coordinates of each ErbB3 receptor which is tagged with a YFP molecule. This data allows one to explore parameters such as clustering tendencies, an approach used successfully in studies of the T-cell receptor[14]."

3) The authors should state what pH of MEA sample buffer was used? This has been shown to be of critical importance for blinking of probes. Has this been optimized in the current experiment, or reported elsewhere?

The Mowiol-MEA solution had a pH of 8.5. This was not optimized in the current experiments.

4) Is the image acquisition widefield fluorescence or TIRF? If widefield (as the objective lens suggests) how is the out of focus light dealt with by the ThunderSTORM/SOFI algorithms.

All of the presented data was acquired in widefield mode (not TIRF). ThunderSTORM achieves an optical sectioning effect by removing molecules which lie outside the expected range for their FWHM. This results in a "computational optical section" of about 400 nm thickness, approximated by the depth of focus of the objective. SOFI achieves good background suppression because non-fluctuating background (including out of focus light) is suppressed by calculating higher order cumulants over time. SOFI also achieves quite good optical sectioning, as has been shown in the literature [15].

5) Relating to Line 98 and Table 3 - can the authors comment on whether the 'photoelectrons per A/D count' vary with exposure or EM gain on this detector?

This value (photoelectrons per A/D count) does not change with EM gain or with exposure time. It can be changed by adjusting the values of "preamp setting" and the digitizer speed. This information is available from the camera manufacturer. The relevant information needed for the settings we used is included in Table 2.

6) Use of zoomed insets within the images in the figures would help the reader to better see the impact the super-resolution has.

Zoomed-in views have been provided in a new figure (Fig 6). This figure compares the results of SMLM and SOFI analysis.

7) Can the authors explain the source of the variety in numbers for each of the 10 molecules featured in Table 1, for the non-specialist reader (e.g. the localization uncertainly varies between 14.60 and 29.85 - why)?

We have added the following text to the manuscript on line 196: "The variation in parameters between molecules is usually attributed to differences in the local environment of each molecule such as oxygen concentration, and to factors such as the fluorophore orientation."

8) Fig 3B - the red dots are difficult to discern in the compressed version available to the reviewers.

Unfortunately the PDF automatically generated by the manuscript submission system resulted in very low quality images. Please use the links within the PDF (located on the pages with the figures) to download the original high resolution images.

9) The authors should explain what is meant by 2nd, 3rd, 4th order of SOFI (e.g. Line 176 and Fig 5).

SOFI relies on calculation of higher order cumulants. 2nd, 3rd, 4th order SOFI refers to 2nd, 3rd, 4th cumulant that was used for the calculation of the respective SOFI image. We added additional description to clarify this notation in the paper.

We added the following text and references to the manuscript:

"SOFI relies on calculating higher order cumulants as described in the previous section. Calculating cumulants raises the molecular brightness to the n-th power (Eq. 3). The SOFI's non-linear response to brightness becomes an issue for cumulants of higher than second order where fluorescent spots of high brightness may mask less bright details. The balanced SOFI (bSOFI) algorithm linearizes the response to brightness [16] or to the detected intensity [17]. Throughout this work, the "n-th order bSOFI image" refers to an image calculated using the n-th order cumulant and applying the subsequent linearization according to the procedure described in [17]."

10a) Do the density maps (Fig. 1D cf. Fig. 5D) use the same color scaling (the compression on Fig. 5 obscures the scale/numbers)

We changed the density maps so that now they all use the same scaling and color mapping.

10b)- can the authors comment on the significance of the regions of high density erbB3?

We do not wish to speculate about regions with higher densities of ErbB3 without additional data and a larger study of ErbB3 signaling and membrane dynamics.

11) Can the authors comment on how many frames they recommend to accurately reconstruct an image of a sample such as this, given the differences seen between datasets in Table 2? Why do we see such differences in density of detections per frame between YFP data 1 and YFP data 3, what was done differently (similar number of detections, but 10,000 frames and 1,419 frames respectively)? Are the outcomes comparable?

Our experiments revealed some unexpected variation from cell to cell. Nothing was done differently between the different datasets and we have no evidence to explain the variations in the rates of blinking and bleaching. The datasets were truncated somewhat arbitrarily after the blinking had become negligible (just a few visible blinks per frame). We assume that this indicates that all of the molecules capable of blinking had been recorded and that all the information about the positions of ErbB3 molecules had been extracted. There is no way for us to predict how many frames are required for this to occur. Recent work in the literature has examined the question of how many localizations are required for various types of samples[18].

Minor comments

12) Line 20: for the non-specialist reader, I suggest giving a value for the 'classic limit in optical microscopes'

We added a typical value as requested.

13) Line 42: I suggest displaying the equation for lateral resolution as an equation rather than in the text.

We changed the formatting of the equation as requested.

14) Line 153: Typo "Fig. 4D" should be 1D?

We corrected this error.

15) Tables 2 & 3 could be combined and should be referred to from the "Single molecule microscopy" section, beginning Line 80.

We have combined the two tables.

Reviewer #2:

1) The problem with YFP photoblinking is its reliability when compared to other fluorophores such as mEos2 for PALM or Cy5/AF647 for dSTORM. It might not become clear to readers why one should use YFP for SMLM or why raw data with YFP is needed. This should be explained in more detail.

Reviewer #1 had a similar concern and we use the same explanation here. The reason we used this particular fluorescent protein for the presented data is its low photon emission rate which creates a challenge for localization of emitters by SMLM techniques. The aim of our work was to offer a dataset with difficult SMLM images (low SNR) for evaluation of analytical tools under suboptimal conditions. However mCitrine is probably not the best choice for SMLM experiments when compared to newer fluorescent proteins such as mEos. On the other hand, SMLM experiments using mCitrine can be accomplished with a single laser, simplifying the microscope setup.

We added the following text and references to the manuscript:

"The advantage of using mCitrine is that SMLM can be accomplished with a single laser, rather than with separate activation and readout lasers as is done when using mEOS[5]. The question of how fluorophore photophysics influences SMLM experiments is still under investigation[6], but this topic has recently been reviewed fairly comprehensively, taking into account the photoswitching characteristics of fluorescent proteins for SMLM[7]."

2) In the abstract the authors mentioned the low amount of photons when using YFP. In addition to that I also think that the rate of photobleaching is a problem as well (cf. 'YFP data 4.tif' after 1000 frames). How big might be the fraction of YFP molecules that can be reversibly photoswitched? Does the large fraction of bleaching allow reliable reconstruction of the underlying structure while the majority is irreversible photobleached in the beginning of the experiment?

It is not clear that the YFP molecules are irreversibly photobleached. We feel the opposite is the case, that upon an initial large dose of excitation light and under the right buffer conditions (exclusion of oxygen and addition of 100 mM reducing agents), a large fraction of the molecules are converted to a non-fluorescent dark state. These molecules later return to a fluorescent state, resulting in a blinking event. Based on the data at hand, we don't feel confident speculating on the fraction of YFP molecules permanently photobleached vs. those that convert to a dark state and subsequently blink.

Based on our experience in this field, we believe that the presented SMLM and SOFI reconstructions are reliable, but there is no way to assess this within the scope of the present study.

Because raw data from SMLM experiments has not been published up to this point (that we are aware of), our paper allows exploration of this and other topics which are normally not considered at all. We feel this is a strength of our submission to *GigaScience*. On the other hand we agree that mCitrine is not the best choice for SMLM experiments in comparison to other probes. The data presented here is meant as a sort of "wort case scenario" for development of SMLM algorithms.

3) A comparison with another fluorophore, which is known to be very reliable such as Cy5 under dSTORM conditions, should be performed. This would not only help to verify and classify the YFP data for interested users, it would also make the data set more comprehensive for software developers and those who want to learn the method.

We have added an additional dataset that was acquired using Alexa 532 and dSTORM protocols. In this dataset we used 5-fluorouridine to label newly synthesized mRNA transcripts in the nucleus of HeLa cells. We used immunocytochemistry methods to detect the fluorouridine with an antibody, followed by a secondary antibody labeled with Alexa 532. This work was part of a larger study of replication and transcription in the nucleus[4]. This dataset is presented only for the purposes of comparing

photoblinking characteristics and SMLM reconstruction for mCitrine vs. Alexa 532. The Alexa 532 dataset was acquired with the same microscope setup and detector as the mCitrine data, allowing detailed comparisons.

We admit that this is not an ideal choice of sample because it introduces another cell line and biological system, but after looking through our dSTORM data we determined that this was among the highest quality datasets we had available and for which we knew all of the relevant parameters. However the purpose of the paper is to publish the raw and analyzed datasets, not to make observations about any particular biological problem.

4) The authors did SMLM and SOFI analysis of their data, but without comparing both.

We have added a new figure, (Fig 6) which compares the results of SMLM and SOFI analysis. We have also added new text on lines ~200-225 describing the new figure and comparing the results.

5a) It is quite impressive that the authors get 4th order cumulant SOFI images from the data with that much photobleaching. In Fig. 5, what is the difference in resolution between A, B and C?

The difference is the SOFI cumulant order which was used. This has been clarified in the text, and additional details about the SOFI results have been added.

5b) In addition to that, is it possible for the reader of this paper to access the software used (maybe upon personal request)?

The SOFI analysis software may be requested from the authors. ThunderSTORM software is open source and publicly available as noted in the manuscript.

6a) Are there any biological conclusions the author can draw from their images?

The goal of the paper is to offer raw and analyzed single molecule localization microscopy data. Without a larger and more complete study, we are reluctant to draw any biological conclusions about the ErbB3 receptor.

6b) Does SMLM allow different insights into the ErbB3 distribution than SOFI?

SMLM and SOFI offer different but complementary information. Looking at the new Fig 6, the distributions of molecules are visually similar.

6c) Further, how can the quantitative analysis be used to obtain insights?

Future studies may include experiments in which reagents are applied to the cell (for example reagents which perturb the plasma membrane, affect cell signaling, or bind to the ErbB3 receptor), followed by SMLM experiments which would reveal potential changes in ErbB3 clustering. This receptor clustering data could then be correlated with biochemical assays of cell signaling.

6d) It is not clear why the authors used super-resolution microscopy to study ErbB3.

Reviewer #1 had a similar concern and we repeat part of the same reply here.

As seen in Figure 1, diffraction limited microscopy provides only coarse details about the distribution of ErbB3 molecules on these cells. SMLM uncovers potential clustering of ErbB3 in these cells. For example, in the future, correlation of tumorigenic potential of ErbB3 in clusters with its randomly distributed counterparts can be studied. ErbB3 co-localization with downstream effectors, and many other open questions related to the nanoscopic organization of oncogenic receptors and their impact on tumorigenesis (or the effect of drugs) can be studied. Such detailed localization analysis can be achieved using super-resolution microscopy approaches. We have added new text and several references to the "context" section of the paper as noted in the reply to Reviewer #1.

Minor comments:

- Page 5, line 54: It could be mentioned, that the reconstructed image is of artificial nature and with an 100 fold increase in the pixel size if compared to the source image (e.g. from 100 nm source pixel size to 10 nm pixel size).

We have added a sentence to better explain this in line ~57.

- Page 6, line 78: What was the amount of mowiol and the pH of the MEA solution?

The Mowiol-MEA solution had a pH of 8.5. The mowiol was prepared according to standard procedures, resulting in a moviol concentration of about 3.2 mM.

- Fig 2: 'number of molecules', because molecules can blink multiple times I suggest using 'number of localizations'

We changed this as requested.

- Table 2: The term 'Loc. accuracy' is used. Because the real position of the molecule remain known, it is better to use 'localization precision'.

This has been changed to "localization uncertainty," which is the value that is calculated.

- Page 10, line 170 / Figure 3B: The density in the image is by far too high for SMLM. The fraction of artifacts will be very high in this example. An image with appropriate density should selected.

We have selected an image with lower density for Fig. 3B.

- Fig 5E: a monoexponentional approximation might not be suited.

More complex models are possible however in our experience processing the data in subsequences and weighting the frames by inverse values of a monoexponential fit as described in Deshout et al [17] provides satisfactory suppression of the photobleaching effect.

- Page 11, line 190: I disagree, that the results from ThunderSTORM should be taken as ground truth because any localization software is prone to artifacts, especially when low performance fluorophores are used for SMLM such as YFP. It should be more considered as a reference obtained from one of the best open access software packages (ThunderSTORM).

We have re-phrased this section to indicate that the results from ThunderSTORM can be taken as "reference data." A new supplement shows how this can be done. We have changed the text as follows:

"Since the true positions of the molecules remain unknown, the results from ThunderSTORM may be taken as the reference data for comparison purposes. ThunderSTORM offers an analysis tool which compares reference data and experimental data and computes several quantities which can be used to evaluate algorithm performance."

- Ref 24: there might be a typo in the reference.

This has been corrected.

- ThunderSTORM: Was the multi-spot emitter function enabled?

This feature was not used in SMLM analysis.

References

[1] Křížek P, Raška I, Hagen GM. Minimizing detection errors in single molecule localization microscopy. Opt Express 2011;19:3226–35. doi:10.1364/OE.19.003226.

[2] Ovesný M, Křížek P, Borkovec J, Švindrych Z, Hagen GM. ThunderSTORM: A comprehensive ImageJ plug-in for PALM and STORM data analysis and super-resolution imaging. Bioinformatics 2014;30. doi:10.1093/bioinformatics/btu202.

[3] Ovesný M, Křížek P, Borkovec J, Švindrych Z, Hagen GM. Image analysis for single-molecule localization microscopy. In: Diaspro A, Marc A. M. J. van Zandvoort, editors. Super-Resolution Imaging Biomed., Boca Raton, Florida: CRC Press; 2016, p. 79–97.

[4] Smirnov E, Borkovec J, Kováčik L, Svidenská S, Schröfel A, Skalníková M, et al. Separation of replication and transcription domains in nucleoli. J Struct Biol 2014;188:259–66. doi:10.1016/j.jsb.2014.10.001.

[5] Betzig E, Patterson GH, Sougrat R, Lindwasser OW, Olenych S, Bonifacino JS, et al. Imaging intracellular fluorescent proteins at nanometer resolution. Science 2006;313:1642–5.

[6] Pennacchietti F, Gould TJ, Hess ST. The role of probe photophysics in localization-based superresolution microscopy. Biophys J 2017;113:2037–54. doi:10.1016/j.bpj.2017.08.054.
[7] Shcherbakova DM, Sengupta P, Lippincott-Schwartz J, Verkhusha V V. Photocontrollable fluorescent proteins for superresolution imaging. Annu Rev Biophys 2014;43:303–29. doi:10.1146/annurev-biophys-051013-022836.
[8] Nagy P, Arndt-Jovin DJ, Jovin TM. Small interfering RNAs suppress the expression of endogenous and GFP-fused epidermal growth factor receptor (erbB1) and induce apoptosis in erbB1-overexpressing cells. Exp Cell Res 2003;285:39–49.
[9] Yarden Y, Sliwkowski. Untangling the ErbB signaling network. Nat Rev Mol Cell Biol 2001;2:127–37.
[10] Naidu R, Yadav M, Nair S, Kutty MK. Expression of c-erbB3 protein in primary breast carcinomas. Br J Cancer 1998;78:1385–90.
[11] Steinkamp MP, Low-Nam ST, Yang S, Lidke KA, Lidke DS, Wilson BS. ErbB3 is an active tyrosine kinase capable of homo- and heterointeractions. Mol Cell Biol 2014;34:965–77. doi:10.1128/MCB.01605-13.
[12] Holbro T, Beerli RR, Maurer F, Koziczak M, Barbas CF, Hynes NE. The ErbB2/ErbB3 heterodimer functions as an oncogenic unit: ErbB2 requires ErbB3 to drive breast tumor cell proliferation. Proc Natl Acad Sci U S A 2003;100:8933–8938. doi:10.1073/pnas.1537685100.
[13] Sithanandam G, Anderson LM. The ErbB3 receptor in cancer and cancer gene therapy. Cancer Gene Ther 2008;15:413–48. doi:10.1038/cgt.2008.15.
[14] Williamson DJ, Owen DM, Rossy J, Magenau A, Wehrmann M, Gooding JJ, et al. Pre-existing clusters of the adaptor Lat do not participate in early T cell signaling events. Nat Immunol 2011;12:655–62.
[15] Dertinger T, Xu J, Naini O, Vogel R, Weiss S. SOFI-based 3D superresolution sectioning with a widefield microscope. Opt Nanoscopy 2012;1:2. doi:10.1186/2192-2853-1-2.
[16] Geissbuehler S, Bocchio NL, Dellagiacoma C, Berclaz C, Leutenegger M, Lasser T. Mapping molecular statistics with balanced super-resolution optical fluctuation imaging (bSOFI). Opt Nanoscopy 2012;1:4.
[17] Deschout H, Lukes T, Sharipov A, Szlag D, Feletti L, Vandenberg W, et al. Complementarity of PALM and SOFI for super-resolution live-cell imaging of focal adhesions. Nat Commun 2016;7:13693. doi:10.1038/ncomms13693.
[18] Fox-Roberts P, Marsh R, Pfisterer K, Jayo A, Parsons M, Cox S. Local dimensionality determines imaging speed in localization microscopy. Nat Commun 2017;8:13558. doi:10.1038/ncomms13558.
