# Supplementary material for: Quantitative super-resolution single molecule microscopy dataset of YFP-tagged growth factor receptors
Source: Gigascience. 2018 Jan 19;7(3):1–10. doi: 10.1093/gigascience/giy002 (PMC5841371; doi:10.1093/gigascience/giy002)
Supplement: Supplemental material [file giy002_supp.pdf]

Example of data re-use: quantitative evaluation of SMLM algorithms using ThunderSTORM

1.1 Introduction

ThunderSTORM [1] can compare the results of two SMLM analysis methods by matching, within a user-selected tolerance, each localization in a results table with the nearest corresponding molecule in a second results table. In this way a new algorithm can be tested against a standard method. Typically this is done with simulated data, but this approach can be used with experimental data as well. The localization microscopy datasets presented here can be used to develop, evaluate, and validate new SMLM algorithms. When working with simulated data, the true positions of the simulated molecules are known (ground-truth positions). When working with experimental data we must use the results from a standard algorithm as the reference data and test this against the results of an algorithm under development. In ThunderSTORM, the reference dataset is always called the "ground-truth" data.

1.2 Counting localized and missed molecules

The process of performance evaluation starts by pairing the localized molecules with the closest molecule in the ground-truth data (or the reference data set), see Figure 1. The numbers of correctly and incorrectly identified molecules are counted as follows. If the distance between the paired molecules is smaller than a user-specified radius, then the localization is counted as a true positive (TP) detection and the localized molecule is associated with the ground-truth position. If the distance is greater than or equal to that radius, then the localization is counted as a false positive (FP) detection. Ground-truth molecules which were not associated with the localized molecules are counted as false negatives (FNs). With a growing density of molecules it becomes more important how the algorithm performs the matching. To solve the problem of finding the correct matching between localized molecules and the ground-truth data, we use the Gale-Shapley algorithm [2].

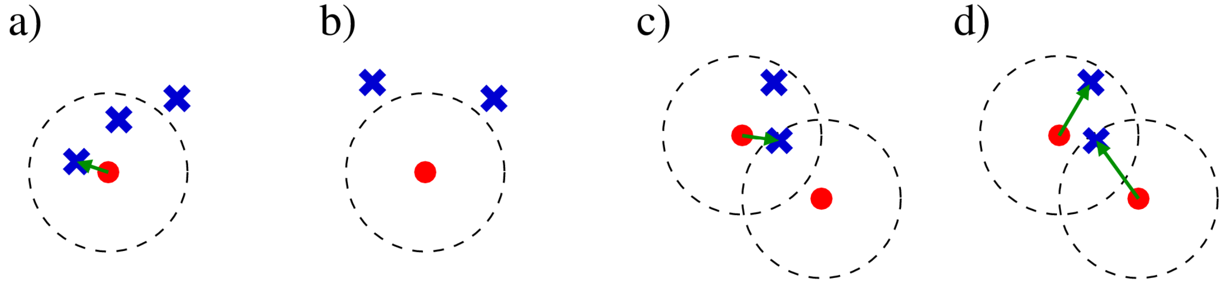

Figure 1. Counting localized and missed molecules. Red dot - ground-truth position of a molecule, blue cross - localized molecule, green arrow - association of a localized molecule with ground-truth position, dashed circle - detection tolerance radius. a) 1 TP + 1 FP, b) 1 FN + 2 FP, c-d) example of a situation, where c) greedy approach fails by finding 1 TP + 1 FP + 1 FN, and where d) Gale-Shapley algorithm finds a correct solution with 2 TP.

1.3. Precision and recall

Statistical measures related to the number of correctly or incorrectly detected molecules, or missed molecules, are the recall (r) (also called sensitivity) and the precision (p) (also called positive predictive value) [3–5]. Their definitions are given by

$$r = TP / TP + FN$$
$$p = TP / TP + FP \qquad (1)$$

Recall measures the fraction of correctly identified molecules, and precision measures the portion of correctly identified molecules out of the set of all localizations. The theoretical optimum is achieved for values of recall and precision both equal to 1.0.

1.4 F1 score

For purposes of comparison between multiple algorithms, it is convenient to combine precision and recall into a single measure of performance with some trade-offs between both values. A traditional method for this applies the F1 score [3,4], defined as

$$F1 = 2pr / p + r \qquad (2)$$

Values of the F1 score close to zero indicate both bad recall and precision while values approaching 1.0 signify a good ratio between recall and precision.

1.5 Jaccard index

Another measure suitable for comparing similarity and diversity of sets of samples is the Jaccard index defined by the formula

$$J = \frac{|A \cap B|}{|A \cup B|}. \qquad (3)$$

Here A is the set of ground-truth molecular positions, B is the set of all molecular positions localized by processing the data, intersection $|A \cap B| = TP$ gives the number of true positive detections, union $|A \cup B| = TP + FP + FN$. The Jaccard index ranges from zero to one and a theoretical optimum is achieved for values of the Jaccard index equal to 1.0.

1.6 RMS distance

For all molecules identified as true positives, we also calculate the root-mean square distance between the ground-truth positions of the molecules and their localizations.

1.7 Performance Evaluation

If ground truth molecular positions are available, ThunderSTORM can evaluate the performance of different processing methods. Ground truth data can be created by ThunderSTORM's generator of simulated data, or imported from a file by selecting `Plugins → ThunderSTORM → Import/Export → Import ground-truth`. To start evaluation, select `Plugins → ThunderSTORM → Performance testing → Performance evaluation`, enter the tolerance radius for accepting true-positive detections, and click `OK`. The results indicate true-positive detections (green), false positive detections (red), false negatives (orange), and related statistical measures (e.g., recall, precision, F1 score).

a) b) c)

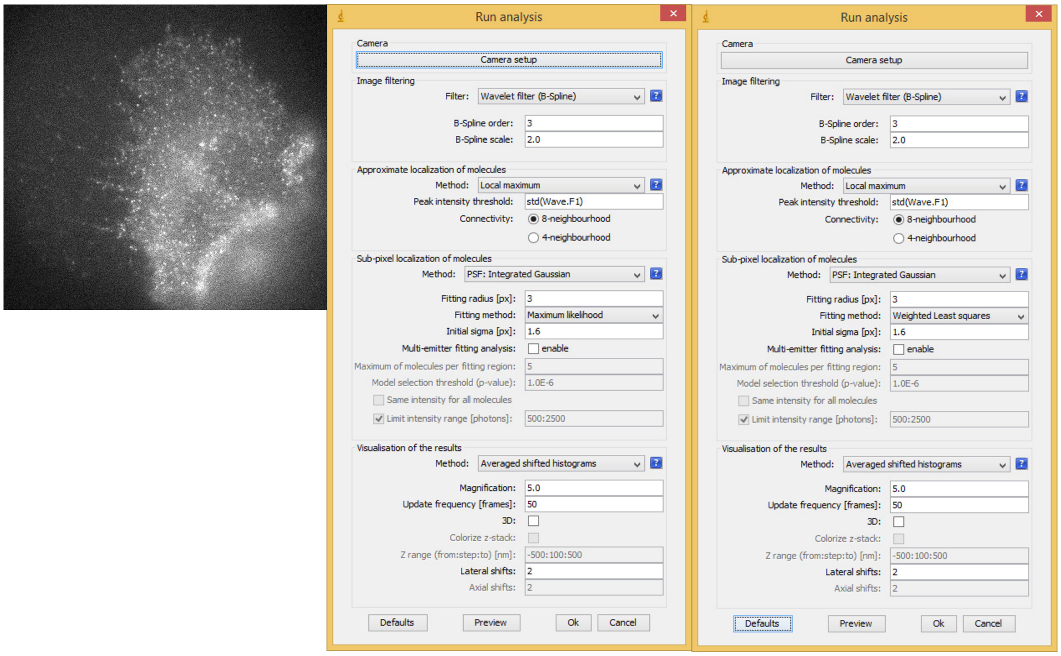

Figure 2. a) input image (frame 100 of 'YFP dataset 2'), b) ThunderSTORM setup, default settings with maximum likelihood fitting method selected, c) ThunderSTORM setup, default settings with weighted least squares fitting method selected.

a) b)

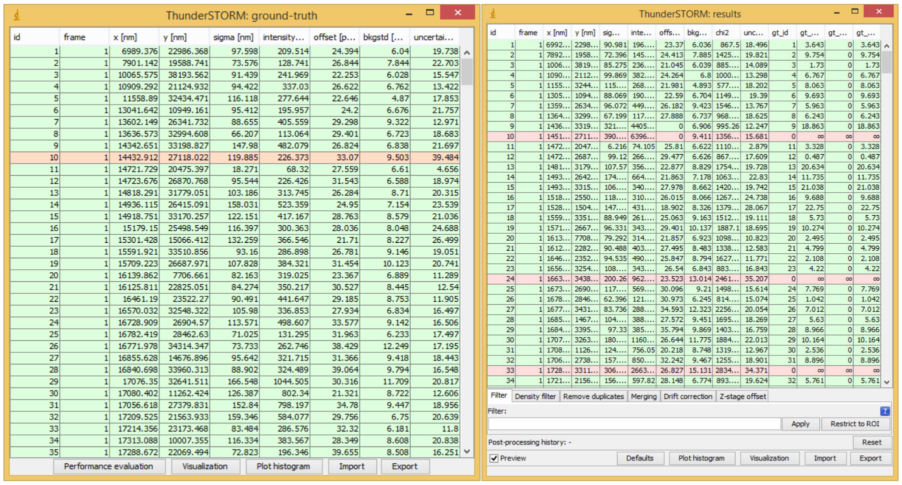

c)

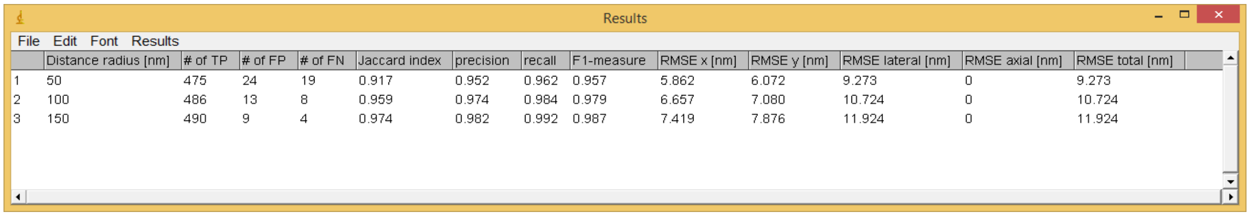

	Distance radius [nm]	# of TP	# of FP	# of FN	Jaccard index	precision	recall	F1-measure	RMSE x [nm]	RMSE y [nm]	RMSE lateral [nm]	RMSE axial [nm]	RMSE total [nm]
1	50	475	24	19	0.917	0.952	0.962	0.957	5.862	6.072	9.273	0	9.273
2	100	486	13	8	0.959	0.974	0.984	0.979	6.657	7.080	10.724	0	10.724
3	150	490	9	4	0.974	0.982	0.992	0.987	7.419	7.876	11.924	0	11.924

Figure 3. a) ThunderSTORM results table using maximum likelihood fitting. b) ThunderSTORM results table using weighted least squares fitting. The results indicate true-positive detections (green), false positive detections (red), and false negatives (orange). c) Table of results when varying the molecule matching tolerance. Statistics are calculated which quantitatively compares the two results tables.

References

[1] Ovesný M, Křížek P, Borkovec J, Švindrych Z, Hagen GM. ThunderSTORM: A comprehensive ImageJ plug-in for PALM and STORM data analysis and super-resolution imaging. Bioinformatics 2014;30.

[2] Gale D, Shapley LS. College Admissions and the Stability of Marriage. Am Math Mon. 1962;69:9–15.

[3] Tan P-N, Steinbach M, Kumar V. Introduction to data mining. Pearson Addison Wesley; 2005.

[4] Křížek P, Raška I, Hagen GM. Minimizing detection errors in single molecule localization microscopy. Opt Express. 2011;19:3226–35.

[5] Wolter S, Löschberger A, Holm T, Aufmkolk S, Dabauvalle M-C, van de Linde S, et al. rapidSTORM: accurate, fast open-source software for localization microscopy. Nat Methods. 2012;9:1040–1.
