# Supplementary material for: Quantitative super-resolution single molecule microscopy dataset of YFP-tagged growth factor receptors
Source: Gigascience. 2018 Jan 19;7(3):1–10. doi: 10.1093/gigascience/giy002 (PMC5841371; doi:10.1093/gigascience/giy002)
Supplement: Response_to_Reviewer_Comments_Original_Submission.pdf [file giy002_response_to_reviewer_comments_original_submission.pdf]

Author's Response To Reviewer Comments

please see attached
