# Supplementary material for: Quantitative super-resolution single molecule microscopy dataset of YFP-tagged growth factor receptors
Source: Gigascience. 2018 Jan 19;7(3):1–10. doi: 10.1093/gigascience/giy002 (PMC5841371; doi:10.1093/gigascience/giy002)
Supplement: Reviewer_1_Report_(Original_Submission) -- Graham Wright — 25 Sep 2017 Reviewed [file giy002_reviewer_1_report_(original_submission).pdf]

Reviewer Report

Title: Quantitative super-resolution single molecule microscopy dataset of YFP-tagged growth factor receptors

Version: Original Submission **Date:** 25 Sep 2017

Reviewer name: Graham Wright

Reviewer Comments to Author:

The authors present four superresolution single molecule localisation microscopy (SMLM) datasets utilising YFP (specifically mCitrine) tagged erbB3 in A431 cells - a welcome addition to the literature and suitable for Giga Science. mCitrine is a challenging fluorophore for SMLM and hence such datasets provide a good testing ground for the continuing development of SMLM algorithms. The paper should be acceptable for publication in Giga Science with some minor corrections/revisions/suggestions as outlined here:

1) Can the authors comment on where mCitrine sits, relative to other fluorescent proteins, in terms of an appropriate choice for the purpose of SMLM? What parameters should be considered?
2) In the 'Context' section (paragraph from Line 56), can the authors expand on why A431 cells and erbB3 were used and what their biological significance is? Why might superresolution be a useful approach for studying erbB3 - what extra information can be gained from such approaches when compared to diffraction limited techniques and can we see this in the images presented?
3) The authors should state what pH of MEA sample buffer was used? This has been shown to be of critical importance for blinking of probes. Has this been optimized in the current experiment, or reported elsewhere?
4) Is the image acquisition widefield fluorescence or TIRF? If widefield (as the objective lens suggests) how is the out of focus light dealt with by the ThunderSTORM/SOFI algorithms.
5) Relating to Line 98 and Table 3 - can the authors comment on whether the 'photoelectrons per A/D count' vary with exposure or EM gain on this detector?
6) Use of zoomed insets within the images in the figures would help the reader to better see the impact the superresolution has.
7) Can the authors explain the source of the variety in numbers for each of the 10 molecules featured in Table 1, for the non-specialist reader (e.g. the localization uncertainly varies between 14.60 and 29.85 - why)?
8) Fig 3B - the red dots are difficult to discern in the compressed version available to the reviewers.
9) The authors should explain what is meant by 2nd, 3rd, 4th order of SOFI (e.g. Line 176 and Fig 5).
10) Do the density maps (Fig. 1D cf. Fig. 5D) use the same colour scaling (the compression on Fig. 5 obscures the scale/numbers) - can the authors comment on the significance of the regions of high density erbB3?
11) Can the authors comment on how many frames they recommend to accurately reconstruct an image of a sample such as this, given the differences seen between datasets in Table 2? Why do we see such differences in density of detections per frame between YFP data 1 and YFP data 3,

what was done differently (similar number of detections, but 10,000 frames and 1,419 frames respectively)? Are the outcomes comparable?

Minor comments
12) Line 20: for the non-specialist reader, I suggest giving a value for the 'classic limit in optical microscopes'
13) Line 42: I suggest displaying the equation for lateral resolution as an equation rather than in the text.
14) Line 153: Typo "Fig. 4D" should be 1D?
15) Tables 2 & 3 could be combined and should be referred to from the "Single molecule microscopy" section, beginning Line 80.

Level of Interest

Please indicate how interesting you found the manuscript: An article of importance in its field

Quality of Written English

Please indicate the quality of language in the manuscript: Acceptable

Declaration of Competing Interests

Please complete a declaration of competing interests, considering the following questions:

- Have you in the past five years received reimbursements, fees, funding, or salary from an organisation that may in any way gain or lose financially from the publication of this manuscript, either now or in the future?
- Do you hold any stocks or shares in an organisation that may in any way gain or lose financially from the publication of this manuscript, either now or in the future?
- Do you hold or are you currently applying for any patents relating to the content of the manuscript?
- Have you received reimbursements, fees, funding, or salary from an organization that holds or has applied for patents relating to the content of the manuscript?
- Do you have any other financial competing interests?
- Do you have any non-financial competing interests in relation to this paper?

If you can answer no to all of the above, write 'I declare that I have no competing interests' below. If your reply is yes to any, please give details below.

I declare that I have no competing interests

I agree to the open peer review policy of the journal. I understand that my name will be included on my report to the authors and, if the manuscript is accepted for publication, my named report including any attachments I upload will be posted on the website along with the authors' responses. I agree for my report to be made available under an Open Access Creative Commons CC-BY license (http://creativecommons.org/licenses/by/4.0/). I understand that any comments which I do not wish to be included in my named report can be included as confidential comments to the editors, which will not be published.

I agree to the open peer review policy of the journal

To further support our reviewers, we have joined with Publons, where you can gain additional credit to further highlight your hard work (see: https://publons.com/journal/530/gigascience). On publication of this paper, your review will be automatically added to Publons, you can then choose whether or not to claim your Publons credit. I understand this statement.

Yes
