# Supplementary material for: Quantitative super-resolution single molecule microscopy dataset of YFP-tagged growth factor receptors
Source: Gigascience. 2018 Jan 19;7(3):1–10. doi: 10.1093/gigascience/giy002 (PMC5841371; doi:10.1093/gigascience/giy002)
Supplement: Reviewer_2_Report_(Original_Submission) -- Sebastian Van de Linde — 13 Oct 2017 Reviewed [file giy002_reviewer_2_report_(original_submission).pdf]

Reviewer Report

Title: Quantitative super-resolution single molecule microscopy dataset of YFP-tagged growth factor receptors

Version: Original Submission **Date:** 13 Oct 2017

Reviewer name: Sebastian Van de Linde

Reviewer Comments to Author:

In their manuscript Lukes and coworkers present a single-molecule based super-resolution microscopy dataset employing YFP blinking. Acquisition and gerneration of the data is clearly described and the need for such data is given.
The data were analysed with two different techniques, single-molecule localization microscopy (SMLM) and superresolution optical fluctuation imaging (SOFI). The software, ThundeSTORM, that was used for SMLM analysis is available and well-established.

I aggree with the authors that full raw data sets are, unfortunately, not wideley available and also that data of lower quality (when for instance compared to Cy5) should be accessible as well especially for improving software for data analysis.
The manuscript is of interest for the interested SMLM user and software developer. I have several comments that should be addressed before publication.

1) The problem with YFP photoblinking is its reliability when compared to other fluorophores such as mEos2 for PALM or Cy5/AF647 for dSTORM. It might not become clear to readers why one should use YFP for SMLM or why raw data with YFP is needed. This should be explained in more detail.

2) In the abstract the authors mentioned the low amount of photons when using YFP. In addition to that I also think that the rate of photobleaching is a problem as well (cf. 'YFP data 4.tif' after 1000 frames). How big might be the fraction of YFP molecules that can be reversibly photoswitched? Does the large fraction of bleaching allow reliable reconstruction of the underlying structure while the majority is irreversible photobleached in the beginning of the experiment?

3) A comparison with another fluorophore, which is known to be very reliable such as Cy5 under dSTORM conditions, should be performed. This would not only help to verify and classify the YFP data for interested users, it would also make the data set more comprehensive for software developers and those who want to learn the method.

4) The authors did SMLM and SOFI analysis of their data, but without comparing both.

5) It is quite impressive that the authors get 4th order cumulant SOFI images from the data with that much photobleaching. In Fig. 5, what is the difference in resolution between A, B and C? In addition to

that, is it possible for the reader of this paper to access the software used (maybe upon personal request)?

6) Are there any biological conclusions the author can draw from their images? Does SMLM allow different insights into the erbB3 distribution than SOFI? Further, how can the quantitative analysis be used to obtain insights? It is not clear why the authors used super-resolution microscopy to study erbB3.

Minor comments:

- Page 5, line 54: It could be mentioned, that the reconstructed image is of artificial nature and with an 100 fold increase in the pixelsize if compared to the source image (e.g. from 100 nm source pixel size to 10 nm pixel size).

- Page 6, line 78: What was the amount of mowiol and the pH of the MEA solution?

- Fig 2: 'number of molecules', because molecules can blink multiple times I suggest using 'number of localizations'

- Table 2: The term 'Loc. accurracy' is used. Because the real position of the molecule remain known, it is better to use 'localization precision'.

- Page 10, line 170 / Figure 3B: The density in the image is by far to high for SMLM. The fraction of artifacts will be very high in this example. An image with appropiate density should selected.

- Fig 5E: a monoexponentional approximation might not be suited

- Page 11, line 190: I disagree, that the results from ThunderSTORM should be taken as grund truth because any localization software is prone to artifacts, especially when low performance fluorophores are used for SMLM such as YFP. It should be more cosidered as a reference obtained from one of the best open access software packages (ThunderSTORM).

- Ref 24: there might be a typo in the reference

- ThunderSTORM: Was the multi-spot emitter function enabled?

Level of Interest

Please indicate how interesting you found the manuscript: An article of importance in its field

Quality of Written English

Please indicate the quality of language in the manuscript: Acceptable

Declaration of Competing Interests

Please complete a declaration of competing interests, considering the following questions:

- Have you in the past five years received reimbursements, fees, funding, or salary from an organisation that may in any way gain or lose financially from the publication of this manuscript, either now or in the future?
- Do you hold any stocks or shares in an organisation that may in any way gain or lose financially from the publication of this manuscript, either now or in the future?
- Do you hold or are you currently applying for any patents relating to the content of the manuscript?
- Have you received reimbursements, fees, funding, or salary from an organization that holds or has applied for patents relating to the content of the manuscript?
- Do you have any other financial competing interests?
- Do you have any non-financial competing interests in relation to this paper?

If you can answer no to all of the above, write 'I declare that I have no competing interests' below. If your reply is yes to any, please give details below.

I declare that I have no competing interests

I agree to the open peer review policy of the journal. I understand that my name will be included on my report to the authors and, if the manuscript is accepted for publication, my named report including any attachments I upload will be posted on the website along with the authors' responses. I agree for my report to be made available under an Open Access Creative Commons CC-BY license (http://creativecommons.org/licenses/by/4.0/). I understand that any comments which I do not wish to be included in my named report can be included as confidential comments to the editors, which will not be published.

I agree to the open peer review policy of the journal

To further support our reviewers, we have joined with Publons, where you can gain additional credit to further highlight your hard work (see: https://publons.com/journal/530/gigascience). On publication of this paper, your review will be automatically added to Publons, you can then choose whether or not to claim your Publons credit. I understand this statement.

Yes
